# Biomaterials for biomarker imaging and detection

**DOI:** 10.1016/j.jare.2025.07.049

**Published:** 2025-08-07

**Authors:** Yan Wang, Xinyu Huang, Guiying Wu, Wanping Wu, Shuang Li, Chunyu Su, Li Li, Qizhuang Lv

**Affiliations:** a College of Smart Agriculture, Yulin Normal University, Yulin 537000 Guangxi, China; b Guangxi Key Laboratory of Agricultural Resources Chemistry and Biotechnology, Yulin 537000 Guangxi, China

**Keywords:** Biomarkers, Biomaterials, Detection, Imaging, Clinical applications

## Abstract

**Background:**

In the realm of biomedical research and clinical practice, the identification and accurate detection of biomarkers have become increasingly critical. Biomarkers serve as key indicators for disease diagnosis, prognosis evaluation, and drug efficacy monitoring, playing a pivotal role in advancing personalized medicine. However, the complexity and diversity of biomarkers pose significant challenges to their detection and imaging. Traditional methods, such as immunoassays, nucleic acid detection, and mass spectrometry, often fall short in terms of sensitivity, specificity, and efficiency. Consequently, there is an urgent need for more precise and efficient technologies to enhance the detection of biomarkers.

**Aim of review:**

This review aims to provide a comprehensive overview of the latest advancements in biomaterials for biomarker imaging and detection. It seeks to highlight the critical role of biomarkers in disease diagnosis and management, while exploring the potential of newly developed biomaterials to overcome the limitations of conventional detection methods.

**Key scientific concepts of review:**

The review delves into the unique physicochemical properties of biomaterials, such as nanoparticles, quantum dots (QDs), and biopolymers, which enable highly sensitive, specific, and high-resolution biomarker detection. This review finds that by integrating their molecular recognition mechanisms with advanced imaging technologies, these biomaterials demonstrate significant advantages in detecting biomarkers for major diseases such as cancer, cardiovascular diseases (CVDs), and neurodegenerative diseases. For instance, nanoparticle-based probes can detect tumor markers at extremely low concentrations, while QD imaging techniques enable high-resolution imaging at the cellular and tissue levels. Additionally, this review provides an in-depth discussion of the numerous challenges confronting biomaterial-based detection technologies during clinical translation and proposes future research directions. We emphasize the necessity of accelerating the development of innovative materials, optimizing imaging and detection technologies, and facilitating clinical application translation.

## Highlights

• In-depth analysis of the classification, characteristics and application of biomarkers. Overview of biomaterial physicochemical properties and delivery routes.
• Explain in detail the mechanism and advantages of biomaterials in biomarker detection.
• Comprehensive summary of biomaterials' applications in disease - biomarker detection.
• In-depth analysis of the challenges and future development direction of biomaterial detection technology.

## Graphical abstract

**Figure:**
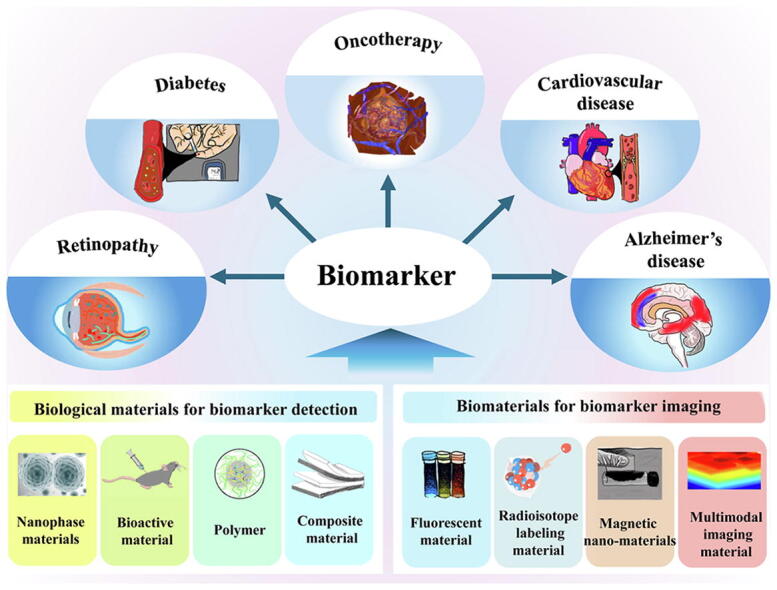

## Introduction

With the advancement of science and technology, the standard of living and quality of life for individuals have significantly improved; however, the diagnosis and treatment of diseases remain essential pillars for maintaining human health and well-being. Currently, disease diagnosis and treatment primarily rely on a combination of traditional clinical observation and modern molecular biology and imaging techniques. Among these, biomarkers play a crucial role as key tools for disease diagnosis and management, garnering increasing attention and research focus. Biomarkers are extensively found in biological samples such as blood, urine, feces, and saliva [1,2], reflecting changes in physiological, biochemical, or pathological processes. They provide essential information for diagnosis, assess the severity or risk of diseases, and guide clinical interventions [3,4].

Traditional methods for biomarker detection and imaging primarily include immunoassays, nucleic acid detection, and mass spectrometry. However, these methods face limitations due to the diversity and complexity of biomarkers; they can be cumbersome, time-consuming, costly, and may exhibit low sensitivity. Consequently, there is a pressing need to develop more efficient and accurate methods for biomarker detection and imaging, with biomaterials offering significant potential to enhance specificity and sensitivity. For example, in situ biomarker imaging provides multidimensional information (e.g., spatial distribution) crucial for demonstrating drug mechanisms in disease processes, requiring high-resolution contrast while being well-suited for in vivo research and diagnostics. Fluorescent probes enable specific biomarker detection [5]. A clear distinction exists between sensing and imaging for biomarker detection: Sensing technology converts biomarker information into physicochemical signals for quantitative analysis, emphasizing signal generation and precise measurement [[6], [7], [8], [9]], whereas imaging focuses on spatial visualization. Specifically, electrochemical metal–organic framework (MOF) sensors leverage the electrochemical properties and specific recognition capabilities of MOFs to detect cancer-related proteins and urinary biomarkers with high sensitivity and selectivity [6,9]. Conversely, nanomaterials-hybridization chain reaction (HCR) strategies target microRNA (miRNA) detection using nucleic acid probes with HCR signal amplification. These systems achieve multiplexed signal outputs with high sensitivity, effectively differentiating miRNA sequences in complex biological samples [8]. Additionally, the diversity and customizability of biomaterials enable their use as drug carriers for the direct treatment of various diseases, thereby advancing the establishment of an individualized medical system. A review of literature related to biomarkers and biological materials (2015 ∼ 2024) in the Web of Science database shows that the number of studies in this field has been on the rise, indicating that this area is becoming increasingly important.

Nevertheless, current research on biomaterials for biomarker imaging and detection is still in its early stages. The transition from basic research to clinical application is complex, with several challenges remaining to be addressed, including biological safety, material stability, and manufacturing processes [10,11]. To address this need, a systematic review of recent applications of biomaterials for biomarker imaging and detection is warranted. First, we summarize the critical role of biomarkers in disease diagnosis, prognosis evaluation, and efficacy prediction, along with the latest advancements in biomarker imaging and detection technologies, highlighting their applications in these domains. Second, we evaluate various biomaterials and their roles in biomarker imaging and detection, such as fluorescent materials, radioisotope-labeled materials, multimodal imaging materials, and other biological materials. We also cover nanomaterials, bioactive materials, composite materials, and multifunctional biomaterials like QDs utilized in biomarker detection. Finally, we discuss the shortcomings of biomaterials in biomarker imaging and detection based on existing literature and explore prospects for the future development of biomarkers and biomaterials. This paper aims to provide researchers and scholars interested in biomarkers and biomaterials with a comprehensive and systematic perspective on the application of biomaterials for biomarker imaging and detection, thereby inspiring new research ideas and promoting further advancements in related fields.

## Biomarkers

Biomarkers are analytes present in biological samples that can predict a patient’s disease status. Over time, the definition of biomarkers has broadened to include any biological measurement, encompassing genomic and proteomic analyses, among others [12]. Biomarkers can be categorized into exposure biomarkers, effect biomarkers, and sensitivity biomarkers, each serving as essential tools in the field of medicine. They play a critical role in disease diagnosis, prognosis evaluation, and efficacy prediction, providing a scientific basis and guidance for clinical decision-making.

### Basic requirements and characteristics of biomarkers

Although numerous studies have been conducted on biomarkers, clear regulations governing their selection principles are still lacking. Prior research has indicated that factors such as small sample sizes, varying screening platforms, and inconsistent biomarker selection can lead to significant bias in results [13]. While there are variations in the basic requirements and characteristics of different biomarkers, they are generally assessed based on six key aspects: specificity, sensitivity, stability, repeatability, individual variability, non-invasiveness, and practicality.

#### Specificity

**Fig. 1:**
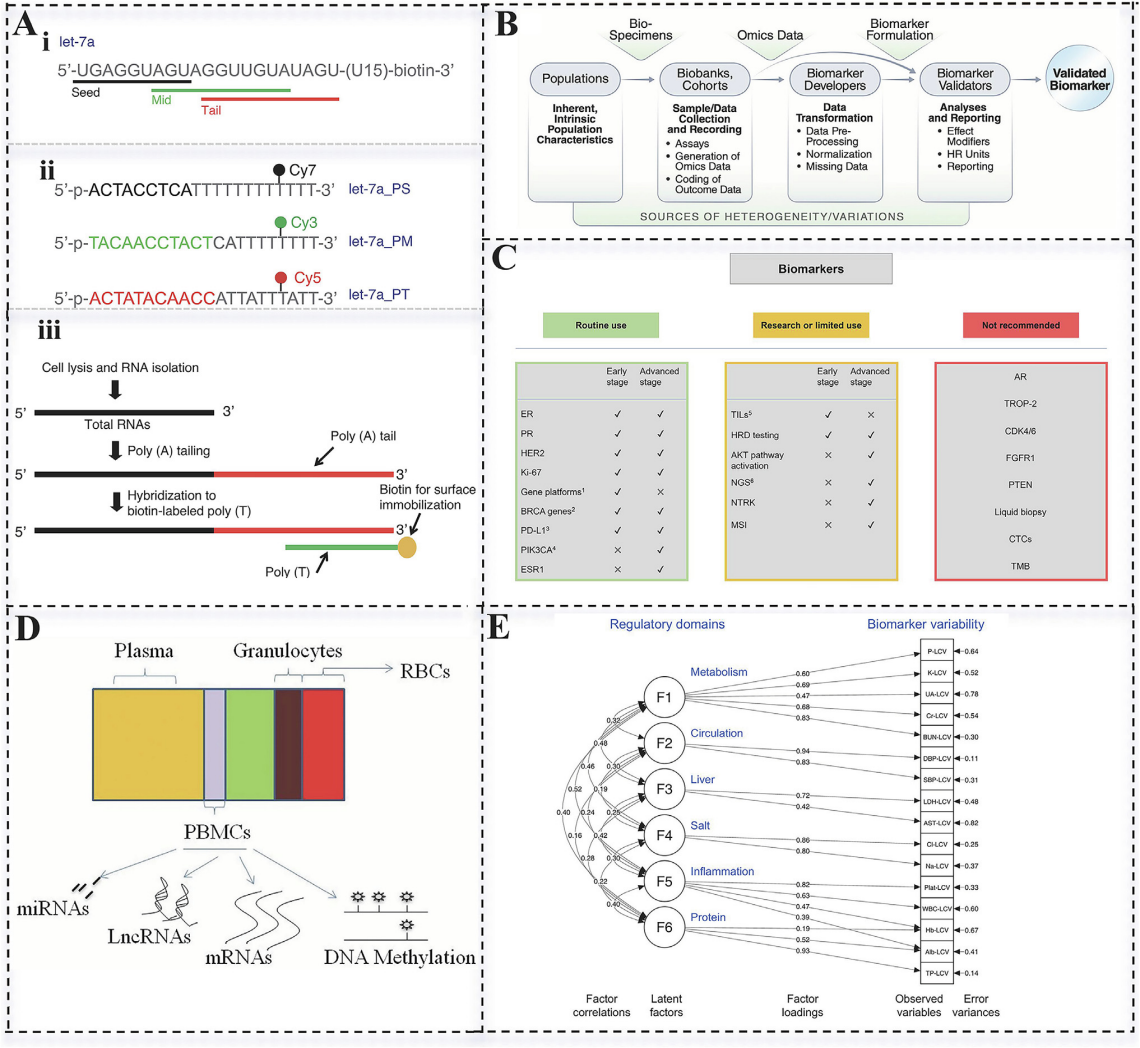
Mirna detection strategies and breast cancer biomarker studies. (a) overview of mirna detection strategies. (i) the target rna was designed with the let-7a sequence at its5′ end, a U15 linker, and biotin labeling at its 3′ end. (ii) DNA probes targeting let-7a, let-7a_PS, let-7a_PM, and let-7a_PT in the seed, middle, and tail regions were labeled with different fluorescent markers for distinction. (iii) Endogenous miRNA immobilization protocol involving total RNA extracted from cell lysates, 3′ end plus tail poly(A) addition, hybridization to complementary DNA strands, and immobilization to the surface via streptavidin–biotin interactions [16]. (B) Sources of variation to consider in biomarker validation studies, including population heterogeneity, sample collection, data processing, analysis, and reporting [26]. (C) Application status of breast cancer biomarkers, categorized into routine application, research application, and non-recommended application [30]. (D) Diversity of PBMCs as a of biomarkers [23]. (E) Path diagram of the six-factor confirmatory factor analysis model, illustrating the relationship between regulatory domains (double arrow path) and the relationship between each domain and biomarker variability (single arrow path). The TE value represents the standardized path coefficient [27].

The specificity of a biomarker primarily refers to its ability to accurately indicate the presence or expression associated with a particular disease, thus distinguishing it from other diseases or normal physiological states. Specific biomarkers are typically grounded in a comprehensive understanding of the molecular mechanisms underlying a disease and reflect biological changes that are unique to that condition. Highly specific biomarkers significantly reduce the risk of misdiagnosis. For instance, miRNAs have emerged as promising biomarkers for early cancer detection, exhibiting distinct expression patterns across different cancer types (Fig. 1A). These specific expression profiles provide critical molecular information for cancer classification, diagnosis, and treatment [[14], [15], [16]].

In clinical applications, the specificity of biomarkers must be comprehensively evaluated based on the specific circumstances of the patient. For example, in detecting the breast cancer marker CA15-3 in serum samples from breast cancer patients, rapid and label-free detection methods using photonic crystal resonance sensors have shown high specificity and sensitivity [17,18]. Consequently, the specificity of biomarkers is a key property in clinical diagnosis, and optimizing detection methods while combining multiple biomarkers can enhance diagnostic accuracy and the reliability of prognosis evaluations. Future studies should continue to focus on identifying high-specificity biomarkers and validating their utility across various diseases.

#### Sensitivity

Sensitivity is a critical measure of a biomarker’s ability to detect a disease, particularly in early diagnosis and disease management. Highly sensitive biomarkers can enhance the accuracy and reliability of diagnoses, which is especially important in the design of clinical trials. Optimizing threshold selection can help balance the size of the positive population and the effect size of the biomarker [19]. For instance, in the diagnosis of Alzheimer’s disease (AD), plasma biomarker strategies have been developed to identify patients most likely to benefit from anti-amyloid immunotherapy. The plasma biomarker p-tau217 demonstrated a strong association with positivity for Amyloid-β (Aβ) and exhibited high sensitivity (AUC = 0.94) in the validation set, highlighting the significant role of highly sensitive plasma biomarkers in reducing the need for invasive diagnostic procedures, such as cerebrospinal fluid (CSF) analysis or positron emission tomography (PET) [20].

However, it is important to note that while biomarkers offer non-invasive or minimally invasive detection methods, their sensitivity can be influenced by various factors, including variations in sample collection and processing, the precision of analytical techniques, and differential marker expression across diverse populations. Additionally, current biomarker detection faces several challenges regarding sensitivity, such as managing false positive results due to insufficient specificity and ensuring the consistency and reliability of biomarkers across different disease stages and subtypes [21]. Therefore, future research should focus on developing more sensitive detection techniques, enhancing the stability and reproducibility of biomarkers, and validating their applicability across various populations through large-scale clinical studies.

#### Stability

The stability of a biomarker refers to its ability to maintain a consistent amount or activity in a biological sample over a certain period under specific conditions. Stability directly impacts the reliability and repeatability of biomarker detection and imaging results and is a critical factor for biomarkers used as clinical diagnostic or research tools [22]. Currently, peripheral blood mononuclear cells (PBMCs) are widely studied as a source of important biomarkers due to their stability and specificity, particularly in various diseases such as schizophrenia, diabetes, and cancer [23] (Fig. 1D). Notably, Smelik et al. analyzed multi-omics data from the UK Biobank and developed a proteome-based score to predict the incidence and mortality of common diseases [24]. This study underscores the advantages of proteomic data in terms of stability, making them more reliable as disease markers in clinical applications and providing new insights for predicting common diseases. Future research should focus on improving biomarker stability through enhanced sample processing and detection techniques, as well as the development of new evaluation methods. These efforts will contribute to advancing the accuracy and reliability of biomarkers in clinical applications.

#### Repeatability and individual differences

In biomarker research, reproducibility and individual variability are two key considerations. Repeatability ensures consistent and reliable results in biomarker detection, while individual differences reflect the variability of marker expression across different populations. Together, these factors contribute to the overall accuracy of biomarkers in guiding personalized treatment. Therefore, researchers and healthcare professionals must account for population heterogeneity when selecting and applying biomarkers [[25], [26], [27]] (Fig. 1B and E). Dou et al. emphasized the different proportions of the population characterized by biomarkers during the approval process of cancer treatments, both in accelerated and routine settings, highlighting the necessity of considering individual differences in precision medicine [28]. Moreover, understanding individual variability is critical for clinical trial design, as it helps identify patient groups most likely to benefit from specific treatments. The clinical application of blood-based biomarkers for AD by Teunissen et al. underscores the significance of individual differences in diagnosis and treatment. Studies have shown that plasma concentrations of phosphorylated tau at different sites—such as pTau181, pTau217, or pTau231—are significantly elevated in AD patients and correlate with gray matter atrophy [29]. This indicates that individual differences play a crucial role in AD diagnosis and treatment. To enhance the clinical utility of biomarkers, it is essential for studies to carefully consider repeatability and individual differences, ensuring rigor from design to analysis, and provide insights into patient diversity to accurately identify and validate markers that are instructive for disease management and treatment.

#### Non-invasiveness

The non-invasiveness of biomarkers refers to the principle that any harm to the patient should be minimized or avoided altogether when collecting these biomarkers. Such testing not only alleviates the physiological burden on patients but also reduces the risk of cross-infection during medical procedures, while leading to lower healthcare costs and resource consumption. In recent years, researchers have focused on discovering and validating biomarkers that can be obtained through non-invasive methods, such as blood, saliva, or urine samples, thereby eliminating the need for invasive surgeries or procedures. For example, Feinberg et al. studied the use of non-invasive fluid biomarkers in diagnosing mild traumatic brain injury (mTBI). The authors emphasize that these biomarkers provide a biological method for diagnosis and monitoring without the need for blood draws or neuroimaging examinations [31]. Additionally, in diagnosing acute kidney injury associated with cardiac surgery, traditional methods relying on changes in serum creatinine and urine output exhibit significant lag time. In contrast, new non-invasive biomarkers, such as neutrophil gelatinase-associated lipocalin and liver fatty acid-binding protein, can be quickly detected following the onset of renal injury, offering the potential for early diagnosis and intervention [32].

#### Practicability

The utility of biomarkers has grown increasingly important in the field of medicine, primarily reflected in their essential roles in disease diagnosis, treatment monitoring, prognosis evaluation, and research into disease mechanisms. To enhance the utility of biomarkers, standardization and normalized detection methods are crucial. Organizations such as the the American Association of Pharmaceutical Scientists have published standardized guidelines on biomarker research and reporting, emphasizing the significance of study design, sample handling, data analysis, and result reporting [33]. In particular, in the areas of cancer and CVD, the roles of biomarkers such as hormone receptors and Human Epidermal Growth Factor Receptor 2 (HER2) in breast cancer treatment are vital. These biomarkers not only guide treatment choice but also assist in assessing the risk of disease recurrence [30] (Fig. 1C). For example, the detection of BRCA1/2 gene mutations provides important prognostic information for patients with early HER2-negative breast cancer undergoing treatment with olaparib [30]. Additionally, the efficacy of immune checkpoint inhibitors (ICIs) in the treatment of hepatocellular carcinoma (HCC) could be enhanced by identifying newly developed predictive biomarkers. Such biomarkers allow for the accurate selection of patients most likely to benefit from ICI treatment [34].

### Classification of biomarkers

**Fig. 2:**
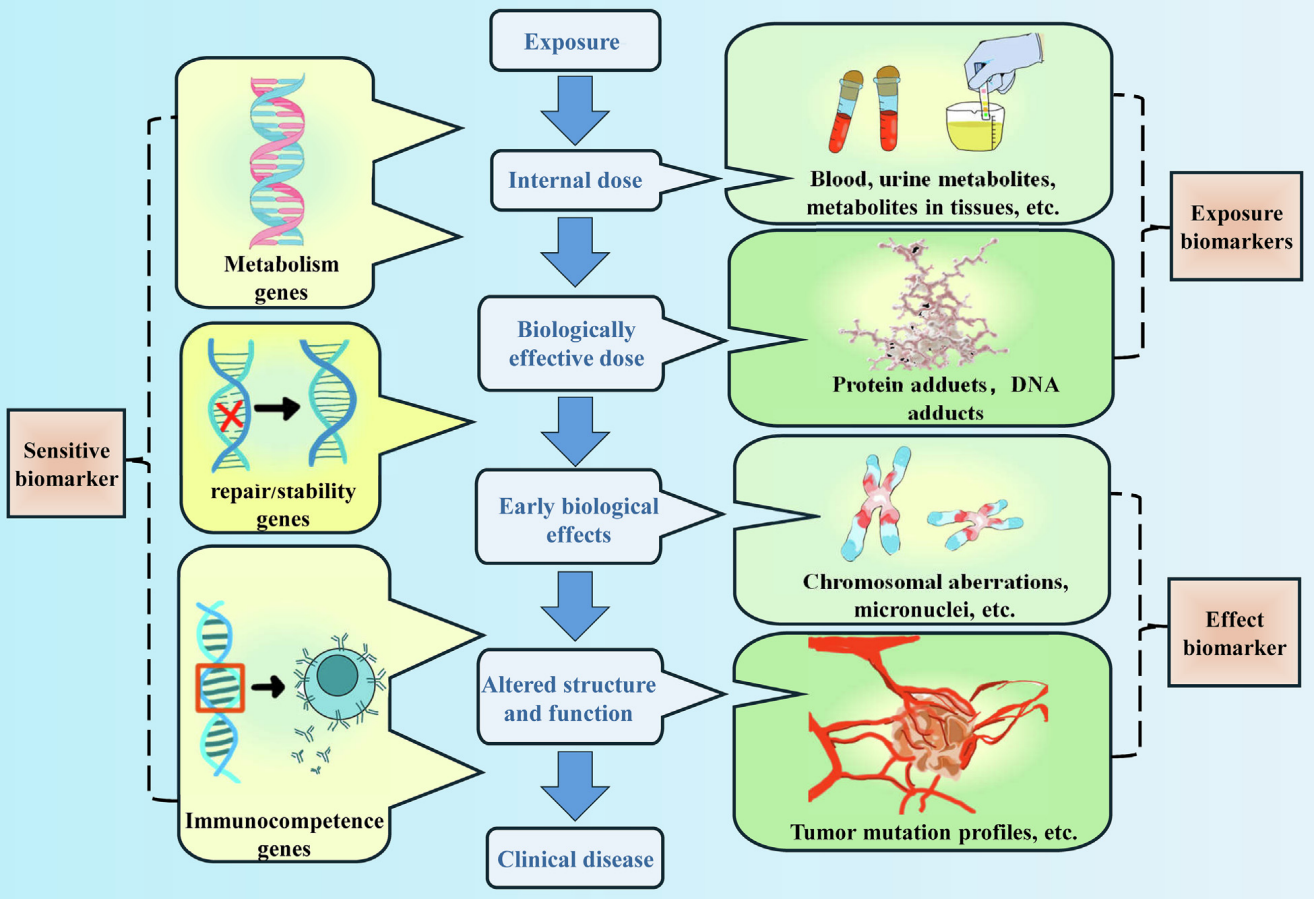
Schematic representation of the classification and dynamic association of biomarkers in the disease process.

Biomarkers are primarily classified into three categories based on their function: exposure biomarkers, effect biomarkers, and sensitivity biomarkers [35] (Fig. 2). Within these categories, exposure biomarkers can be further subdivided into in vivo dose biomarkers and effective dose biomarkers.

#### Exposure biomarkers

Exposure biomarkers refer to metrics that reflect the presence of exogenous chemicals and their metabolites, or the interaction products of endogenous substances within the body [36]. These biomarkers primarily include measurements of parent compounds, metabolites, or DNA and protein adducts. Exposure biomarkers can estimate the in vivo dose, biologically effective dose, or target dose [37], and they can be further classified into in vivo dose biomarkers and effective dose biomarkers. However, the focus and applications of in vivo dose biomarkers and effective dose biomarkers differ. In vivo dose biomarkers emphasize the level of chemical exposure, whereas effective dose biomarkers concentrate on the biological and health effects generated by these chemicals in vivo. It is important to note that while exposure biomarkers do not directly indicate the toxic effects of pollutants, they are essential tools for assessing individual exposure to environmental toxins and carcinogens.

##### In vivo dose biomarkers

In vivo dose biomarkers refer to biomarkers that can detect the exposure to exogenous chemicals and their metabolites. These biomarkers are crucial for understanding the absorption, distribution, metabolism, and excretion processes of foreign substances within the body, and they hold significant value for assessing individual health risks and disease management. Currently, in vivo dose biomarkers are primarily used for early diagnosis, treatment monitoring, and drug dose adjustment. Typically, the concentration of biological pollutants can be estimated by assessing the external environment. However, due to variations in the distribution, absorption, and excretion of toxins or pollutants among individuals, it is essential to measure these pollutants directly in individuals using in vivo dose biomarkers. This approach enhances the accuracy of assessing biological pollutant levels. For example, Scherer et al. evaluated the exposure of smokers to harmful chemicals in tobacco smoke using biomarkers, and assessed changes in exposure by comparing biomarker levels at different time points. Specifically, urinary metabolites such as 2-cyanoethylmercapturic acid (CEMA) and 4-aminobiphenyl (4-ABP) were utilized as in vivo dose biomarkers to evaluate the exposure of smokers to harmful substances in tobacco smoke [38]. Additionally, studies have indicated that biomarkers found in urine can reflect subtle changes in the body, aiding in the early detection of disease [[39], [40], [41]].

##### Effective dose biomarkers

Effective dose biomarkers refer to the amounts of pollutants that are absorbed, metabolically activated, and transported to a target site or an alternative site, along with the effective dose or concentration of reaction products from carcinogens that interact with the DNA or proteins of target cells. The primary focus of effective dose biomarkers is on the actual dose of pollutants or carcinogens at the target site or alternative site, and the extent of their interaction with biological components, as well as their crucial role in drug development and clinical applications [37].

For instance, Ji et al. employed the cell division blocking micronucleus test to assess chromosomal damage caused by vinyl chloride (VC). At designated time points during cell culture, cytotoxins such as cytochalasin B were introduced. This toxin inhibits cell division after DNA replication has occurred, while leaving nuclear division unaffected, resulting in cells with two nuclei. If chromosomal damage occurs during DNA replication, such as chromosome breaks or uneven segregation, the damaged chromosome fragments may not be retained in the two main nuclei but instead form smaller nuclei in the cytoplasm, known as micronuclei (MN). Research has shown that somatic characteristics and genetic susceptibility are linked to chromosome damage caused by VC, suggesting that the frequency of MN can serve as an effective dose biomarker for assessing VC exposure in vivo [42]. Consequently, effective dose biomarkers have significant application potential and research value in the fields of environmental health, clinical medicine, and drug research and development.

#### Effect biomarker

Effect biomarkers are measurable indicators produced by an organism in response to specific environmental exposures that lead to corresponding pathological changes. These biomarkers can include DNA and protein adducts, oxidative stress markers, genetic markers, and epigenetic markers. They play a crucial role in various fields, including clinical medicine, environmental health, and occupational health [43,44]. For instance, Robledo et al. investigated the effects of polybrominated diphenyl ethers (PBDEs) on liver toxicity in rats and mice. They utilized a range of biomarkers—such as liver weight, histopathological changes, hepatocyte steatosis, lipid accumulation, mitochondrial dysfunction, endoplasmic reticulum stress, and oxidative stress markers—as well as the apoptotic pathway to evaluate the extent of toxicity [45]. Similarly, Yu et al. employed doxorubicin (DOX) as a biomarker to predict cardiotoxicity in breast cancer patients. They found that changes in the levels of immunoreactive proteins prior to or during the early stages of DOX treatment correlated with the likelihood of developing cardiotoxicity, effectively allowing for the prediction of risk [46].

It is essential to note that the levels of some effect biomarkers may fluctuate over time and thus require measurement within specific timeframes. This temporal variability can present challenges for practical applications [47,48]. Moreover, effect biomarkers must reliably indicate the long-term harmful effects of pollutants on human health. Consequently, ongoing research and innovation are necessary to improve the persistence and reliability of such biomarkers in future studies.

#### Sensitive biomarker

Sensitive biomarkers refer to biochemical indicators that reveal an individual’s susceptibility to specific environmental factors, drugs, treatments, or diseases. These biomarkers can demonstrate stronger detectable reactions or an increased likelihood of adverse outcomes following exposure to certain stimuli [49]. Sensitive biomarkers are utilized to identify early, subtle biological changes that may arise before the onset of visible disease or pathology. This early detection is crucial for identifying corresponding conditions such as cancer, coronary artery disease, and others [50], [51], [52]. For instance, Liu et al. explored early sensitive epigenetic markers among a large cohort of workers exposed to low-dose benzene for the first time. Their research identified STAT3 hypomethylation as a sensitive biomarker reflecting early toxic effects, enabling the detection of adverse reactions to low levels of benzene exposure [53].

Additionally, sensitive biomarkers can be combined with other biomarkers to enhance disease treatment strategies. For example, Kurimoto et al. employed sensitive biomarkers—including anti-thyroglobulin antibody (TgAb), various cytokines (serum IL-1β, serum IL-2, and serum GM-CSF), and thyroglobulin (Tg)—to predict immune-related adverse events (IRES) related to thyroid function after treatment [54]. In this context, sensitive biomarkers are instrumental in identifying individuals who may experience adverse reactions to treatments, whereas effect biomarkers monitor and evaluate biological changes resulting from those treatments. It is important to note that the current research on sensitive biomarkers is still in its early stages, and further studies are needed with larger and more diverse populations to validate the applicability and generalizability of these biomarkers.

### Application of biomarkers

#### Application of biomarkers in disease diagnosis

**Fig. 3:**
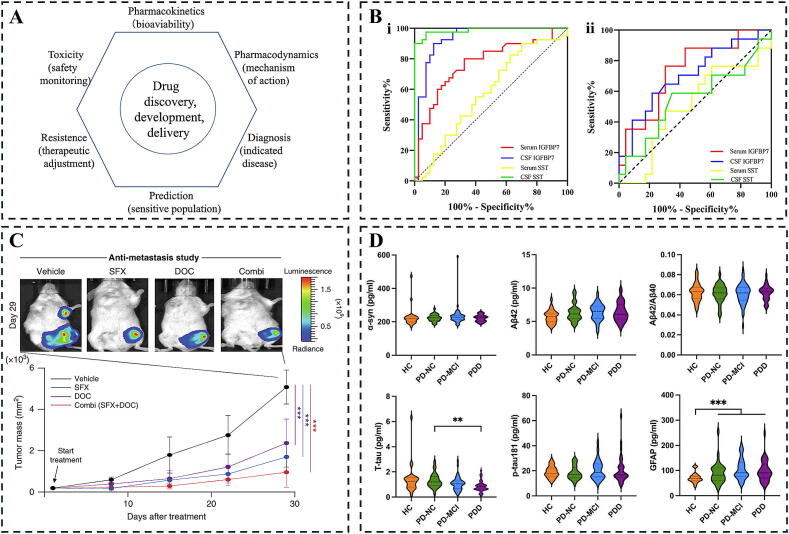
Metabolic pathway of brain natriuretic peptide (BNP) and application of biomarkers in disease diagnosis. (A) Overview of the application of biomarkers in drug development [65]. (B) Application of IGFBP7 and Somatostatin in the diagnosis of MS. (i) receiver operating characteristic (ROC) curve for MS diagnosis. (ii) ROC curve for distinguishing SPMS and RRMS [63]. (C) In vivo imaging and therapeutic effects in 4 T1 cancer cell-bearing mice. Top panel: In vivo images of 4 T1 cancer cell-bearing mice using the IVIS imaging system. Bottom Panel: Tumor mass volume of BALB/c nude female mice inoculated with 4 T1-luci (+) cells and treated with the specified drug or vehicle, with n = 9 per group [66]. (D) Comparison of plasma biomarker levels across different patient groups [67].

The application of biomarkers in disease diagnosis is a continually evolving field marked by ongoing development and innovation. Biomarkers, such as alkaline phosphatase, aspartate aminotransferase, and alanine aminotransferase, have long been utilized as critical indicators in the clinical diagnosis of liver disease [55,56]. Similarly, serum creatinine, urea, and uric acid are employed to diagnose kidney-related disorders [57,58], while aspartate aminotransferase, cardiac troponin, creatine kinase, and their isozymes serve as crucial markers for cardiac diseases [59]. Advances in molecular biology have facilitated the identification of numerous biomarkers, enhancing diagnostic accuracy and enabling early disease detection. For instance, Tran et al. employed high-throughput screening of urinary tumor DNA for the detection of bladder cancer, showcasing its efficacy in monitoring post-treatment progress and hinting at potential enhancements in early detection as well as treatment supervision for bladder cancer [60]. Maas et al. examined the noninvasive diagnosis and monitoring of bladder cancer, identifying DNA methylation as a promising avenue for biomarker development [61]. Furthermore, biomarkers can differentiate diseases with similar clinical manifestations, which typically reflects the distinct molecular mechanisms underlying diverse conditions. For example, Donadio et al. identified phosphorylated α-synuclein in the proximal peripheral nerve as a sensitive biomarker for diagnosing idiopathic parkinson’s disease, effectively distinguishing it from other Parkinsonian disorders [62]. Additionally, Shi et al. employed tandem mass tagging (TMT) quantitative proteomics, identifying insulin-like growth factor binding protein 7 (IGFBP7) as a potential biomarker for diagnosing and predicting multiple sclerosis (MS) progression [63] (Fig. 3B). This study introduces a new biomarker that could aid in differentiating MS from other neurological diseases while serving as a predictor of disease progression. Liu et al. conducted a systematic review and bibliometric analysis of potential biomarkers for mTBI [64]. Their findings indicated that molecular markers are the most studied category, accounting for 28.4 % of publications in this domain, and the volume of research has surged notably over the past five years, suggesting molecular markers may represent a key focus for future investigations. However, the concentration of molecular markers in biological samples can be exceedingly low, necessitating high-sensitivity detection techniques for accurate identification and quantification.

#### Application of biomarkers in drug research and development

Biomarkers represent essential tools in contemporary drug research and development, with their significance recognized throughout all stages of drug discovery. In the early phases of drug development, biomarkers assist in identifying drug targets and assessing the efficacy of candidate drugs. By analyzing the biological effects associated with these biomarkers, researchers can evaluate potential drug effects at an early stage [65] (Fig. 3A). In the clinical research phase, biomarkers serve as vital instruments that not only expedite the evaluation of a drug’s biological activity and safety but also optimize dosage selection and enable the precise stratification of patients most likely to benefit. This capability enhances the efficiency of clinical trials and allows for individualized treatment [68,69]. Furthermore, biomarkers facilitate the discovery of new uses or indications for approved drugs, thereby broadening their clinical applications. For example, Im et al. explored the potential repurposing of the antibacterial drug sulfisoxazole, which was identified as a specific inhibitor of breast cancer cell biogenesis and the secretion of small extracellular vesicles [66] (Fig. 3C). This study underscores the critical role of biomarkers in drug repurposing, particularly in identifying compounds that can effectively modulate specific biological pathways, and presents a precise and effective strategy for their application.

#### Application of biomarkers in prognosis evaluation

The application of biomarkers in prognostic evaluation is diverse, enabling insights into disease progression and outcomes. Precise measurement of biomarkers equips clinicians with powerful tools to refine prognostic stratification for patients. Notable examples include cancer antigen 125 (CA 125) for breast cancer [70], troponin and BNP for assessing prognosis in heart failure and myocardial infarction [71,72], β-amyloid and tau levels for evaluating AD prognosis [67] (Fig. 3D), and serum creatinine along with urine protein for assessing renal function and chronic kidney disease [73]. Through the quantitative analysis of these key biomarkers, physicians can identify patient subgroups with poorer prognoses, allowing for the development of targeted monitoring plans and intervention strategies that enhance overall clinical management [74,75].

Furthermore, for patients with chronic diseases, regular biomarker monitoring serves as a cornerstone of long-term health surveillance. These molecular indicators not only reflect microscopic changes in disease activity but also provide a precise quantitative means for evaluating the effectiveness of disease management. By continuously tracking biomarker levels, physicians can timely adjust treatment plans and optimize disease management strategies [76]. Additionally, patients can gain a clearer understanding of their health status through this data, allowing them to engage more actively in self-management. For instance, Chen et al. identified four key genes closely associated with the prognosis of ovarian cancer patients—COL6A3, CRISPLD2, FBN1, and SERPINF1 [77]. These genes hold promise as monitoring biomarkers for evaluating the survival of ovarian cancer patients and offer new molecular indicators for clinical treatment and prognostic assessment.

#### Application of biomarkers in response prediction

Efficacy prediction refers to the prediction of a patient's response to a specific treatment and therapeutic effect by analyzing the patient's biomarker, genome, transcriptome, proteome and metabolome data before or during treatment [78]. At present, immunotherapy efficacy prediction is one of the current research hotspots, especially in the field of oncology. The main class of immunotherapy is ICIs, which act by disinhibiting the immune system by tumor cells. The Tumor Microenvironment (TME) is composed of a variety of cell types, including cancer cells, immune cells, stromal cells, etc. The interaction between them has a significant impact on the response to immunotherapy [79]. Among them, the heterogeneity of TME means that even different regions within the same tumor may respond differently to treatment and can predict the response of patients to ICIs. For example, immune cell infiltration in TME, especially the presence of CD8 + T cells and regulatory T cells, can be used as a predictor of response to immunotherapy [80]. Immunosuppressive molecules in TME, such as PD-L1 and TGF-β, may be associated with resistance to immunotherapy, and their expression levels can provide clues to therapeutic response [81]. In addition, by integrating genomics, transcriptomics, proteomics and metabolomics data, the characteristics of TME can be comprehensively revealed and the accuracy of prediction models can be improved. Genomic and transcriptome analyses identify key immunotherapy response signatures, such as tumor mutational burden (TMB) and neoantigen expression, to inform personalized therapy. Ye et al. analyzed Immune characteristics to predict the response of patients to predicted Immune Checkpoint Blockade (ICB) [82]. ICB therapy enhances the immune system's attack on tumors by inhibiting immune checkpoints (such as PD-1/PD-L1). It is found that those patients who benefit from ICB treatment have increased their immune stimulation and inhibition characteristics after the start of treatment. This shows that the activity of immune cells in TME has changed, and these changes may be used as biomarkers to predict the curative effect. In addition, a new scoring system, immune activation score (ISΔ), was proposed based on the dynamic changes of plasma interleukin levels in patients. Among them, interleukins are a class of cytokines that play key roles in the immune response. If this finding IS validated in a broader patient population, the ISΔ score could become a useful clinical tool to help physicians make more informed treatment decisions and potentially improve patient outcomes. As an emerging therapy, effective biomarkers for the prediction and evaluation of immunotherapy efficacy are still under continuous exploration. Research results are being translated from the laboratory to clinical practice, and the predictive value of biomarkers is verified by clinical trials, which accelerates the development of predictive tools for immunotherapy efficacy. The study of immunotherapy efficacy prediction facilitates the targeted development of new drugs, and clinical trials can be designed more effectively by identifying patient populations that are likely to be susceptible to new drugs. However, despite the progress made in the prediction of immunotherapy response, the study of peripheral blood biomarkers still faces challenges, including the lack of large prospective studies, the need for detection standardization, and the integration of multiple biomarkers for evaluation.

### Biomarker summary

Biomarkers are indispensable tools in modern medicine, providing key insights for disease diagnosis, prognosis, drug development and efficacy prediction. Their utility depends on essential properties such as specificity, sensitivity, stability, reproducibility, non-invasiveness and utility. Classification based on function (exposure, effect, sensitivity) helps to understand their diverse roles in reflecting biological states and responses. Despite significant progress in identifying and applying biomarkers for various diseases, challenges remain in standardization, validation across different populations and disease stages, and discovery of highly specific and stable markers. Addressing these challenges through rigorous research and technological innovation is critical to advancing personalized medicine and improving clinical outcomes (Table 1).

**Table 1:** Biomarker Classification, Mechanisms, and Functional Characteristics.

Category	Subtype/Definition	Key Mechanisms	Functional Properties	Representative Examples & Applications	References
Exposure Biomarkers	In vivo dose biomarkers	Reflect the absorption, distribution and metabolism processes of exogenous chemicals in the body.	Quantify exposure levels Assess individual health risks	The CEMA and 4-ABP in the urine of smokers are used to assess the exposure to harmful substances in tobacco.	[38]
	Effective dose biomarkers	The biological effective dose of pollutants at the target site and their interactions with biological macromolecules.	Directly related to toxic effects Used in environmental health and drug development	MN frequency assessment of chromosome damage caused by VC exposure.	[42]
Effect Biomarkers	DNA/protein adducts, oxidative stress markers, etc.	Pathological changes in organisms in response to environmental exposures	Indicate early biological effects	In the PBDEs exposure study, liver tissue pathological markers were used to assess liver toxicity; DOX treatment was used to predict cardiac toxicity in breast cancer patients.	[45,46]
Susceptibility Biomarkers	Individual markers of sensitivity to environmental factors/drugs	Revealing the differences in responses caused by genetic or epigenetic variations	Early detection of microorganism changes Guidance for individualized treatment	Low methylation of STAT3 serves as an early toxicity marker for benzene exposure; TgAb combined with cytokines can predict adverse events of immunotherapy for thyroid diseases.	[53,54]

## Advances in biomarker imaging and detection

With the deepening of biomedical research, the imaging and detection technology of biomarkers has become a rapidly developing hotspot in the medical field. Biomarker imaging techniques, including traditional X-ray imaging [83], computed tomography (CT) [84], and ultrasonography (US) [85], as well as more advanced optical imaging [86], near infrared spectrum instrument (NIR) imaging [87], PET [88] and multimodal imaging [89], etc., have significantly improved our understanding of the spatial distribution and dynamic changes of biomarkers. These imaging techniques not only provide high-resolution images of biomarkers but also enable noninvasive detection at the in vivo level. These imaging techniques are not only able to provide high-resolution images of biomarkers, but also enable noninvasive detection at the in vivo level, which is essential for qualitative understanding of biological roles and pathological states of biomarkers. Secondly, the detection technology of biomarkers, such as immunodetection methods {Enzyme linked immunosorbent assay (ELISA), immunochromatographic analysis [90]}, molecular detection methods {Polymerase Chain Reaction (PCR), gene sequencing [91,92]}, protein detection methods {mass spectrometry, protein chip [93,94]} and detection methods based on material properties { MOF fluorescence detection platform [95]}, etc. provide powerful tools for accurate quantification and analysis of biomarkers. These techniques allow researchers to perform quantitative analysis of biomarkers, which play a key role in early diagnosis, efficacy evaluation, and prognosis judgment of the disease. At present, the development of new imaging probes and biosensors and the continuous progress of high-throughput technology have become the key ways to improve the accuracy and imaging effect of biomarker detection, which play an important role in promoting the accurate identification of biomarkers.

### The importance and implications of biomarker imaging and detection

Biomarker imaging and detection play a vital role in medical research and clinical diagnostics. They facilitate not only the early detection of diseases such as cancer but also the monitoring of disease progression, the assessment of treatment efficacy, and the implementation of personalized medicine. Biomarker imaging provides non-invasive information regarding disease status. For instance, optical imaging biomarkers have potential applications for early cancer detection and can enhance clinical workflows, particularly in evaluating skin lesions and monitoring patients with Barrett’s esophagus [86]. Detection methods for biomarkers, including fluorescent probe technology, offer highly sensitive and specific means for identifying biological substances, conducting cell imaging, tracking biochemical processes in vivo, and monitoring disease biomarkers [11]. Furthermore, with the recent approval of cell therapy products in clinical trials, cell therapies such as stem cell therapy and adoptive immunotherapy for tumors have garnered significant attention. Real-time imaging and observation of cells in vivo allow for visualizing cell distribution, tracking cell activity, and observing cell migration and growth [96]. Consequently, biomarker imaging and detection technologies are essential not only for foundational scientific research but also for their considerable potential in clinical practice. Their ongoing development and application will continue to advance the field of medicine toward increased precision and personalization.

### Key considerations in biomarker sample preparation

Biological sample pretreatment forms the critical foundation of biomarker sample preparation. For blood samples–which exhibit wide dynamic ranges–removal of high-abundance proteins (e.g., albumin) is essential to enhance detection sensitivity for low-abundance markers [97]. Tissue samples require standardized collection, storage (including anticoagulant selection, centrifugation parameters, and avoidance of freeze–thaw cycles), and homogenization protocols to address heterogeneity and ensure reproducibility [98]. Regarding injection technologies, microfluidic systems (pressure-, centrifugation-, magnetic-, or passive flow-driven) offer advantages through miniaturization, low consumption, and high-throughput capabilities (e.g., droplet technology), though sample compatibility remains a consideration [99].Mobile phase design necessitates precise microenvironment control. While microfluidics coupled with chromatographic techniques (e.g., online LC-MS/MS) can optimize separation efficiency and target enrichment, biocompatibility must be ensured to prevent protein denaturation. Interfacial effects significantly impact analytical performance: microscale solid–liquid interfaces exhibit size-dependent phenomena (e.g., reduced solute atomic concentration gradients leading to grain coarsening, as demonstrated in solder joint studies) [100]. Effective strategies to enhance stability include optimizing interfacial hydrophilicity through surface modifications (e.g., biofilms, hydrophilic coatings), reducing nonspecific adsorption, and careful selection of chip materials (paper-based substrates, etc.). In conclusion, synergistic optimization of sample processing standardization, microfluidic integration, mobile phase design, and interface modification is imperative to improve detection sensitivity and reproducibility in biomarker sample preparation [100,101].

### Technological innovations in biomarker imaging and detection

In the field of biomarker imaging and detection, the development of new imaging probes, the application of biosensors, and the application of high-throughput technology are three important innovation directions. Newly developed fluorescent probes, such as NIR region fluorescence enhanced probes, provide new wavelength options and higher imaging depths for multiplex fluorescence imaging in vivo [102]. As an interdisciplinary technology combining biology, chemistry, physics and electronics, biosensor is able to detect biomolecules or cells and convert them into quantifiable and tractable electrical or optical signals. High-throughput technologies, combined with automated and microquantitative experiments, accelerate the biomarker discovery and validation process and are of great significance for drug discovery and biomarker research.

#### Newly developed imaging probes

**Fig. 4:**
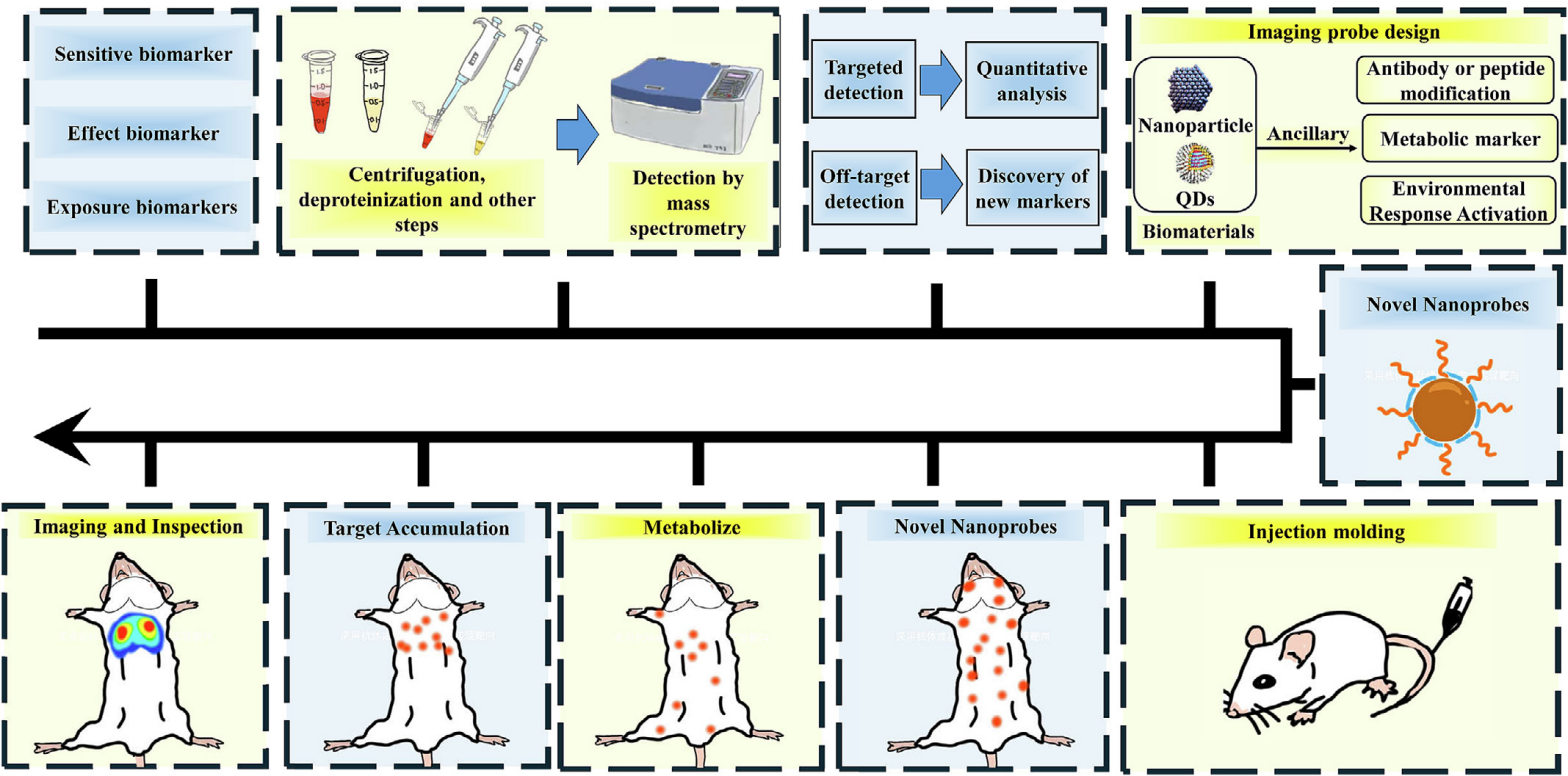
Schematic diagram of biomarker detection and imaging probe design and application.

Imaging probes are a class of molecules utilized in biomedical imaging that provide crucial information regarding biological processes, disease states, or treatment responses. These probes typically comprise a signal generator (such as a fluorophore or radioisotope), a targeting group (such as an antibody, peptide, or small molecule), and a linker that connects the two components. Imaging probes are specifically designed to interact with particular biomarkers or cellular processes, producing detectable signals that allow for the visualization of biological events (Fig. 4). Zhang et al. developed pcStar, a newly developed photoconverted fluorescence probe, and Quick-SIMBA, an innovative live-cell super-resolution imaging method [103]. The pcStar probe exhibits early luminescence and high light conversion efficiency, enhancing time resolution and labeling density in imaging, making it suitable for super-resolution imaging of short-lived biomolecules or structures. Quick-SIMBA technology offers high spatial and temporal resolution in single-molecule localization imaging of living cells, enabling clear analyses of the dense tubular endoplasmic reticulum structure within these cells. This advancement provides new insights and evidence for imaging ultra-early structures during development. Yang et al. introduced innovative rare-earth fluorescent probes with near-infrared (NIR) second windows, enabling real-time dynamic multiplex fluorescence imaging in vivo within the 1500 to 1700 nm range [102]. The development of these probes enhances high-resolution and high signal-to-noise ratio imaging in deep tissues, offering new tools for studying physiological mechanisms in situ. Wang et al. created a high-performance genetically encoded cyclic adenosine monophosphate (cAMP) green fluorescent probe, G-Flamp1, which demonstrates a fluorescence change of up to 12-fold in living cells [104]. This capability to monitor the spatiotemporal dynamics of cAMP signaling in specific neurons in real-time with high sensitivity provides crucial insights into the cAMP signaling pathway and its biological functions.

Notably, nanoprobes represent a focal point of current research, particularly in the fields of biomedical imaging, diagnosis, and treatment, demonstrating significant potential and application value. Nanoprobes typically refer to particles with nanoscale dimensions that are deliberately designed to facilitate the identification and imaging of specific targets within living organisms. These probes may be composed of various materials such as Magnetic Nanoparticles (MNPs), QDs, or carbon nanotubes, and can be modified to carry targeting ligands, drugs, or imaging tags. Moreover, nanoprobes integrate multiple imaging techniques, including magnetic resonance imaging (MRI), PET, CT, and fluorescence imaging, to achieve high-resolution imaging of tumors and other lesions [105,106]. However, despite their promise demonstrated in laboratory studies, the clinical translation of nanoprobes continues to encounter challenges. Researchers are diligently working to overcome these obstacles, aiming to translate these probes into clinically practical tools.

#### Biosensors

A biosensor is an instrument designed to detect biological substances by converting their concentration into an electrical signal for analysis. This analytical tool or system consists of an immobilized biosensitive material that serves as the recognition element, along with a suitable physicochemical transducer and signal amplification device. Biosensors can be classified according to their detection principles into various categories, including thermal biosensors, field effect tube biosensors, piezoelectric biosensors, optical biosensors, acoustic wave channel biosensors, enzyme electrode biosensors, and mesomeric biosensors. Among these, electrochemical biosensors are a significant subset, valued for their ability to detect biomarkers in body fluids by transforming biorecognition events into electrical signals. Typically, this process involves redox reactions occurring on the electrodes’ surface, which produce changes in current or voltage, facilitating the quantitative analysis of target molecules [107,108].

Nevertheless, integrating these sensors into portable devices for point-of-care detection presents technical challenges, particularly in enhancing sensing accuracy, enabling multiplexing, and achieving one-step processing [109]. To address these issues and improve analysis accuracy and reliability, researchers have developed photoelectrochemical (PEC)-coupled dual-mode biosensors. These biosensors merge the high sensitivity and operational ease of PEC analysis with ratiometric detection and dual-mode strategies to minimize external interferences. For instance, Xu et al. developed an enhanced PEC biosensor based on portable piezoelectric and plasma exciton effects for the instantaneous detection of low-abundance cancer markers [110]. This sensor utilized localized Surface plasmon resonance (SPR)-enhanced and piezoelectric effects between silver nanoparticles and the piezoelectric material NaNbO3 to achieve sensitive detection of prostate-specific antigen with an ultra-wide linear range and ultra-low detection limit when excited by portable UV light.

Particularly, researchers are investigating advanced materials to enhance the performance of electrochemical, optical, piezoelectric, and mass spectrometric sensors. The unique structures and tunable physicochemical properties of these materials make them promising for the development of a variety of sensing systems [111]. Future technological advancements and innovations should focus on promoting the integration and application of cutting-edge technologies, including nanotechnology, biomaterials, and microfluidics, to improve the sensitivity, stability, and reliability of sensors.

#### High-throughput technology and machine learning, artificial intelligence, computational chemistry

High-throughput technology, as a cutting-edge scientific tool, demonstrates significant potential across diverse research and industrial fields through its capacity for efficient large-scale sample processing and analysis. Indispensable from chemistry to materials science, its core strength lies in rapid data collection and analysis, substantially enhancing research efficiency and accuracy. In biology, key applications encompass sequencing, proteomics, cell imaging, flow cytometry, and microarray technologies. Integrated high-throughput screening combines automation with microquantitative experimentation to rapidly evaluate candidate biomarkers, simultaneously improving screening efficiency while reducing costs and accelerating identification of promising targets. Within biomarker imaging and detection, this technology drives development of newly developed imaging probes and biosensors while optimizing detection methods. For example, Ministro et al. developed a CRISPR/Cas9-based platform using mammalian endoplasmic reticulum synthetic libraries for high-throughput antibody discovery against natural antigens, significantly accelerating therapeutic molecule identification[112]. Despite advances, challenges persist in enhancing imaging resolution/sensitivity, reducing costs/time, and integrating multi-platform systems. Future progress will focus on combining newly developed probes and biosensors to improve imaging clarity/accuracy while leveraging automation, refined data analysis, and AI-driven intelligence for efficient processing. Cross-disciplinary innovation will further expand applications and maximize the technology's unique advantages.

Machine Learning (ML), Artificial Intelligence (AI), and Computational Chemistry critically advance the development of biomaterials for biomarker imaging and detection. Through deep integration with high-throughput technologies, they accelerate the discovery, optimization, and application of biomaterials. For example, ML models predict sensing performance (e.g., sensitivity, selectivity) of novel nanomaterials—including carbon-based materials and silicon nanowires—and guide compositional optimization of biomaterials like hydrogels and high-entropy alloys by learning complex composition-structure–property relationships [113]. Computational chemistry provides essential theoretical guidance for designing high-affinity biosensing materials, using molecular simulations and quantum mechanical calculations (e.g., density-functional theory) to predict electronic structures, surface properties, and biomolecular interactions [114]. Notably, recent studies demonstrate that ML-driven signal processing combined with nanomaterial biosensors (e.g., self-powered PEC devices) enables real-time monitoring of ultra-low-concentration biomarkers in human body fluids, establishing a new paradigm for precision medicine [115]. However, challenges persist in standardizing biomaterial data, overcoming small-sample learning limitations, and accurately simulating material-biological interface interactions in complex biological environments [116]. Future progress will require fusing computational chemistry's physical models with data-driven ML methods to develop intelligent, personalized biosensing platforms.

### Summary of imaging and detection advances

The field of biomarker imaging and detection is rapidly evolving, thanks to continued technological innovation. Novel imaging probes [e.g., pcStar, NIR-II probes, Aggregate-induced Emission Luminogen (AIEgens)] and biosensors (e.g., PEC-coupled dual-mode sensors) have achieved unprecedented sensitivity, specificity, resolution, and multiplexing capabilities. High-throughput technologies have significantly accelerated biomarker discovery and validation. These advances are critical for enabling non-invasive, real-time visualization and quantitative analysis of biomarkers in vivo, contributing to a deeper understanding of disease mechanisms and progression. However, challenges remain in translating laboratory innovations into robust, standardized, and cost-effective clinical tools, particularly in terms of integration, portability for field care, and long-term stability. Overcoming these barriers is critical to realizing the full potential of these technologies in routine clinical practice.

## Biomaterials for biomarker imaging and detection and their applications

Biomaterials not only provide biocompatibility and reduce immune response and nonspecific adsorption in biological samples, but also enhance signaling and improve the sensitivity and selectivity of sensors. In particular, nanomaterials such as carbon nanotubes and gold nanoparticles significantly enhance detection performance due to their high surface area and special electrochemical properties. In addition, the multifunctionality of biomaterials enables biosensors to integrate biocomputing and storage functions, and the development of newly developed biomaterials has further advanced sensor technology. These materials also contribute to the fabrication of low-cost, disposable, portable biosensors for rapid on-site detection and resource-limited environments, showing great potential for applications in areas such as medical diagnostics.

### Biomaterials for biomarker imaging

The central role of biomaterials in biomarker imaging is to function as imaging probes or contrast agents tailored to specific modalities. These materials mediate signal changes or contrast enhancement at target sites through biomarker interactions (e.g., specific binding, environmental response) or via intrinsic physicochemical properties (e.g., magnetism, radioactivity, fluorescence). These detectable signals, captured by external imaging devices, thereby produce visual representations of biomarker spatial distribution, abundance, and dynamic processes–enabling precise localization, real-time tracking, and comprehensive visual analysis.

#### Fluorescent materials

Fluorescent materials possess the capability to image specific biomarkers with high sensitivity and specificity, both in organisms and in vitro, due to their unique fluorescent properties. This imaging technology facilitates not only the detection and identification of biomarkers but also the elucidation of their distribution, dynamic changes, and interactions with other biomolecules within the organism. Both organic small-molecule fluorescent probes and fluorescent nanoparticles (FNMs) are utilized for biomarker imaging; however, they differ in terms of size, structure, and synthesis methods. Organic small-molecule fluorescent probes emphasize molecular-level design and functionality, whereas FNMs focus on nanoscale engineering and applications. Aggregation-induced emission (AIE) materials are differentiated from other fluorescent materials by their ability to emit light weakly in dilute solutions, with a significant increase in intensity occurring upon aggregation or in the solid state. This high luminescence efficiency exhibited in the aggregated state provides AIE materials with a distinct advantage for use as bioimaging probes and in cancer therapies.

##### Fluorescent molecules

**Fig. 5:**
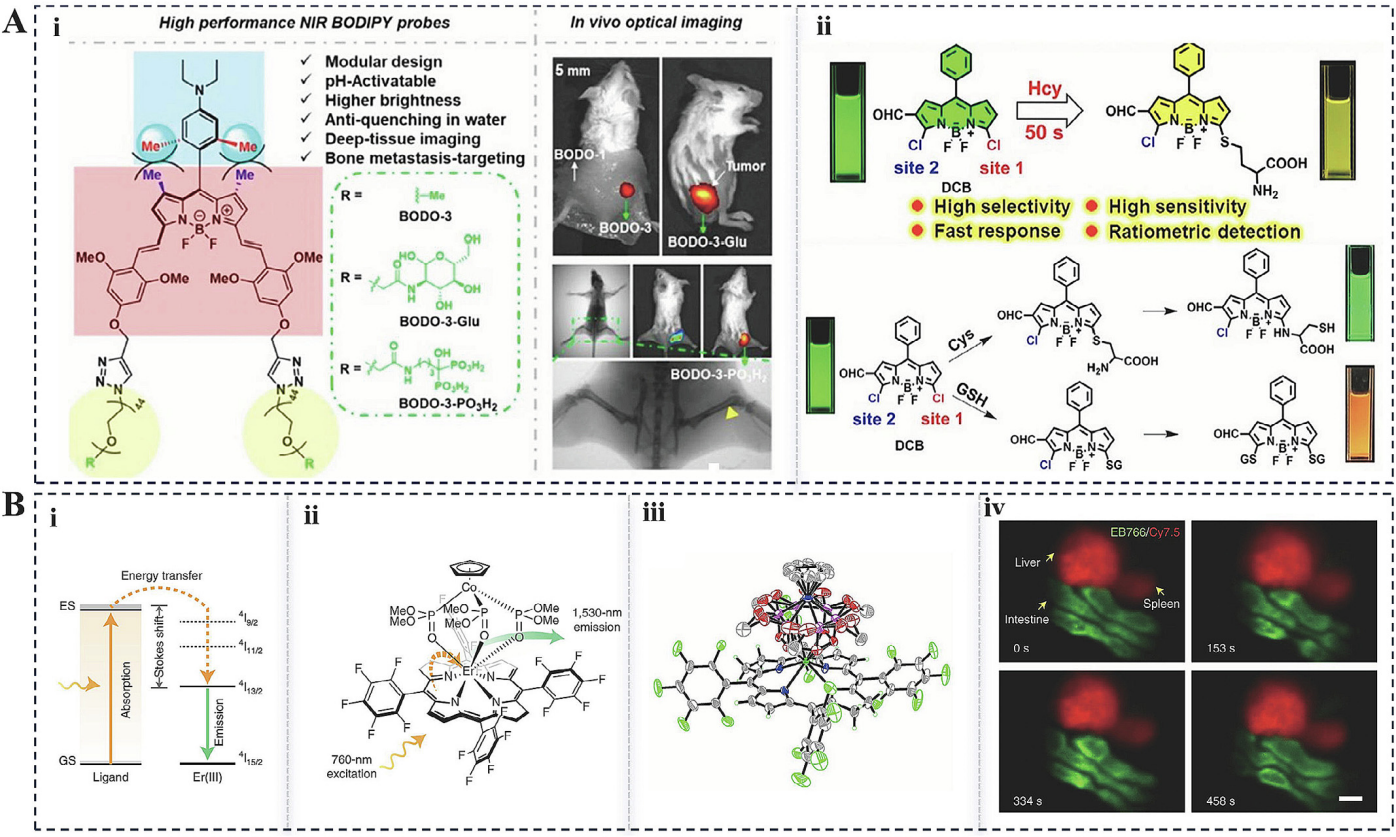
Integrated demonstration of fluorescent molecular structures and bioimaging techniques. (A) Molecular structure and reaction mechanism: (i) (ii)The schematic illustrates the modular structure of pH-responsive NIR BODIPYs. (iii) This section describes the mechanism by which DCB interacts with homocysteine (Hcy), cysteine (Cys), and glutathione (GSH) [119]. (B) Hair light sensitization and bioimaging: (i) The sketch depicts the photosensitization process of Er (III) complexes, which includes ligand absorption, energy transfer, and metal center emission. Here, “gs” denotes the ground state, while “es” indicates the excited state. (ii) The chemical structure of EB766 is presented. (iii) An X-ray crystal structure of EB766 is depicted, with ellipsoids shown at a 30% probability level and color-coded as follows: Er (dark green), N (blue), F (green), C (gray), O (red), and P (pink). (iv) Superimposed images of Cy7.5 from the liver and spleen are shown alongside EB766 images of the gastrointestinal tract; these representative images were replicated across three biologically independent mice [120]. (For interpretation of the references to color in this figure legend, the reader is referred to the web version of this article.)

In biomedical research, the development and application of fluorescent molecules as key tools for bioimaging and biomarker detection, mainly includes fluorescent protein (FP), fluorescein, rhodamine, acridine orange, and other fluorescent probes, and has been a hot spot in scientific research. Through organic synthesis methods, such as condensation and substitution reactions, groups with fluorescent properties are attached to the molecular structure, thus forming fluorescent molecules [117]. In recent years, researchers have made remarkable progress in designing newly developed fluorescent probes, improving imaging resolution, and developing semi-synthetic fluorescent biosensors. Among them, organic small-molecule fluorescent probes have been widely studied due to their easy synthesis and structural tunability. For example, Wang et al. developed NIR-II fluorophores based on asymmetric small organic molecules for high-performance tumor photothermal and photodynamic therapy [118]. These fluorophores exhibited 90.8 % tumor inhibition under 808 nm laser irradiation, thanks to their excellent NIR-II fluorescence imaging and photothermal/photodynamic properties. In addition, fluorophores such as boron-dipyrromethene, rhodamine, and tetraphenylethylene showed unique advantages in cancer biomarker detection [119] (Fig. 5A). To achieve high sensitivity and super-resolution imaging, Wang et al. achieved high-contrast in vivo fluorescence multiplexed imaging in the NIR region through chemical modifications, e.g., by utilizing the rare-earth element erbium (Er^3+^) for coordination with bacterial chlorophyll [120]. Such probes not only exhibit bright fluorescence emission in the aqueous phase, but also exhibit narrow half-peak widths and large Stokes shifts in their absorption and emission spectra, which provide a powerful tool for in vivo fluorescence multiplex imaging (Fig. 5B). In addition, to overcome the limitations of FP-based biosensors for live cell imaging, researchers have developed semi-synthetic fluorescent biosensors. These sensors use chemically modified fluorescent probes and self-labeled protein tags or DNA/RNA-based hybridization systems with the unique advantage of easy modification and self-labeled protein tags to label the synthetic probes through covalent bonds [121]. For example, Xue et al. constructed a semi-synthetic biosensor for accurate detection of CoA concentration and distribution in living cells [122]. This sensor consists of a complex of self-tagged proteins, FP, and CoA receptor proteins, and is capable of quantitative detection of CoA through fluorescent color changes. This study reveals the mechanism of CoA homeostasis and metabolic regulation within subcellular compartments and provides a molecular tool for the development of inhibitors or drugs for CoA metabolism-related diseases.

##### Fluorescent nanoparticles

**Fig. 6:**
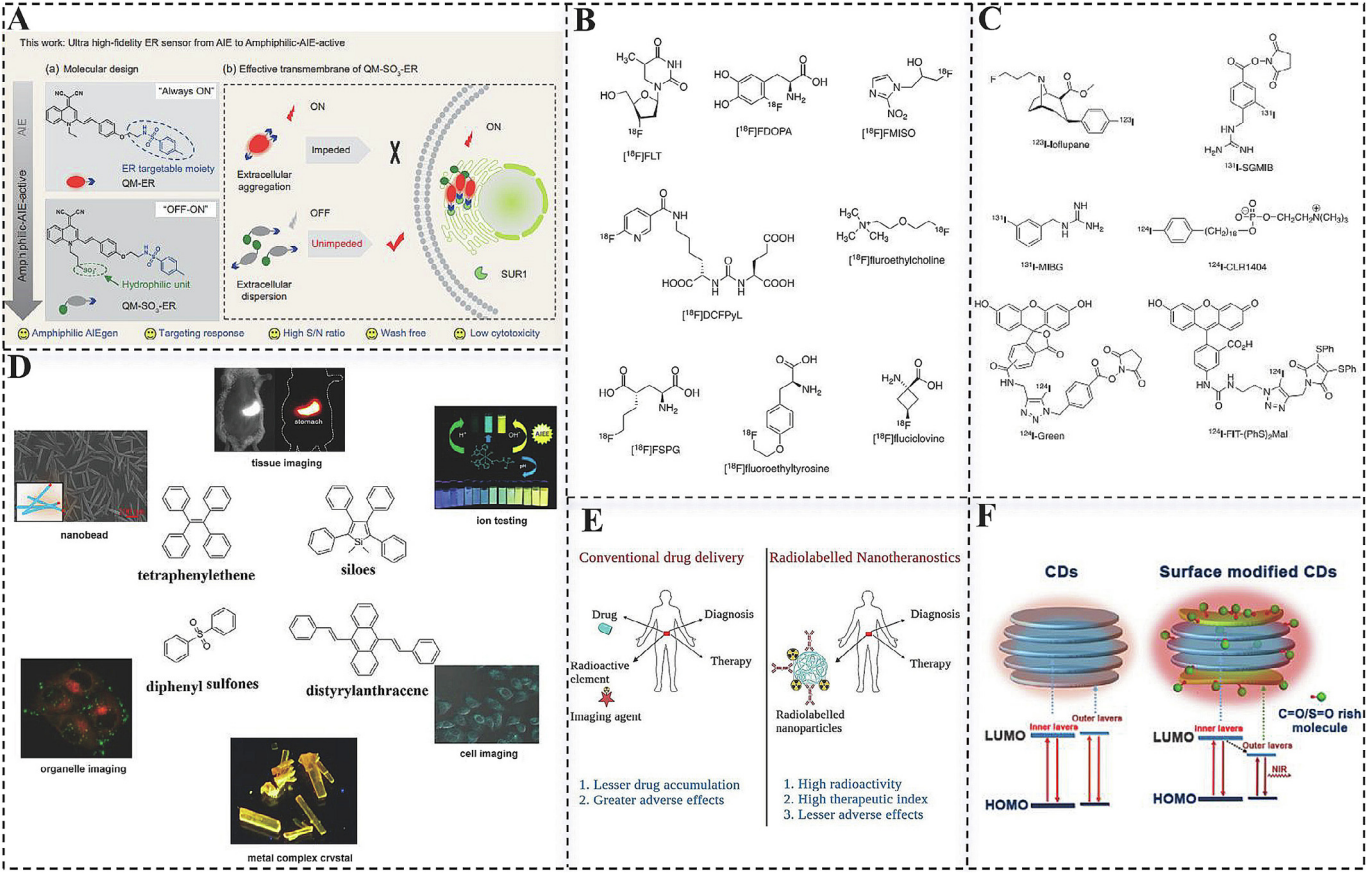
Innovative applications of AIE materials coupled with radioisotope labeling techniques. (A) AIE-Active Sensors in Endoplasmic Reticulum (ER) Tracking:(a) The molecular structures of QM-ER and QM-SO3-ER illustrate how sulfonate groups are incorporated into QM-ER to enhance ER targeting. (b) The amphiphilic QM-SO_3_-ER demonstrates good solubility and cellular uptake in aqueous solutions, achieving “off–on” signaling to the ER through the KATP-targeting motif and the RIM mechanism of AIE [137]. (B) Examples of 18F-labeled small molecule visualizers illustrate the physicochemical properties of the PET nuclide 18F and its applications [140]. (C) Radioiodine I-labeled nuclear drugs and biocoupling reagents [142]. (D) Development of common AIE backbone structures [134]. (E) Applications of radioisotope-labeled materials in therapeutic diagnostics include advancements in radionuclide labeling methods involving various nanocarriers [138]. (F) NIR-Emitting CDs in bioimaging [124].

FNMs play a crucial role in bioimaging and fluorescence-dependent detection due to their distinctive optical properties. Researchers have been actively exploring newly developed FNMs with enhanced attributes to improve selectivity and photoluminescence efficiency for both imaging and detection [123]. The synthesis of fluorescent nanoparticles is usually carried out using methods such as chemical reduction and sol–gel methods. Among these, Carbon Dots (CDs) and Upconversion Nanoparticles (UCNPs) represent two promising classes of fluorescent nanomaterials with significant applications in bioimaging. Each type presents unique advantages and contributes substantially to imaging techniques. CDs are a specific class of fluorescent carbon nanomaterials characterized by distinctive structures that have demonstrated promising applications in bioimaging, biosensing, and therapeutics. The optical characteristics of CDs present new opportunities for deep tissue fluorescence imaging, particularly in terms of absorption and emission in the near-infrared region, thereby providing in situ photothermal effects for photoacoustic imaging (PIA) and photothermal therapy [124] (Fig. 6F). Furthermore, UCNPs are capable of emitting high-energy photons within the visible wavelength range under near-infrared light excitation, exhibiting enhanced tissue penetration depth and biocompatibility [125]. Erbium (Er)-doped upconversion nanoparticles have been utilized as luminescent centers in bioimaging owing to their strong absorption and emission of red and green light in the near-infrared II region (1500 nm) [126]. For instance, Lan et al. synthesized CDs via a hydrothermal method using 1,3,6-trinitropyrene and Na_2_SO_3_, resulting in dots that exhibited excellent photothermal conversion efficiency under 800 nm femtosecond pulsed laser irradiation [127]. Additionally, these dots were capable of generating intense fluorescence through two-photon excitation and producing singlet oxygen, thus demonstrating potential as multimodal therapeutic agents in photoacoustic and fluorescence imaging, as well as in photothermal and photodynamic synergistic cancer therapy. Yang et al. achieved upconversion of luminescence color by doping Yb^3+^/Ho^3+^ ions into a NaYF_4_ matrix and co-doping Ce^3+^ ions for modulation and enhancement of luminescence intensity [128]. This strategy facilitates energy transfer between Ho^3+^ and Ce^3+^ ions through the cross-relaxation (CR) process, resulting in modulation of luminescence color and a substantial increase in the red-to-green (R/G) luminescence ratio.

Persistent Luminescence Nanoparticles (PLNPs), a type of fluorescent nanomaterial, exhibit unique optical properties [129]. These materials can absorb and store excitation light energy, enabling them to emit light for an extended period following the cessation of the excitation light. By not relying on real-time excitation light, they effectively minimize spontaneous fluorescence interference, making them suitable as sources of fluorescence deep within biological tissues, thus facilitating the activation of biochemical reactions. This characteristic highlights the wide range of potential applications for PLNPs in biomedical detection, bioimaging, and tumor therapy. Jinnai et al. demonstrated that highly efficient organic long afterglow luminescence can be achieved in a p-type system utilizing an organic photoredox catalyst dopant under visible light excitation [130]. Furthermore, the luminescence properties can be optimized through careful adjustment of the type and concentration of the dopant, providing innovative avenues for the development of new long afterglow luminescent materials.

##### Aggregation-induced emission materials

AIE is a distinctive photophysical phenomenon in which certain molecules exhibit weak or negligible luminescence in dilute solutions, yet their luminescence is significantly enhanced in the aggregated or solid state (Fig. 6D). Their synthesis methods include polycondensation reaction [131], stepwise growth method [132] and so on. Luo et al. were the first to report hexaphenylsilane as the pioneering AIEgen, thereby establishing a new field of innovative scientific research [133]. The primary advantage of AIEgens is their high luminescence efficiency in the aggregated state [134], which contrasts with traditional aggregation-caused quenching materials. With low background signals, high photostability, and resistance to photobleaching, AIEgens can provide reliable signals for bioassays and enable long-term tracking and monitoring capabilities. Consequently, AIEgens have been utilized to develop newly developed biomarker-activated probes that emit fluorescent signals only upon interaction with specific biomarkers. This property enhances the contrast and accuracy of imaging, particularly in fluorescence and PIA for detecting disease biomarkers [135]. Moreover, AIEgens can enhance therapeutic efficiency through targeted drug delivery, image-guided laser ablation, and image-guided surgery, garnering significant attention in cancer treatment. For example, Chen et al. proposed a newly developed method for preparing NIR long afterglow nanoprobes based on AIEgens, which can simultaneously red-shift and enhance afterglow signals for advanced tumor imaging and image-guided tumor resection [136]. However, AIEgens face challenges in bioimaging applications, especially regarding biocompatibility. To enhance the applicability of AIEgens in biological systems, molecular design modifications are essential to improve their water solubility, extend their circulation time within organisms, and reduce their clearance. Therefore, Zhu et al. proposed an “amphiphilic AIE” design strategy, successfully synthesizing an amphiphilic AIE molecule, QM-SO_3_-ER, by introducing a hydrophilic sulfonic acid group and a sulfonamide group containing a heteroatom onto the parent quinolinonitrile (QM) structure [137] (Fig. 6A). This design enables the AIE probe to achieve effective dispersion in both aqueous and lipid environments, effectively minimizing nonspecific aggregation and false-positive signals while improving specificity and the signal-to-noise ratio of imaging. This strategy offers new perspectives and directions for the design of AIE bioprobes.

#### Radioisotope-labeled materials

Radioisotopes are isotopes characterized by unstable nuclei that decay by spontaneously emitting radiation, including α-particles, β-particles, and γ-rays, thereby transitioning to a more stable state [138]. They are usually prepared through the chemical combination of radioactive isotopes with biological molecules. Radioisotope therapy (RIT) represents a cancer treatment strategy that leverages ionized atoms and free radicals generated by radioisotopes to disrupt DNA single strands and induce apoptosis in cancer cells (Fig. 6E). The primary types of nuclides used in RIT include α-particle emitters, β-particle emitters, and Auger particle emitters. To enhance bioavailability and minimize physiological toxicity, researchers have explored various biomaterials as multifunctional nanocarriers. These materials encompass targeting molecules, macromolecular monoclonal antibodies, peptides, inorganic nanomaterials, as well as organic and polymeric nanomaterials, facilitating the precise delivery of therapeutic radioisotopes [139,140] (Fig. 6B). Liang et al. proposed employing nanoparticle-mediated internal RIT to locally augment the permeability of tumor vasculature, thereby enhancing the tumor-specific uptake of second-wave therapeutic nanoparticles and improving overall treatment efficacy [141].

Additionally, the radiation emitted by radioisotopes can be detected using nuclear medicine imaging techniques, such as single-photon emission computed tomography (SPECT) and PET, which facilitate the tracking and imaging of biomolecules in vivo. Among these techniques, radioactive iodine labeling technology has emerged as a significant focus in the field of nuclear medicine, particularly for the development of radiopharmaceuticals that target specific molecules. Commonly used radioiodine isotopes include iodine-125 (^125^I) and iodine-131 (^131^I), both of which emit detectable radiation through γ-rays or β-particles [142] (Fig. 6C). In this context, biological materials, particularly proteins and peptides, serve as key targets for radioiodine labeling. The direct labeling method is frequently employed in radiopharmaceutical development due to its operational simplicity, rapid response, and high radiochemical yield. This method utilizes potent oxidants, such as chloramine T or Iodogen, to efficiently convert iodine anions into iodine molecules, which then bind firmly to tyrosine residues in biological materials for effective radiolabeling of target molecules [143,144]. However, despite the notable efficiency of the direct labeling method, the labeled molecules may be susceptible to insufficient stability and deiodination in vivo, which somewhat limits their applicability. By contrast, the indirect labeling method significantly enhances the stability of labeled molecules in vivo, although it involves more complex procedures such as pre-binding to auxiliary moieties followed by radiolabeling. This strategy effectively mitigates the in vivo instability issues associated with direct labeling by utilizing a carefully designed chemical pathway. However, the indirect labeling method may also lead to decreased radiochemical yield and potentially alter the original active structure of the biomaterial, necessitating careful trade-offs in the selection and optimization of the labeling strategy. Chen et al. developed radioiodine isotope-labeled ultrathin Pd nanosheets, successfully employing them as a pH-sensitive diagnostic platform in cancer diagnosis and therapeutic research, thereby providing a new direction for future radiochemical studies and in vivo biological applications of nanomaterials [145].

#### Magnetic nanomaterials

**Fig. 7:**
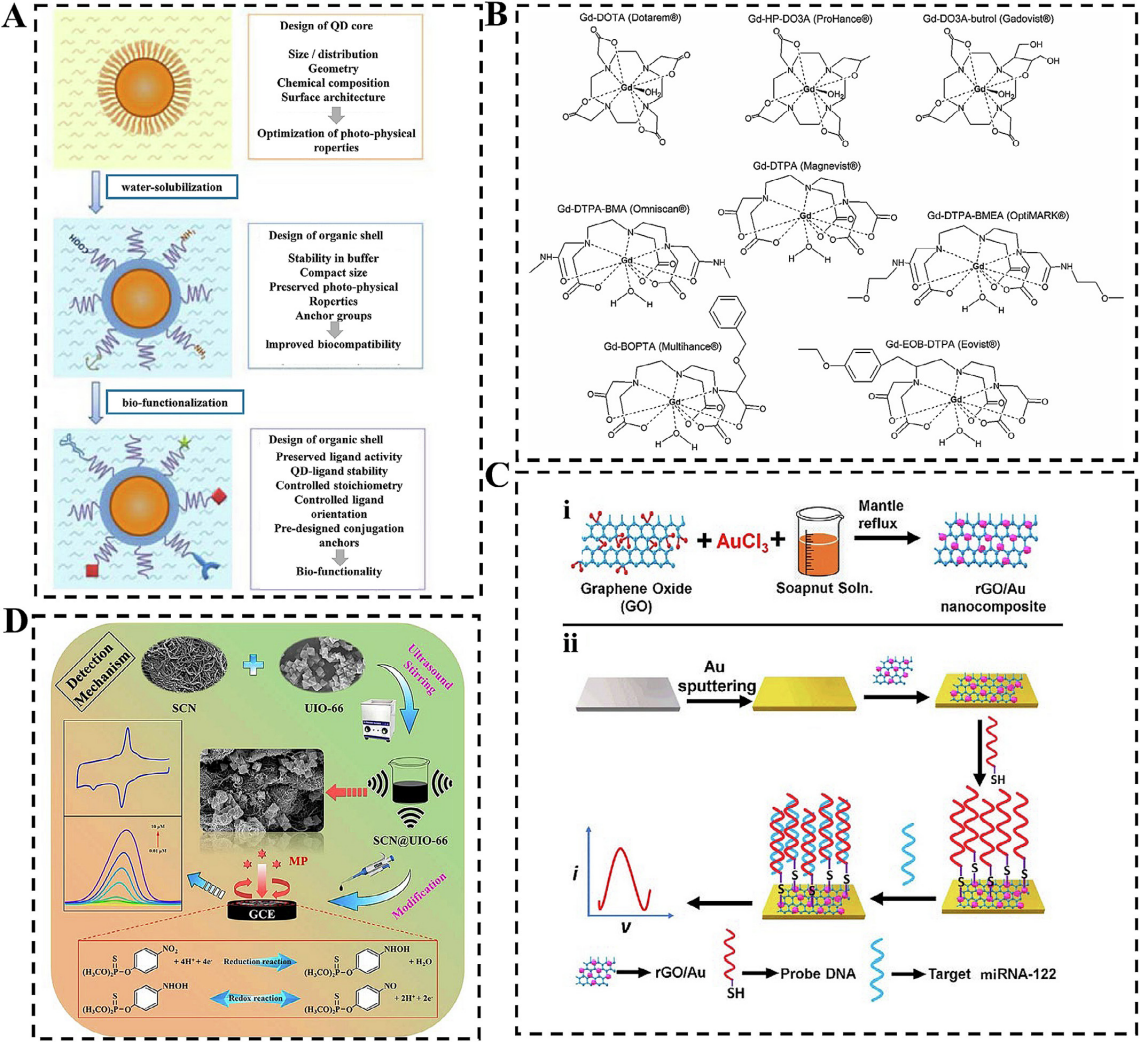
Nanomaterials in biomedical detection and imaging. (A) General steps and design guidelines for engineering QD probes for biomedical use [176]. (B) Commercially available Gd^3+^-chelated MRI contrast agents for MRI [148]. (C) rGO/Au nanocomposites and their electrochemical detection platform. (i) Synthesis process of rGO/Au nanocomposites. (ii) Schematic representation of the fabrication of the miRNA-122 electrochemical detection platform based on rGO/Au nanocomposites [158]. (D) Carbon nanotube-based electrochemical sensing platform for the fabrication and implementation of ion detection [170].

MNPs in biomarker imaging represents a significant branch in medical imaging, encompassing MNPs and other magnetic nanostructured materials. These biomaterials exhibit great potential in biomolecular imaging due to their unique physicochemical properties, including superparamagnetism and biocompatibility [146]. MNPs enhance image contrast by altering the relaxation time of water molecules, thereby allowing for clearer visualization of lesion areas [147]. Typically, these nanoparticles are designed to be superparamagnetic, meaning they display magnetic properties in the presence of an external magnetic field but lose this magnetism once the field is removed. Additionally, owing to their physicochemical properties and favorable biocompatibility, rare earth-doped paramagnetic nanoparticles are widely used as contrast agents in MRI (Fig. 7B). These nanomaterials generally possess a specific iron oxide core coated with an outer layer of polymeric materials such as dextran, carboxymethyl dextran, chitosan, starch, heparin, albumin, and polystyrene, which effectively reduce signal intensity by shortening the transverse relaxation time (T2) of H_2_O [148]. A series of gadolinium (Gd)-based classical complexes, including Gd-DTPA, Gd-DTPA-BMA, Gd-EOB-DTPA, Gd-MS-325-L, Gd-DTPA-BMEA, Gd-BOPTA, Gd-DO3A-butrol, Gd-DOTA, and Gd-HP-DO3A, have established themselves as MRI contrast agents [149]. These complexes are efficient in reducing the T1 relaxation time of tissues in MRI images due to their excellent paramagnetism, thereby enhancing image contrast; most existing Gd (III) complex contrast agents have been modified and optimized based on these foundational complexes. Notably, the design and synthesis of MNPs are crucial for their effectiveness in biomarker imaging applications. Future exploration of different synthesis methods and surface modification techniques is necessary to improve the performance of MNPs, focusing on enhancing their stability, targeting, and biocompatibility in organisms to play a vital role in the early diagnosis of diseases and therapeutic monitoring.

#### Multimodal imaging materials

Multimodal imaging materials are nanomaterials that can respond to multiple imaging modalities, and they hold significant promise in medical diagnosis and treatment. These materials can simultaneously provide anatomical, functional, molecular, and metabolic information, contributing to a comprehensive understanding of disease states and the monitoring of treatment effects. Among multimodal imaging techniques, common imaging modalities include PET, MRI, CT, optical imaging (e.g., fluorescence and bioluminescence), and US. By integrating these imaging techniques, richer information, encompassing structural, functional, and molecular level details, can be obtained. The design of multimodal imaging materials often involves the integration of multiple functional components. For instance, MNPs can be combined with fluorescent molecules, enabling the materials to be utilized for both MRI and fluorescence imaging. Furthermore, some materials can be engineered to respond to specific biomarkers or environmental conditions, such as pH or enzyme activity, facilitating more precise imaging. Yan et al. proposed a new strategy for constructing “activated” NIR fluorescence/MRI bimodal small molecule imaging probes by combining fluorescence-activated reactions and in situ self-assembly [150]. This strategy has been applied in high-resolution MRI and high-sensitivity NIR fluorescence imaging analysis in vivo.

#### Trilinear state materials

Singlet and triplet states represent the two primary electron spin configurations. Due to the symmetry-forbidden transition barrier preventing triplet-state return to the ground state, triplet-state materials exhibit novel photophysical properties distinct from singlet-state systems, yielding broad application potential in optoelectronics and biomedicine. These materials have emerged as “excitation-free” biomarker imaging probes, leveraging their capacity to capture and slowly release triplet-excited-state energy. Their ultra-long afterglow and near-infrared emission enable real-time-excitation-free operation, demonstrating unique advantages for biomarker detection. Through triplet–triplet annihilation upconversion (TTA-UC), such materials convert low-energy near-infrared light into high-energy visible emissions, effectively overcoming biological tissue light scattering and autofluorescence interference to significantly enhance imaging penetration depth and signal-to-noise ratio [151].

Recent advances in organic–inorganic hybrid polymers (e.g., anthracene-derivative-containing systems) maintain high-efficiency solid-state TTA-UC while offering excellent photostability, tunable emission wavelengths, and biocompatibility–enabling novel strategies for high-resolution deep-tissue imaging [151]. Surface functionalization with targeting molecules (e.g., antibodies, peptides) further permits specific biological target recognition and real-time monitoring of TMEs and inflammatory markers [152]. Despite these promising characteristics, research on triplet-state materials remains limited, warranting expanded investigation.

#### Imaging biomaterials Summary

Biomaterials have revolutionized the field of biomarker imaging by providing diverse platforms tailored to specific imaging modalities. Fluorescent materials (organic molecules, nanoparticles, AIEgens) provide high sensitivity and specificity for optical imaging, with AIEgens overcoming the problem of aggregation-induced bursting. Radioisotope labeling materials enable sensitive PET/SPECT imaging and targeted radiation therapy. Magnetic nanomaterials (e.g., iron oxide, gadolinium complexes) serve as powerful MRI contrast agents. Multimodal imaging materials integrate multiple functions, provide complementary information, and contribute to a more complete understanding. The design and synthesis of these materials focuses on enhancing targeting, signal strength, biocompatibility, and stability. Future work must prioritize improving the in vivo performance of these complex imaging agents, reducing potential toxicity, and facilitating their clinical translation (Table 2). However, the current development of bioimaging and detection materials faces multiple technical bottlenecks. Fluorescent materials are difficult to balance high quantum efficiency and long-term stability, and are often interfered by photobleaching and tissue autofluorescence [153]; radiolabeled materials are prone to nuclide leakage due to the limitations of nuclide half-life matching and in vivo stability of chelating agents [154]; magnetic nanomaterials suffer from the tension between the increase of relaxation rate and biosafety, and the risk of metal ions release and immunogenicity impede the application [155]; and multimodal probes suffer from different imaging and clinical translations. The multi-modal probes encounter the problem of incompatible signal strengths and penetration depths of different imaging modalities (e.g., optical/MRI), and it is difficult to ensure batch consistency in large-scale production [156]. In order to promote the practical application of bioimaging and detection technologies, future research should focus on breaking through the above bottlenecks by improving the stability and signal-to-noise ratio of fluorescent probes, ensuring the safety and accuracy of radiolabeling, resolving the biosafety of magnetic nanomaterials, and overcoming the compatibility and batch production problems of multimodal probes.

**Table 2:** Biosensing Materials: Mechanisms, Advantages, and Biomedical Applications.

Material Class	Subtype	Detection Mechanism	Key Advantages	Applications in Biomarker Detection	References
Nanomaterials	Metal Nanoparticles (Au, Ag)	SPR enhanced signal Catalytic activity	High conductivity Easy functionalization Enhanced electrochemical/optical signals	The lateral flow immunoassay technology mediated by bimetallic nanoenzymes for highly sensitive detection of cancer markers.	[160]
	CNTs	Surface charge transport characteristics One-dimensional structure sensitive to environmental changes	High specific surface area High mechanical strength Suitable for electrochemical sensors	Single-Walled Carbon Nanotubes (SWCNTs) chemical resistance sensors detect citrus Huanglongbing secretory proteins; PA/PANI-CNT electrochemical sensors detect Aβ42.	[173]
	QDs	Narrowband emission Photoluminescence Multicolor labeling	Stokes shift is large Light stability is superior to that of traditional dyes	Three-color QD nanobead multiplex detection technology; Graphene QDs for synchronous detection of DNA targets.	[184,185]
	MOFs	Porous structure for capturing analytes Ligand functionalization	High specific surface area Customizable pore structure Easy to couple with biological molecules	The MOF-ELISA platform is used for ultra-sensitive detection of PD-L1; lanthanide MOFs are used for the detection of TMAO.	[187,189]
Bioactive Materials	Antibodies/Nanobodies	Antigen-antibody specific binding	High affinity Strong penetration of nanobodies	68 Ga-labeled HER2 nanobody PET imaging for breast cancer; VHH-136 nanobody for detecting MMP-2.	[191,195]
	Enzymes/Nanozymes	Catalyze the generation of detectable signals from the substrate	Signal amplification capability Sensitivity improvement of single-atom nanoenzymes	Fe-N-C single-atom nanozyme detects Aβ1-40; CuCoFe layered double hydroxide mimics peroxidase to detect markers of pheochromocytoma.	[196,197]
	Nucleic Acid Aptamers	Adaptor-target specific folding binding.	Easy to synthesize Resistant to denaturation Designable anti-nuclease sequences	ssDNA aptamer for detecting CA125 in ovarian cancer; electrode design for inhibiting CypB degradation.	[199,200]
Polymers	Molecularly imprinted polymers (MIPs)	Template molecular imprinting forms specific recognition cavities	Resistant to environmental interference Low cost Reusable	Surface-enhanced Raman spectroscopy-MIP technology for detecting tumor markers.	[205]
	Hydrogels	Three-dimensional network-fixed biological molecules Intelligent responsive release signals	High biocompatibility Suitable for wearable devices	miRNA hydrogel sensor can detect Alzheimer's disease without enzymatic amplification; semiconductor hydrogel can in situ amplify bioelectric signals.	[209,210]
	Conductive Polymers	Biological recognition events alter electrical conductivity	Flexible electrode materials Easy to integrate into sensing systems	Poly-pyrrole-poly-dopamine/AuNPs complex for detecting carcinoembryonic antigen (CEA); SWCNT-conductive polymer nanocomposite for impedance detection of calretinin.	[226,227]
Composite Materials	Nanomaterial-Bioactive Hybrids	Nanomaterials loaded with biological recognition elements	Synergistic enhancement of sensitivity/selectivity	Functionalized polypyrrole with gold nanoparticles is used to detect CEA.	[226]
	Nanomaterial-Polymer Composites	Polymer matrix dispersion of nanomaterials Increase the active surface area	Mechanical flexibility Can be scaled up for production	rGO/Au nanocomposite material for detecting miRNA-122.	[158]

### Biomaterials for biomarker detection

Biomaterials for biomarker detection are the core functional components for building biosensors or sensing platforms (Table 3). The core role of these materials is to realize specific recognition and signal conversion and amplification. Specifically, they make use of their own bioactivity or biomimetic recognition properties to capture and bind target biomarkers with high selectivity; at the same time, they are able to efficiently convert such biorecognition events into measurable physical or chemical signals (e.g., electrochemical signals, such as changes in current, voltage, and impedance, and optical signals, such as changes in absorbance, fluorescence intensity, color, and mass, etc.), and often make use of the special properties of the materials themselves (e.g., the catalytic activity of nanomaterials) to achieve specific recognition and amplification. The detection signals are often enhanced by the special properties of the materials themselves (e.g., catalytic activity of nanomaterials, high surface area, electrical conductivity) or by combining them with signal amplification strategies, which ultimately leads to the goal of qualitative detection and accurate quantitative analysis of biomarkers with high sensitivity and specificity.

**Table 3:** Summary of Key Biomarkers with Detection/Imaging Technologies and Biological Significance.

Biomarker	Detection/Imaging Technology	Biomaterial Used	Biological Significance	References
CEMA, 4-ABP (tobacco exposure)	Urinary metabolite assay	Not specified	Reflects absorption & metabolism of harmful substances in smokers; used to assess tobacco exposure dose	[38]
MN frequency	Cytokinesis-block micronucleus test	Not specified	Indicates chromosome damage induced by VC; serves as an effective-dose biomarker	[42]
Phosphorylated α-synuclein	Immunohistochemistry/ELISA	Antibody-labeled probes	Early diagnosis of Parkinson’s disease; distinguishes from other parkinsonian disorders	[62]
cTnI (cardiac troponin I)	Electrochemical immunosensor	Conductive polymer/Au nanoparticles	Early diagnosis and prognosis of myocardial infarction	[216]
BNP/NT-proBNP	Immunofluorescence/ELISA	Not specified	Diagnostic and therapeutic monitoring markers for heart failure	[71,72]
CA125, CEA	Fluorescence-linked immunosorbent assay (FLISA)	QDs	Early screening and treatment efficacy evaluation for ovarian and gastric cancers	[184]
Aβ42, p-tau181	Single-molecule array (Simoa)	Antibody-coated magnetic beads	Early diagnosis and progression monitoring of AD	[268,269]
miRNA	Fluorescent probe hybridization	DNA probe-fluorophore conjugates	Early cancer diagnosis and subtyping; reflects aberrant gene expression	[16,91]
CD47 (atherosclerosis)	NIR fluorescence imaging	AIE-active nanoparticles	Plaque localization in atherosclerosis and drug screening	[246]
VEGF (retinopathy)	OCT imaging	Ag_2_S nanoparticles	Early diagnosis of diabetic retinopathy (DR) and detection of vascular abnormalities	[262]
STAT3 hypomethylation	DNA methylation assay	PCR probes	Early toxicity marker for benzene exposure; indicates epigenetic alterations	[53]
TgAb + cytokines	Immunoassay	Antibody cocktail	Predicts thyroid dysfunction triggered by immunotherapy	[54]
IGFBP7	Mass spectrometry/ELISA	Not specified	Diagnostic and progression-prediction marker for MS	[63]

#### Nanomaterials

Nanomaterials are synthesized by physical, chemical and biological methods. Physical synthesis includes methods such as gas deposition, electron beam lithography, pulsed laser stripping, laser-induced pyrolysis, powder ball milling and aerosols. Chemical synthesis includes methods such as co-precipitation, microemulsion, hydrothermal, electrochemical deposition, sonochemistry and thermal decomposition. Biosynthesis includes fungal-mediated, algal, bacterial-mediated, and yeast-mediated methods [157]. Nanomaterials are playing an increasingly important role in biomarker detection, with their unique physicochemical properties enabling a wide range of applications in medical diagnostics. These properties include the small size effect, high specific surface area, surface effect, quantum size effect, macroscopic quantum tunneling effect, and dielectric confined domain effect. As a result, nanomaterials exhibit significant differences from conventional materials in terms of melting point, vapor pressure, optical properties, chemical reactivity, and magnetic properties. For instance, MNPs and metal oxide nanoparticles are commonly employed for signal amplification and optical detection due to their distinctive optical and electrical properties. Additionally, carbon nanotubes are well-suited for constructing electrochemical sensors, enhancing the selectivity and sensitivity of detection due to their excellent electrical conductivity and mechanical properties.

##### Metal nanoparticles and metal oxide nanoparticles

MNPs and Metal Oxide Nanoparticles (MONs) have demonstrated significant potential and applications in biomarker detection due to their unique physicochemical properties [158]. These nanomaterials enhance the sensitivity and selectivity of biosensors and are widely utilized in electrochemical, optical, and SPR biosensors (Fig. 7C). MNPs typically refer to aggregates of metal atoms with sizes ranging from 1 to 100 nm, primarily including gold nanoparticles and silver nanoparticles, among others. They exhibit physicochemical properties such as high specific surface area, high porosity, and low thermal conductivity. Conversely, MONs include materials such as zinc oxide (ZnO) [159], titanium oxide (TiO_2_), and iron oxide (Fe_3_O_4_). In biomarker detection, MNPs and MONs can serve as signal amplification tags, catalysts, or bioprobes to enhance the performance of biosensors. For instance, gold nanoparticles can augment SPR signals for ultra-sensitive biomarker detection. Additionally, MONs have been employed in the preparation of high-performance electrochemical sensors for the detection of various biomarkers, including glucose, lactate, dopamine, and ascorbic acid. Meng et al. developed a strategy based on bimetallic nanoenzymes-mediated in situ catalytic deposition of reporter molecules for lateral flow immunochromatography, achieving ultrasensitive detection of cancer biomarkers [160]. This method successfully identified positive gastric cancer samples from clinical serum specimens. Notably, Yang et al. developed a facile photochemical method to prepare luminescent gold nanoparticles (L-AuNP@HTT), coated with 2-hexylthio-1,3,4-thiadiazole-5-thio, which exhibited excellent photoluminescence properties and two-photon absorption cross-sections, providing a new tool in the field of bioimaging [161]. However, despite the great potential of metal oxide nanomaterials and their nanocomposites for electrochemical sensors and biosensor platforms, further exploration is required to address challenges such as stabilizing multiple active sites, optimizing analyte adsorption, and securing adsorption of intermediate products, as well as developing reusable sensor platforms.

##### Nanofibers

Nanofibers have a wide range of potential applications in biomarker detection due to their unique physical and chemical properties. Characterized by high specific surface area, porosity, and high loading capacity, nanofibers are particularly useful for immobilizing recognition elements, directly interacting with target analytes, enhancing antibody immobilization, and improving the activity and lifetime of biomolecules [162]. These advantages enable the development of nanofiber-based biosensors for the highly sensitive and selective detection of major disease biomarkers. Currently, the application of electrostatically spun nanofiber biosensors in biomarker detection has been extensively studied, demonstrating their capability for detecting and analyzing various disease biomarkers, including those related to cancer, CVD, and neurological disorders [162]. Additionally, nanofibers have been employed to enhance the stability and activity of enzymes, which is critical for sensitive biomarker detection. For instance, Chenafa et al. immobilized β-galactosidase in polystyrene electrospun nanofiber membranes incorporating functionalized graphene oxide, resulting in significantly improved adsorption capacity, stability, and activity of the enzyme [163]. This technique can be extended to the detection of other enzymes and biomarkers, offering new possibilities for biomedical diagnosis. Notably, Zhou et al. utilized engineered fd phage nanofibers—safe viral nanofibers equipped with peptide fragments that target specific biomarkers—for the detection of cervical cancer biomarkers [164]. This approach demonstrated higher sensitivity than the traditional ELISA method, providing new ideas and methodologies for the diagnosis and treatment of cervical cancer.

##### Carbon nanotube

Carbon nanotube (CNT) has demonstrated significant potential in biomarker detection due to their outstanding electronic, structural, and optical properties [165]. The common types of CNT include SWCNTs [166], Multi-Walled Carbon Nanotubes (MWCNTs) [167], Few-Walled Carbon Nanotubes [168], and Double-Walled Carbon Nanotubes [169]. Among these, SWCNTs and MWCNTs are primarily utilized in electrochemical and electrical applications [170] (Fig. 7D). Recently, researchers have developed a variety of carbon nanotube-based sensors for efficient and sensitive biomarker detection, which is crucial for the early diagnosis and treatment of diseases [171]. In electronic sensors, carbon nanotubes are integrated into chemoresistors and field-effect transistors to detect minute biomolecules. The operation of these sensors relies on the one-dimensional structure of carbon nanotubes and the charge transport properties on their surface, making them highly sensitive to environmental changes [172]. For instance, Tran et al. developed a SWCNT-based chemoresistive biosensor for detecting secreted protein biomarkers associated with citrus Huanglong disease, highlighting the potential of carbon nanotubes for biomarker detection [173]. Moreover, Galassi et al. demonstrated the long-term biocompatibility of SWCNTs in vivo, suggesting their viability as a preclinical research tool without adverse short- or long-term health effects [174]. This finding is critical for understanding the biocompatibility of carbon nanotubes in future applications, particularly in biomarker detection. Additionally, Kim et al. developed a method for detecting ovarian cancer using quantum defect-modified carbon nanotubes in serum samples [175]. By employing machine learning and spectral fingerprinting techniques, the researchers achieved high sensitivity and specificity in detecting ovarian cancer biomarkers. This work not only illustrates the potential of carbon nanotubes in disease diagnosis but also provides a new direction for future biomarker detection. Thus, carbon nanotubes show significant promise in biomarker detection, particularly for biomarkers associated with metabolic diseases and cancer. Nonetheless, to fully harness their potential in clinical biomarker detection, future research must delve deeper into this area.

##### Quantum dots

QDs are low-dimensional semiconductor nanocrystals composed of group II-VI (e.g., CdS, CdSe, ZnS) or III-V (e.g., InP, InAs) elements that exhibit significant size effects within a diameter range of 2 to 20 nm, endowing them with unique physical and chemical properties [177,178]. The optical characteristics of these nanocrystals, including broad excitation spectra, excellent photochemical stability, and long fluorescence lifetimes, allow them to provide higher fluorescence intensity and stability in fluorescent labeling compared to conventional fluorescent dyes, resulting in increased sensitivity in detection and diagnostics [176], [179], [180] (Fig. 7A). Additionally, QDs can efficiently detect multiple protein biomarkers and viruses in blood samples simultaneously. Their simple detection methods and high cost-effectiveness [181] have led to preliminary applications in medical testing. For instance, the fluorescence immunosorbent assay utilizes QDs to replace enzymes in conventional ELISA, achieving highly sensitive detection of biomarkers, proteins, and small-molecule toxins. This method not only enhances the high-throughput capability of the assay but also ensures accuracy and reproducibility of the results [182]. Beyond that, the photoluminescence (PL) properties of QDs can be effectively modulated through a core–shell structure, allowing for narrow-band emission and photochemical stability, which has been realized in various QD material systems [183]. Consequently, QDs have been widely employed as key active ingredients in diverse applications, including solar cells, light-emitting diodes, and photodetectors. For example, Lv et al. developed customized tricolor QD nanobeads for multiplexed assays with tunable detection ranges and signal amplification immunosensors exhibiting multilevel sensitivity, demonstrating the potential of QDs for multiplexed biomarker detection [184]. Moreover, graphene QDs have shown exceptional potential for detecting cancer biomarkers with high sensitivity and efficiency due to their unique physicochemical properties. Wang et al. developed a dual-color graphene QD and carbon nanoparticles biosensing platform combined with nucleic acid exonuclease III-assisted signal amplification for the simultaneous detection of multiple DNA targets, providing a promising approach for DNA biomarker detection [185]. However, the biocompatibility, potential toxicity, and long-term stability of QDs remain significant obstacles to their widespread clinical applications. Further research is needed to advance the development of QDs for biomedical applications.

##### Metal-organic frameworks

**Fig. 8:**
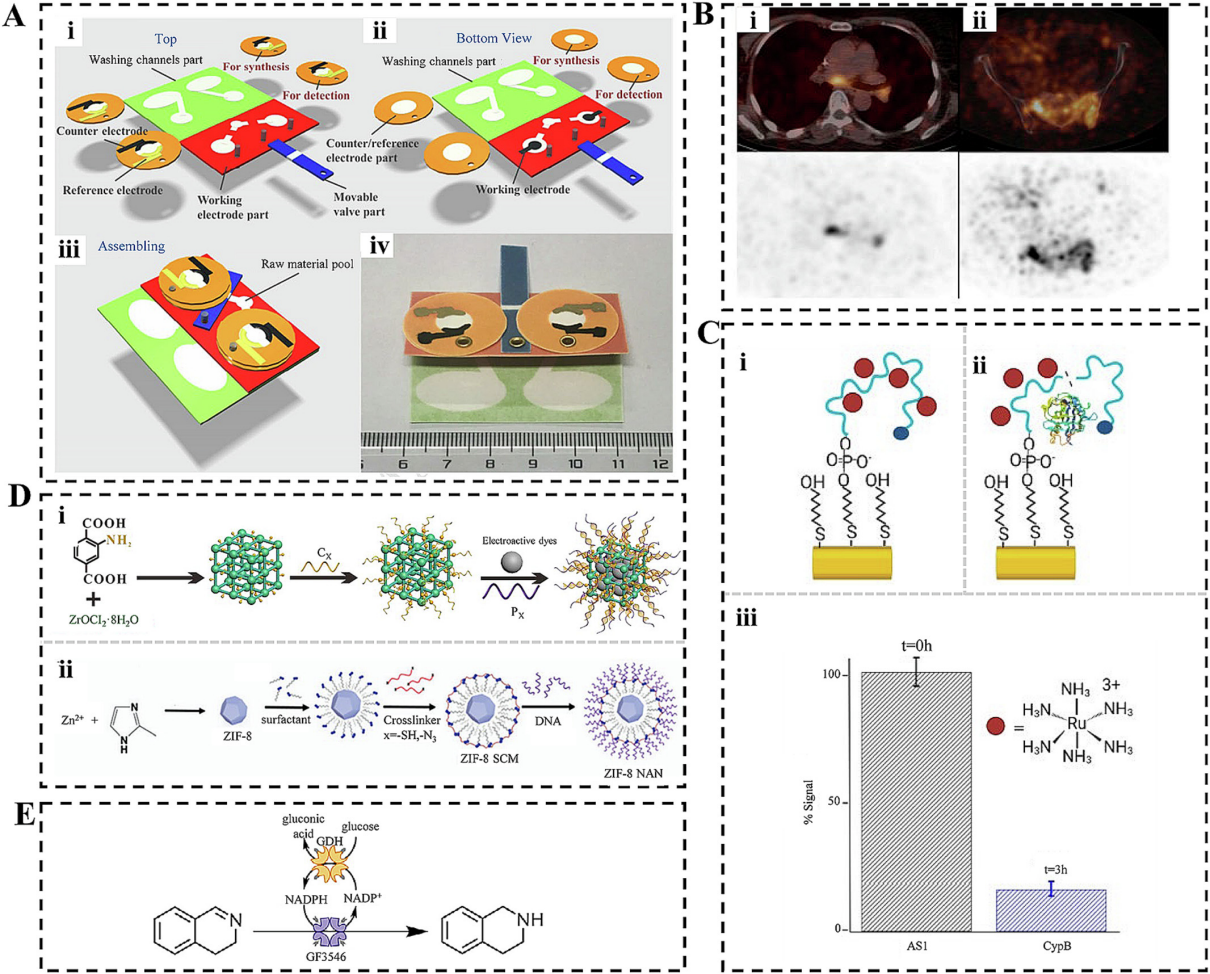
Application of biomaterials in biosensor design and molecular detection technologies. (A) 3D structure and components of Bio-MIP-ePADs. (i) Top view of the Bio-MIP-ePAD assembly, showing the working electrode (red), counter/reference electrode (yellow), flushing channel (green), and removable valve (blue). (ii) Elevation view of the same assembly. (iii) 3D schematic of the fully integrated Bio-MIP-ePAD, illustrating the synthetic material cell and the sample cell connected via removable valves. (iv) Top view of the constructed Bio-MIP-ePAD, along with photographs of dimensional measurements [202]. (B) PET/CT and PET images of 68 Ga-HER2 nanobodies in metastatic lesions. (i) Lymph node invasion observed in the mediastinum and left hilar region of the patient. (ii) Pelvic bone metastases in patient 20 [191]. (C) Effect of CypB on surface DNA concentration of nucleic acid-based electrochemical sensors (NBEs). (i) DNA surface concentration was assessed by incubating NBEs in a ruthenium hexamine buffer solution. (ii) Electrostatic binding of redox molecules to the DNA backbone. (iii) Cleavage of the DNA strand leads to the removal of ruthenium hexamine molecules from the surface. The percentage of ruthenium hexamine molecules was measured by chrono-coulometry for freshly fabricated NBEs and NBEs treated with 500 nM CypB, with a waiting time of 2 h before DNA quantification [200]. (D) Schematic representation of covalent binding of DNA and MOFs. (i) Formation of an amide bond between the amino group on UIO-66 and the carboxyl group at the end of the nucleic acid. (ii) Covalent immobilization of nucleic acids facilitated by surfactants and cross-linkers [186]. (E) All-enzyme hydrogel for the reduction of imines to amines, accompanied by cofactor regeneration [206]. (For interpretation of the references to color in this figure legend, the reader is referred to the web version of this article.)

MOFs are a class of porous crystalline materials formed by metal ions or metal clusters connected to organic ligands through strong coordination bonds. MOFs possess a unique pore structure and tunable chemical properties. The high specific surface area and porous structure of MOFs facilitate increased interactions between the material and analyte interfaces, along with active sites. Moreover, the organic ligands of MOFs carry abundant functional groups, enabling their easy functionalization with various molecules and materials, including nucleic acids, enzymes, and nanoparticles [186] (Fig. 8D). As a result, MOFs have been widely applied in biomarker detection, encompassing immunodetection, chemical sensing, fluorescence sensing, and more. For instance, a significant advancement in the application of MOFs has been achieved in immunodetection. Zhand et al. developed an innovative MOF-based ELISA platform capable of ultra-sensitive detection of programmed cell death ligand 1 (PD-L1), a key immune checkpoint molecule that plays a crucial role in the immunotherapy of many cancers [187]. In the realm of chemosensing, MOFs have also demonstrated unique applications. Qu et al. successfully utilized [NH_2_ (CH_3_)_2_] _2_-[Cd_3_._5_ (bdba)(Hbdba)(H_2_O)_1_._5_]_n_ (Cd-MOF) for the chemosensing of diphenyl phosphate as a flame retardant biomarker, highlighting the structural characterization and stability analysis of MOFs [188]. Additionally, in fluorescence sensing, Min et al. achieved the successful detection of trimethylamine-N-oxide, a biomarker challenging to detect using UV–vis spectroscopy, through the use of lanthanide MOFs via an outer sphere interaction strategy [189]. The applied research of MOFs in biomarker detection is rapidly developing, and their versatility and tunability offer new tools and methods for biomedical diagnosis. Future research may focus on developing newly developed MOF materials with improved stability, biocompatibility, and enhanced sensitivity and selectivity [190]. Researchers are also expected to explore the integration of MOFs with other technologies to facilitate faster and more convenient biomarker detection methods. Additionally, developing regeneration methods applicable to MOFs and reducing the cost and complexity of MOF synthesis are crucial directions for future research.

#### Bioactive materials

Bioactive materials are a class of substances that can trigger specific biological reactions upon contact with living organisms, playing a crucial role in biomarker detection. These materials include, but are not limited to, antibodies, nucleic acid aptamers, MIPs, and enzymes, all of which are capable of specifically recognizing and binding to target biomarkers. Thus, they are integral to biosensing, clinical diagnosis, and disease monitoring. Among these materials, antibodies rely on specific binding generated by the immune system, MIPs are synthetic polymers that mimic the recognition functions of antibodies, and enzymes utilize their catalytic activity to enhance detection signals. Each of these categories has its unique advantages in biomarker detection.

##### Antibodies

Antibodies are an important class of biologically active molecules produced by B cells that differentiate into plasma cells in response to antigenic stimulation. They are a type of immunoglobulin that specifically binds to particular antigens. With advancements in biotechnology, various types of antibodies are now employed in biomarker detection. Currently, both monoclonal and polyclonal antibodies have become well-established in this field. Monoclonal antibodies are generated from a single B-cell clone, resulting in a high degree of homogeneity and specificity. They are widely used in clinical diagnosis, particularly in specific immunohistochemistry (IHC) testing. Conversely, polyclonal antibodies are mixtures produced by multiple B-cell clones that recognize several different antigenic epitopes and are commonly utilized in methods such as ELISA. However, the large molecular weight of both monoclonal and polyclonal antibodies may limit their ability to penetrate cells and tissues. In contrast, nanoantibodies are characterized by strong tissue penetration and efficient metabolism, enabling precise targeting of cells and molecules. Additionally, nanoantibodies are applied in various imaging techniques, such as PET [191] (Fig. 8B), SPECT [192], and optical imaging [193]. This versatility offers significant prospects for applications in the fields of bioimaging and detection, positioning them as a current research hotspot. For example, Deng et al. developed a technique for the directed immobilization of nanoantibodies on polystyrene surfaces, facilitating efficient immobilization on solid-phase carriers while preserving protein activity and spatial structural integrity [194]. Marturano et al. identified a nanoantibody (VHH-136) that does not inhibit the activity of matrix metalloproteinase-2 (MMP-2) and is chemically coupled to a fluorescent probe [195]. This design allows for the detection of human MMP-2 expression using flow cytometry and immunocytochemistry. Consequently, nanobodies have a wide range of applications in biomarker detection and tumor imaging, and as research progresses, they are expected to demonstrate significant application value across more fields in the future.

##### Enzymes

Enzymes are proteins that catalyze specific chemical reactions in living organisms. They are highly selective, recognizing and binding to target biomarkers with precision. The binding of an enzyme to a biomarker initiates a signaling process that converts the biorecognition event into a measurable signal. Simultaneously, the catalytic activity of the enzyme can enhance the detection signal, particularly in the development of newly developed biosensors. Currently, researchers primarily utilize nanomaterials and nanoenzymes to improve the sensitivity and specificity of detection. For instance, Lyu et al. developed Fe-N-C single-atom nanoenzymes, which substantially enhanced the detection sensitivity of amyloid β1-40, achieving an ultra-low detection limit [196]. Similarly, Huang et al. reported the use of CuCoFe layered double hydroxide as laccase-mimicking nanoenzymes for the colorimetric detection of pheochromocytoma biomarkers [197]. This demonstrates that nanostructured mimetic enzymes possess significant potential for optimizing biomarker detection. Notably, Neda Rafat et al. introduced an innovative approach for the multipoint-of-care electrodetection of neocoronavirus pneumonia biomarkers through enhanced enzymatic amplification and metallization on nanostructured surfaces [198]. The crux of this approach lies in the generation of dense and conductive localized surface silver metallization on microelectrode arrays by combining an enzymatically labeled probe for the biomarker with a nanostructured catalytic surface that selectively reduces a soluble silver substrate. This method permits sensitive, quantitative, simple, and direct electronic readout of biomarker binding without the need for intermediate optics, providing a new direction for related research.

##### Nucleic acid aptamers

Nucleic acid aptamers are a class of single-stranded DNA or RNA molecules that can be screened from a large library of randomly sequenced oligonucleotides with high affinity and specificity for a particular target molecule through a systematic evolutionary process. The primary method for screening aptamers is Systematic Evolution of Ligands by Exponential Enrichment (SELEX) technology. Using SELEX, aptamers can be isolated from oligonucleotide libraries containing numerous random sequences capable of specifically binding to a target molecule. These aptamers can detect and quantify a variety of biomolecules, including proteins, nucleic acids, and small molecules, providing a rapid, sensitive, and cost-effective assay. Currently, nucleic acid aptamers are primarily used in early cancer detection and diagnosis; for example, the single-stranded DNA (ssDNA) ligand developed for the ovarian cancer biomarker CA125 has demonstrated promising diagnostic potential [199]. Additionally, aptamers serve as recognition elements that specifically bind to target molecules, triggering signal converters to produce detectable signals. For instance, Clark et al. explored the nuclease activity of human-derived cyclosporin B (CypB) and used an ultrasensitive nucleic acid-based electrochemical sensor to confirm that purified CypB could hydrolyze electrode-bound ssDNA in buffer solutions of physiological pH and ionic strength, as well as facilitate double-stranded DNA/DNA and DNA/F-RNA hybridization [200]. The findings of this study bear significant implications for the development of aptamer sensors. Given that CypB exhibits nuclease activity, future CypB bioassay development will necessitate the use of nuclease-resistant aptamer sequences (Fig. 8C). Consequently, when designing aptamer sensors for CypB detection, it is essential to consider the potential degradation of the nucleic acid portion of the sensor by CypB. This consideration underscores the need for aptamers capable of resisting nuclease activity, presenting a new challenge and research direction for the application of aptamer sensor technology in biomarker detection.

#### Polymers

Polymers primarily consist of polymeric compounds of both natural and synthetic origins, typically including materials such as MIPs, hydrogels, and conductive polymers. These polymers are generally biocompatible, allowing them to interact safely with human tissues and blood. As a result, polymers can serve as substrates for immobilizing bioactive molecules, facilitating the development of high-performance biosensors. Additionally, they can function as signaling agents to convert biometric events into measurable electrical or optical signals. With advancements in materials science and nanotechnology, it is anticipated that a greater variety of polymeric materials will be developed in the future to meet the demands of clinical diagnostics and biomedical research.

##### Molecularly imprinted polymers

MIPs are synthetic materials designed to replicate the mechanisms of antibody-antigen interactions found in nature. Typically, MIPs refer to polymers with molecular recognition capabilities that are created by selecting a specific template molecule, inducing functional monomers to self-assemble around it, and utilizing cross-linking agents and catalysts during the process. Afterward, the template molecules are removed using eluents, resulting in recognition cavities that are complementary to the structure of the original template molecules. This process is akin to the fitting of a key into a lock, allowing MIPs to achieve highly specific recognition when the template molecule is encountered again. Currently, this capability poses challenges concerning the selectivity and sensitivity of sensors, particularly since analytes are often present at trace levels. In this context, enhancing the selectivity and specificity of molecular recognition elements in chemical sensors becomes particularly important. Consequently, molecular imprinting technology has emerged as a promising approach to improve the selectivity of sensor targets, and MIPs offer numerous advantages, including resistance to environmental changes, low cost, and straightforward synthesis processes [[201], [202], [203]] (Fig. 8A). The application of MIPs in biosensor technology is becoming increasingly widespread [204], encompassing a broad range of sensor types, including, but not limited to, optical molecularly imprinted sensors, electrochemical molecular imprinting sensors, and QCM sensors. These sensors are widely utilized in the detection of various biomarkers, such as cancer markers and viral markers. For instance, Lin et al. developed an interference-free, high-precision biosensor that combines surface-enhanced Raman spectroscopy with surface MIPs technology for the detection of tumor biomarkers in human blood [205]. Similarly, a MIPs-based electrochemical biosensor was employed to monitor bone loss by measuring the concentration of CTx-I in serum. However, MIPs still encounter several challenges in practical applications, such as the efficiency of template molecule elution, the stability of the polymers, and the feasibility of large-scale production—all of which require further investigation in the future.

##### Hydrogels

Hydrogels are three-dimensional polymer networks with a high capacity for water absorption [206]. They are typically formed by hydrophilic polymers through physical or chemical cross-linking (Fig. 8E) and can be categorized into natural and synthetic hydrogels. Natural hydrogels, such as sodium alginate and gelatin, exhibit excellent biocompatibility, while synthetic hydrogels, like polyacrylamide gels and polyamide gels, are known for their controllability and stable performance. Furthermore, hydrogels—especially smart-response hydrogels—can provide a stable microenvironment for bioactive molecules while facilitating molecular diffusion and sensing reactions [207]. In biomarker assays, hydrogels are commonly employed to immobilize bioactive molecules, enhancing their stability and preserving their bioactivity, which in turn improves the selectivity and sensitivity of the sensors. For example, Jinn et al. developed a bioresponsive hydrogel sensor that utilizes silica nanoparticles to analyze protein biomarkers through light interference, demonstrating significant potential for a variety of applications [208]. Additionally, Lim et al. created a miRNA sensing hydrogel for the early diagnosis of AD, showcasing its intrinsic signal amplification capabilities [209]. This hydrogel utilizes a probe that catalyzes a hairpin assembly reaction to amplify fluorescent signals without the need for enzymes or temperature changes, enabling highly sensitive detection of target miRNAs. Moreover, enhancing the mechanical and electrical properties of hydrogels opens new possibilities for biomarker detection. For instance, Li et al. conducted pioneering research on high-performance semiconductor hydrogels that possess excellent mechanical properties along with semiconductor characteristics, allowing for in situ high signal-to-noise amplification of bioelectrical signals [210]. Notably, hydrogels’ high water content, good biocompatibility, and softness—similar to human skin—make them ideal materials for use in wearable biomarker detection devices [211] and for developing flexible sensors that detect biomarkers in blood, sweat, saliva, and tears. These sensors utilize hydrogels as immobilization platforms for biomolecules, capturing and converting biomarkers into detectable signals through specific binding events [212]. In conclusion, the application of hydrogels in biomarker detection shows great promise, and with advancements in materials science, we can expect the development of even more hydrogel materials in the future to meet the needs of clinical diagnosis and biomedical research.

##### Conductive polymers

Conductive polymers are a class of polymeric materials characterized by conjugated bonds, which can be divided into composite and structural types [213]. Composite conductive polymers are created by adding conductive fillers to conventional polymers, and they have been widely used in applications such as antistatic materials and electromagnetic wave shielding. In contrast, structural conductive polymers are formed by doping conjugated polymers to achieve conductivity, with examples including polyacetylene and polyaniline. These materials exhibit excellent electrochemical activity and biocompatibility, making them ideal for immobilizing biomolecules and facilitating electron transfer. This type of material has potential applications in batteries, sensors, display technologies, and more [214]. In biomarker detection, conductive polymers are often utilized as part of the electrode material to immobilize biorecognition elements, such as antibodies or nucleic acid aptamers, through methods like adsorption, covalent bonding, or embedding. When specific biomarkers bind to the biorecognition elements on the electrode surface, the conductive polymer converts the binding event into an electrical signal, enabling the quantitative detection of the biomarker [215]. Furthermore, the applications of conductive polymers in electrochemical biosensors extend to the detection of cancer markers, CVD markers, inflammatory markers, and infectious disease markers. For instance, Gholami et al. developed an electrochemical immunosensor based on an antibody-coated conductive polymer for detecting cardiac troponin I (cTnI) in plasma [216]. Notably, integrating conductive elements into hydrogel systems can produce materials that possess both conductive properties and high water content, making them suitable for use in biomanufacturing technologies [217]. For example, Wang et al. investigated a stretchable, freeze-resistant conductive hydrogel reinforced with cellulose nanocrystals for wearable electronic devices [218]. Thus, conductive polymers play a crucial role across various fields, from biomedical applications to wearable electronic devices and electrochemical sensing. However, to fully realize the potential of conductive polymers in clinical biomarker detection, future research must further explore this area in depth.

##### Covalent organic frameworks

Covalent organic frameworks (COFs) are crystalline porous polymers constructed from organic monomers (e.g., benzene diboronic acid, melamine) *via* covalent linkages (boronic ester bonds, imine bonds, etc.). These materials exhibit exceptional properties including high specific surface areas, tunable pore sizes, and structural designability [219,220]. Through precise control of topology and functional groups, COFs achieve selective biomolecular adsorption, enabling highly sensitive detection through adsorption-induced optical/electrical property changes [221].

Nitrogen-rich COF structures (e.g., containing hydrazone bonds or triazine units) facilitate specific binding with metal ions (Cr^3+^, Hg^2+^) or biomolecules, triggering measurable fluorescence signal variations [222]. In electrochemical sensing, COF-conductive material composites (e.g., carbon nanotubes, gold nanoparticles) leverage porous architectures to immobilize recognition elements (antibodies, aptamers, enzymes), significantly enhancing detection performance [223]. For instance, Song et al. developed a COF-carbon nanotube composite electrochemical biosensor for neurospecific enolase (NSE) detection [224]. This sandwich-type configuration achieved a detection limit of 166.7 fg/mL with a linear range of 500 fg/mL–10 ng/mL. The carbon nanotube integration enhanced conductivity and surface area, enabling efficient antibody immobilization and accelerated electron transfer that amplified electrochemical signals for ultrasensitive NSE quantification.

Despite COFs' outstanding biocompatibility and structural tunability, practical implementation faces challenges including poor aqueous dispersibility and limited scalability [221,225]. Future research should focus on optimizing biointerfacial properties via surface modification (e.g., hydrophilic group incorporation) and composite design, while advancing miniaturization and integration strategies to accelerate clinical translation.

#### Composite materials

Composite materials hold significant promise for applications in biomarker detection, as they can substantially enhance the performance of biosensors by integrating the benefits of various materials. Typically composed of two or more distinct materials, these composites exhibit differing physical or chemical properties on a macroscopic level while maintaining their unique characteristics at the microscopic level. This enables a synergistic effect that improves overall performance. The primary types of composites commonly utilized in biomarker assays include nanomaterial-bioactive material composites and nanomaterial-polymer composites. Nanomaterial-bioactive material composites incorporate nanomaterials—such as MNPs, carbon nanotubes, and QDs—with bioactive materials like antibodies, enzymes, and nucleic acid aptamers. This combination enhances the sensitivity and selectivity of biosensors. For instance, Wang et al. developed an electrochemical immunosensor based on a polypyrrole-polydopamine complex functionalized with gold nanoparticles for the detection of CEA [126]. This composite exhibits excellent electrical conductivity, water dispersibility, and adhesion, allowing it to form a homogeneous, stable, and biocompatible conductive film. Highly specific detection of CEA was achieved by immobilizing the oncogenic antibody through the unique interaction between the gold nanoparticles and the antibody. Nanomaterial-polymer composites not only increase the active surface area of the biosensor by incorporating nanomaterials like carbon nanotubes and graphene with polymers but also enhance sensitivity and selectivity through signal amplification strategies. For example, Aydın et al. developed a SWCNT-based conducting polymer nanocomposite for constructing an ultrasensitive label-free impedance immunosensor aimed at detecting calreticulin biomarkers in human serum samples via electrochemical impedance spectroscopy [227]. As advancements in science and technology progress, the application of composites in biomarker detection is expected to broaden significantly. Future research may focus on developing high-performance composites and multifunctional integrations that will facilitate the advancement of personalized medicine.

#### Summary of detecting biomaterials

Diverse biomaterials underpin the development of highly sensitive and specific biosensors for biomarker detection. Nanomaterials—including MNPs, MONs, nanofibers, carbon nanotubes, QDs, and MOFs—offer high specific surface area, unique electronic/optical properties, and signal amplification capabilities. Bioactive materials (antibodies, enzymes, nucleic acid aptamers) deliver precise molecular recognition, while polymers (molecularly imprinted polymers, hydrogels, conductive polymers) provide versatile platforms for immobilization, signal transduction, and biocompatibility. Composite materials synergistically integrate these advantages to enable biosensors with enhanced sensitivity, selectivity, stability, and potential for miniaturization and point-of-care applications. Key challenges include stabilizing biorecognition elements (especially enzymes/antibodies), minimizing nonspecific binding, ensuring long-term reliability, scaling production, and enhancing in vivo biocompatibility. Critical bottlenecks involve nanomaterial instability from protein adsorption/aggregation in physiological environments even after modification [228,229]; activity attenuation of bioactive molecules during immobilization where modifications often mask functional sites [230]; and environmental interference (pH, ionic strength) compromising polymeric response specificity [231]. These limitations stem from intrinsic material constraints (efficiency-stability tradeoffs, sensitivity-safety conflicts), biological complexity (nonspecific interactions, metabolic clearance), and engineering challenges (multicomponent integration, scalable quality control), necessitating interdisciplinary innovation for transformative breakthroughs.

### Clinical applications of biomaterials for biomarker detection and imaging

Biomaterials are reshaping the clinical diagnosis and treatment landscape with unprecedented depth, from early screening and targeting of tumors to real-time tracing of cardiovascular plaques, from non-invasive monitoring of diabetes to molecular-level imaging of retinal and neurodegenerative diseases, which have been pulled into the era of “early detection, precise localization, and tracking” of major diseases with higher sensitivity, higher resolution, and stronger targeting. Precision medicine era of “early detection, precise localization and traceability (Table 4).

**Table 4:** Applications of biomaterials in the imaging and detection of clinical disease biomarkers.

Clinical application	Examples of biological materials	Technical methods	Performance advantages	Potential challenges	References
Oncotherapy	QDs, Gold Nanoparticles, Carbon Nanotubes, Multifunctional Nanocomposites	Fluorescence Imaging, MRI, PET	High Sensitivity and Specificity for Targeted Drug Delivery and Imaging	Possible Toxicity Risks, Long-term Safety Unknown	[[232], [233], [234], [235], [236]]
CVD	MONs, Gold Nanorods, Nanoparticles Modified with Targeting Peptides	US, CT, OCT	Excellent Biocompatibility Enhancing Imaging Contrast and Sensitivity	Need to Enhance Targeting, Potential Immune Reactions	[[240], [241], [242], [243], [244], [245]]
Diabetes	GOD-Modified Nanomaterials, Carbon Nanotubes, Conductive Polymers	Electrochemical Sensing, Optical Sensing	Rapid Response and High Sensitivity Detection for Real-time Monitoring	Stability and Reproducibility Need Boosting; Cost-effectiveness to Consider	[250,255]
Retinopathy	Silver Nanoparticles, Upconversion Nanoparticles, Biocompatible Polymer Materials	OCT, Fluorescence Imaging	High-resolution Imaging with Good Biocompatibility and Safety	Need Deeper Imaging, Avoid Potential Phototoxicity	[[257], [258], [259], [260], [261], [262], [263]]
AD	Aβ Oligomer-Specific Antibodies, Tau Protein Phosphorylation-Specific Antibodies, Neuroinflammation-Related Protein Antibodies	ELISA, Chemiluminescent Immunoassay, Single-molecule Array Technology	High Sensitivity and Specificity, Multiple Biomarker Detection Capability	Invasive Lumbar Puncture Required for CSF, Blood Test Sensitivity to Be Enhanced	[[264], [265], [266], [267], [268], [269],^271^,^272^]

#### Oncotherapy

Tumors are complex diseases characterized by uncontrolled cell proliferation and invasive growth [232]. Treatment options include surgery, chemotherapy, radiotherapy, immunotherapy, and more. Key biomarkers in tumor therapy encompass tumor protein markers (e.g., CEA, CA125), gene mutations (e.g., KRAS, EGFR), circulating tumor cells, and circulating tumor DNA [233]. In oncology therapeutics, detection technologies for these key biomarkers have progressed significantly, evolving from traditional ELISA [234] to advanced techniques such as mass spectrometry, flow cytometry, IHC, and various molecular biology methods. These technological innovations have markedly enhanced the sensitivity and specificity of tumor biomarker detection, thereby playing a crucial role in the early diagnosis of tumors, monitoring therapeutic responses, and prognostic assessments. Currently, tumor diagnosis primarily involves comparing their signal differences with those of healthy tissues, utilizing a range of high-precision imaging techniques, including CT, PET, MRI, and Raman imaging [235]. Metallic nanomaterials have gained widespread use in CT imaging due to their excellent X-ray absorption, malleability, and ease of surface modification [236]. For instance, Wang et al. demonstrated that coating gold nanomaterials with silica on their surface significantly improved the specificity of CT diagnoses for HCC [237]. Additionally, attaching anticancer drug molecules to these gold nanomaterials increased the sensitivity of radiotherapy. While both CT and MRI provide contrast distribution in imaging, MRI has the advantage of producing tomographic images in any direction, including three-dimensional body images. Incorporating nanomaterials into MRI techniques can notably enhance their sensitivity and accuracy. MNPs have been particularly extensively studied in this area. For example, Chee et al. achieved a more effective T2-weighted image contrast agent by enriching superparamagnetic iron MONs with 86 polytitaniums, which improved liver contrast and increased the detection rate of HCC [238]. Furthermore, radioisotope-labeled metallic materials have demonstrated significant potential in PET imaging. Lee et al. utilized polyethyleneglycol to label gold nanomaterials with ^124^I (PEG-^124^I-Au@AuCBs) and verified their effectiveness in diagnosing tumors via PET through in vivo experiments [239]. These studies showed that the material did not adversely affect normal cells under different pH levels, serum conditions, and various simulated in vivo environments, demonstrating high stability and sensitivity in PET imaging of tumors. These findings indicate that the combination of biomaterials and advanced imaging techniques provides robust support for early tumor diagnosis. Future research may focus on developing newly developed biomaterials for precise tumor targeting, optimizing therapeutic efficacy, and minimizing side effects during treatment.

#### Cardiovascular disease

CVD is one of the leading causes of death worldwide, encompassing a wide range of disorders, including coronary artery disease, cardiomyopathy, heart failure, arrhythmia, and hypertension. The detection of key biomarkers for CVD is crucial for early diagnosis, monitoring therapeutic responses, and assessing prognosis. Major biomarkers include those indicating myocardial injury (e.g., cTnI, creatine kinase isoform MB), cardiac function markers (e.g., BNP), inflammatory markers (e.g., C-reactive protein), and oxidative stress markers [240]. Currently, traditional assays like ELISA [241] are being gradually replaced by more advanced technologies such as mass spectrometry [242], protein chip technology [243], and circulating miRNA assays [244], which have significantly improved the sensitivity and specificity of CVD detection. The application of biomaterials, particularly nanomaterials, in CVD imaging is becoming a focal point of research. Iron oxide-based nanoparticles have emerged as multifunctional tools for the diagnosis and treatment of atherosclerosis. They can serve not only as MRI contrast agents but also as secondary imaging components for multiple modalities, including PET, SPECT, CT, near-infrared fluorescence imaging, PAI, or US, through surface conjugation or ion doping [245]. Notably, Wang et al. developed a nanoprobe with high-brightness a AIE properties that specifically binds to CD47 molecules overexpressed in atherosclerotic plaques [246]. This technological advancement greatly enhances the accuracy and sensitivity of detecting atherosclerotic plaques while also providing a new tool for the efficient screening of anti-atherosclerotic drugs, which is significant for facilitating early diagnosis and drug discovery for CVD. Additionally, biosensors based on nanomaterials have gained widespread attention for the detection of CVD biomarkers due to their high sensitivity and specificity. For example, Vairaperumal et al. introduced an instant CVD biomarker detection technique using optical nanobiosensors, which are considered a promising method for rapid cardiac biomarker detection. These sensors benefit from easy monitoring, low cost, broad detection range, high sensitivity, and minimal interference [247]. At this stage, researchers are also exploring the development of smart biomaterials and devices [248,249]. With ongoing research and technological advancements, more biomaterials and technologies for CVD imaging and detection are expected to emerge in the future to address the needs of clinical diagnosis and treatment.

#### Diabetes mellitus

Diabetes mellitus is a chronic disease characterized by inadequate insulin production, impaired insulin efficacy, or both [250]. The hallmark of this disease is a persistent hyperglycemic state, which can cause long-term damage to various body systems, leading to significant dysfunction [251]. Therefore, regular monitoring of blood glucose levels in patients is essential for developing and implementing effective glycemic control strategies. Diabetes marker assays have become a prominent research topic in substance detection, biomedicine, and health services due to their wide range of applications, which primarily include measurements of blood glucose, insulin, C-peptide, and glycosylated hemoglobin (HbA1c) [252]. Blood glucose and insulin levels indicate short-term glycemic control, whereas C-peptide reflects the functional status of pancreatic β-cells. HbA1c provides an average glucose level over the past 2 to 3 months [253] and is considered one of the gold standards for assessing long-term glycemic control. Blood glucose is typically measured using glucose sensors, insulin is assessed mainly through electrochemical methods, and HbA1c is primarily measured via immunoassays. The most widely used glucose sensors predominantly utilize glucose oxidase (GOD) as a biosensitive substrate and have undergone four generations of technological innovation. In the first two generations, the active centers of GOD are often buried deep within the protein, resulting in low electron transfer efficiency. Although mediators have been employed to enhance electron transfer, their associated costs and complexities pose challenges for widespread clinical application. To address these issues, third-generation glucose sensors facilitate efficient electron transfer between the FAD active group of GOD and the electrode by modifying the electrode surface with functionalized materials, including carbon nanotubes and metal nanomaterials such as Pt-based nanoparticles, Au-based nanoparticles, and metal oxides. For example, Zhou et al. loaded GOD, gold nanoparticles (AuNPs), zinc oxide (ZnO) nanorods, and reduced graphene oxide (rGO) onto an indium tin oxide (ITO) substrate and covered it with Nafion to immobilize GOD and minimize interference from other electroactive species [254]. They employed UV irradiation (302 nm) to generate holes, accelerating the oxidation of GOD (FADH_2_) to GOD (FAD) and thus improving assay sensitivity. Currently, fourth-generation non-enzymatic glucose electrochemical sensors are under development [255]. These sensors utilize nanomaterials, such as carbon nanotubes and metal nanomaterials, that mimic the catalytic activity of enzymes to directly catalyze glucose oxidation, thereby avoiding the complexities and instabilities associated with enzymes. Additionally, research on non-enzymatic glucose sensors includes surface modification and functionalization of nanomaterials to enhance their selective catalytic activity for glucose. These developments offer promising new strategies for glucose monitoring in diabetes management, aiming to enable rapid, accurate, and cost-effective glucose detection [256].

#### Retinopathy

Retinopathy is one of the leading causes of vision impairment, particularly DR. DR is a significant microvascular complication of diabetes mellitus that can quickly progress to vision-threatening DR (VTDR), ultimately leading to irreversible visual impairment [257]. Early systematic screening and prompt treatment have proven highly effective in preventing visual loss. With advances in biomedical imaging techniques, retinal imaging has become an essential tool for assessing and diagnosing retinopathy. Testing for DR primarily involves analyzing various biological fluids obtained from patients as potential biomarkers [258], which can include vitreous fluid, atrial fluid, and blood. Biomarkers revealed by retinal imaging techniques vary. For instance, fundus color photography and fluorescein fundus angiography primarily visualize microaneurysms, hemorrhages, and exudates [259], whereas OCT focuses on retinal thickness and structural changes in the macula [260]. Blood tests and analysis of atrial fluid samples measure VEGF and other inflammatory factors [261]. However, conventional imaging methods for retinopathy often rely on contrast agents, which may trigger allergic reactions. Some biomaterials exhibit good biocompatibility, thereby reducing adverse reactions associated with retinal imaging and yielding clearer imaging results. For example, Coro et al. prepared Ag2S nanoparticles that demonstrated excellent biocompatibility [262]. These nanoparticles were used not only as an OCT contrast agent but also as a NIR ocular imaging probe, showcasing outstanding imaging characteristics. Notably, OCT angiography (OCTA) is a groundbreaking technique that belongs to a form of functional OCT. It has the advantage of eliminating the need for contrast agents by utilizing red blood cells as a natural contrast medium to depict the vascular structure. This is achieved by detecting the movement of red blood cells, which allows for the visualization of the vascular system within different retinal and choroidal layers [263]. Currently, the application of biomaterials in the field of OCTA is still in its early stages. However, OCTA leverages the principle of low-coherence optical interference to capture backscattered signals. Biomaterials, such as nanoparticles, can serve as enhancers, increasing the scattering signal to improve the contrast of the OCTA images and reveal the retinal vasculature in greater detail. Additionally, biomaterials can enhance the design of OCTA probes, making them gentler on ocular tissues and reducing patient discomfort during examination.

#### Alzheimer's disease

**Fig. 9:**
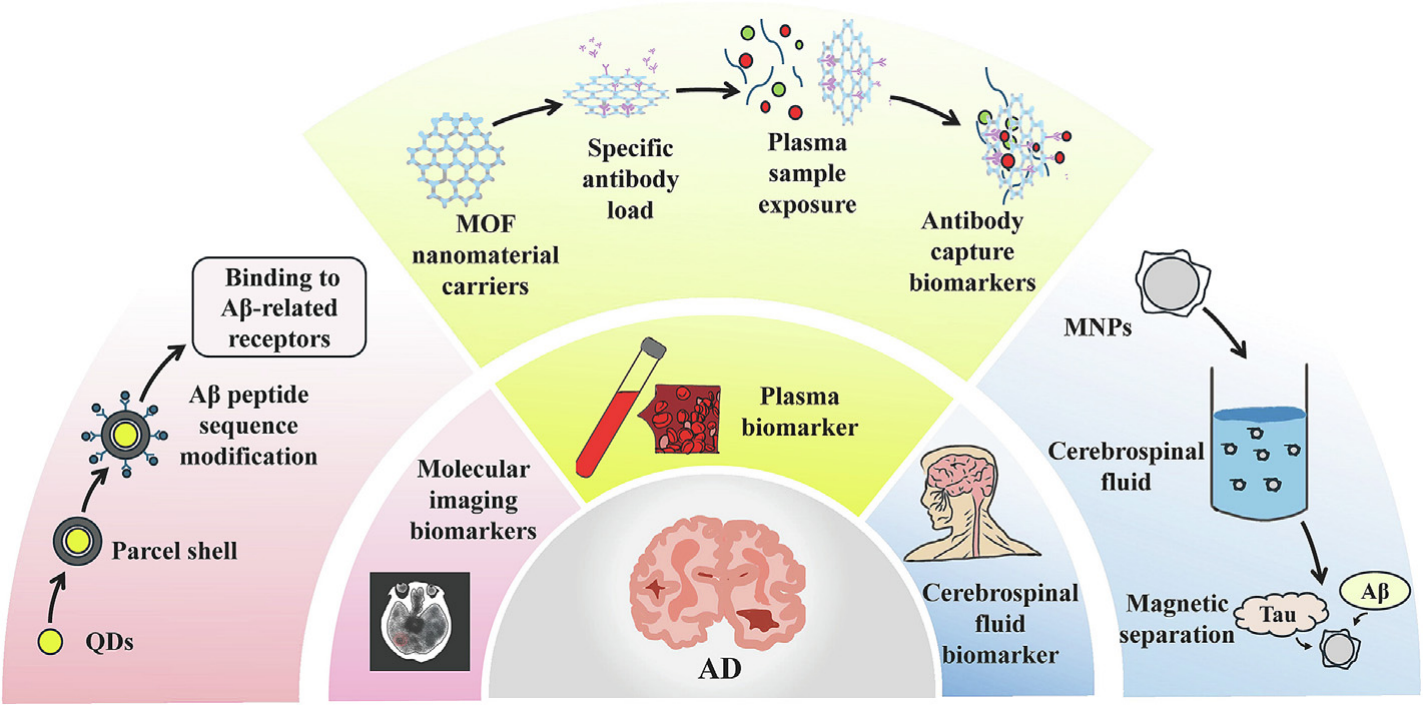
Schematic illustration of the application of biomaterial nanotechnology in the detection of Alzheimer's disease biomarkers.

AD is a chronic neurodegenerative condition characterized by progressively worsening cognitive dysfunction, ultimately leading to severe loss of mental function. The pathological basis of the disease involves a progressive reduction of nerve cells in the brain, abnormal accumulation of β-amyloid (Aβ) that forms senile plaques, and the tangling of neurofibrillary fibers due to the abnormal phosphorylation of tau proteins [264] (Fig. 9). These pathological changes are particularly pronounced in specific regions of the brain, making biomarker testing of CSF samples for Aβ peptides (especially Aβ1-42 and Aβ1-40), total tau protein, and phosphorylated tau (p-tau181) critical for the clinical diagnosis of AD [265]. In addition to these traditional biomarkers, amyloid-derived diffusible ligands and proteins that impact tau protein imbalance have also been recognized as potential markers for AD diagnosis [266,267]. The primary diagnostic techniques for AD include ELISA and microsphere suspension dot-matrix techniques [268,269]. Both methods utilize antibodies as the recognition and capture elements for biomarkers; however, they have limitations concerning timeliness and accuracy. To address these limitations, researchers are investigating the use of newly developed biomimetic receptors instead of traditional antibodies. These include nucleic acid aptamers, peptide receptors, peptide-like receptors, MIPs, nanobodies, and others. Notably, peptide and peptidomimetic receptors are increasingly used in actual Alzheimer’s detection, while nucleic acid aptamers and MIPs, though less frequently applied, show great promise. For example, Jia et al. developed a CRISPR-driven aptamer sensor to quantify Aβ40 and Aβ42 biomarkers in CSF. This approach combines the high specificity of Aβ biomarker aptamers with the fluorescence detection capabilities of the CRISPR-Cas12a system, allowing for the assay to be completed in less than 60 min with high sensitivity [270].

In addition, blood marker assays represent an early detection method with significant potential for AD. These assays offer various advantages, such as easy sampling, being less invasive, and demonstrating reproducibility compared to CSF marker assays [271]. Furthermore, they show a significant correlation with biomarker levels in CSF. The main techniques currently used for blood marker detection include electrochemiluminescence, chemiluminescence, immunomagnetic subtraction, single molecule arrays, and immunoprecipitation-mass spectrometry. Among these methods, nanomaterials—such as metallic nanoparticles, MNPs, and QDs—are employed to construct highly sensitive biosensors for the detection of AD biomarkers in blood samples. These nanomaterials can enhance detection signals and improve the identification of low-abundance biomarkers [272]. For instance, Liu et al. developed an electrochemical DNA biosensor based on polyamide/polyaniline carbon nanotubes (PA/PANI-CNT) for the rapid detection of Aβ42 in human blood, which is crucial for the early diagnosis of AD [273]. It is hypothesized that future nanoparticles may integrate diagnostic and therapeutic functions, providing more effective treatment options for AD. As clinical trials progress, nanotechnology-based AD assays are expected to become part of standard clinical practice.

## Summary and outlook

As an important tool for disease diagnosis, prognosis assessment and efficacy monitoring, biomarkers play a crucial role in the development of individualized medicine. From the perspective of sustainable development, the application of biomarkers reduces the reliance on traditional invasive detection methods, decreases the consumption of medical resources and the physical damage to patients, and is in line with the requirements of sustainability. In the field of green chemistry, it helps with the monitoring of environmental pollutants, promotes the safety assessment and management of chemical substances, and drives the chemical industry towards a greener and more environmentally friendly direction. In recent years, the innovative application of biomaterials in biomarker detection is promoting the diagnosis of diseases from “macro-grouping” to “micro-precision”. New materials such as nanoparticles, QDs and biopolymers have broken through the limitations of traditional immunoassay and mass spectrometry technologies by virtue of their ultra-high sensitivity, multimodal signal output and dynamic response. In the fields of cancer and CVD, biomaterials-driven liquid biopsy technology has achieved single-molecule detection of circulating tumor DNA, while QD-labeled multiphoton imaging technology has improved the resolution of neuronal synapses to 200 nm, providing a new tool for early diagnosis of AD. Current technological bottlenecks are centered on three core contradictions: the imbalance between sensitivity and specificity, the contradiction between imaging depth and resolution, and the disconnect between laboratory performance and clinical stability. First, the imbalance between sensitivity and specificity is prominent. For example, although the SPR effect of nanomaterials can significantly enhance the Raman signals, the non-specific adsorption phenomenon leads to an increase in the false-positive rate, which directly affects the reliability of the detection results. Secondly, it is difficult to balance the imaging depth and resolution. Taking two-photon imaging technology as an example, although it can realize tissue observation at a depth of 500 μm, the resolution is still limited to 1 μm level, which cannot meet the clinical needs of higher precision. Finally, the disconnection between laboratory performance and clinical stability is particularly serious, directly affecting the long-term stability and practicality of the equipment. These issues show that the existing technology in the breakthrough performance limits, but still need to address the stability and reliability challenges in practical applications.

Future technological development will focus on the functional integration of imaging and detection and the reconstruction of clinical utility. On the one hand, the intelligent design of stimulus-responsive probes has become a key breakthrough, for example, ROS/pH dual-activated QDs can dynamically release targeted ligands in the TME, synchronously achieving in situ marker enrichment and near-infrared two-region fluorescence imaging, so that detection sensitivity and spatial localization accuracy form a synergistic enhancement. On the other hand, multimodal technologies are breaking the limitations of single methods, such as the integration of PIA and surface-enhanced spectroscopy, which can locate breast cancer foci with a resolution of 50 μm, and also realize molecular typing by analyzing the vibrational spectra of HER2 receptor, which provides a brand new solution for “image-guided biopsy”. In future research, we propose the following optimizations: First, precisely select materials according to the properties of biomarkers. Second, optimize the physical and chemical properties of the materials to meet specific requirements. Third, ensure good biocompatibility of the materials. Fourth, enhance the stability of the materials in different environments. Fifth, further improve the imaging and detection performance of the materials. Sixth, design them in combination with actual application scenarios. We aim to promote the innovative development of biomarker detection and imaging materials through the interdisciplinary integration of materials science and biotechnology, helping the biomedical field reach new heights. At the clinical translation level, the innovation of whole blood direct detection technology significantly shortens the diagnostic process, which is an important research direction in the future. With the rapid analysis of Raman spectra by deep learning algorithms and the maturation of biomimetic interface design to inhibit biological contamination and other auxiliary technologies, biomaterials-driven testing systems are moving from the laboratory to the clinical site. It is expected that individualized disease models constructed by organ chips will drive the testing technology to “in vivo real-time monitoring” in the future, and the combination of the Material Genome Project and high-throughput screening platform is expected to further shorten the research and development cycle of new biomaterials. In addition, by solving industrial-level problems such as biosafety and manufacturing consistency, biomarker imaging and detection technologies will realize large-scale applications in early cancer diagnosis, dynamic assessment of neurodegenerative diseases and other scenarios, and ultimately promote the substantive breakthrough of precision medicine from data accumulation to clinical intervention.

## Compliance with ethics requirements

*This article does not contain any studies with human or animal subjects.*

## CRediT authorship contribution statement

**Yan Wang:** Writing – original draft, Writing – review & editing, Formal analysis, Conceptualization, Methodology. **Xinyu Huang:** Investigation, Formal analysis, Conceptualization, Validation. **Guiying Wu:** Methodology, Validation, Formal analysis. **Wanping Wu:** Validation, Formal analysis, Conceptualization. **Shuang Li:** Formal analysis, Validation. **Chunyu Su:** Conceptualization. **Li Li:** Project administration, Supervision, Resources, Conceptualization. **Qizhuang Lv:** Project administration, Funding acquisition, Supervision.

## Declaration of competing interest

The authors declare that they have no known competing financial interests or personal relationships that could have appeared to influence the work reported in this paper.

## Data Availability

No data was used for the research described in the article.
