# Supplementary figures and images for: Biomaterials for biomarker imaging and detection
Source: J Adv Res. 2025 Aug 7;83:219–51. doi: 10.1016/j.jare.2025.07.049 (PMC13131447; doi:10.1016/j.jare.2025.07.049)

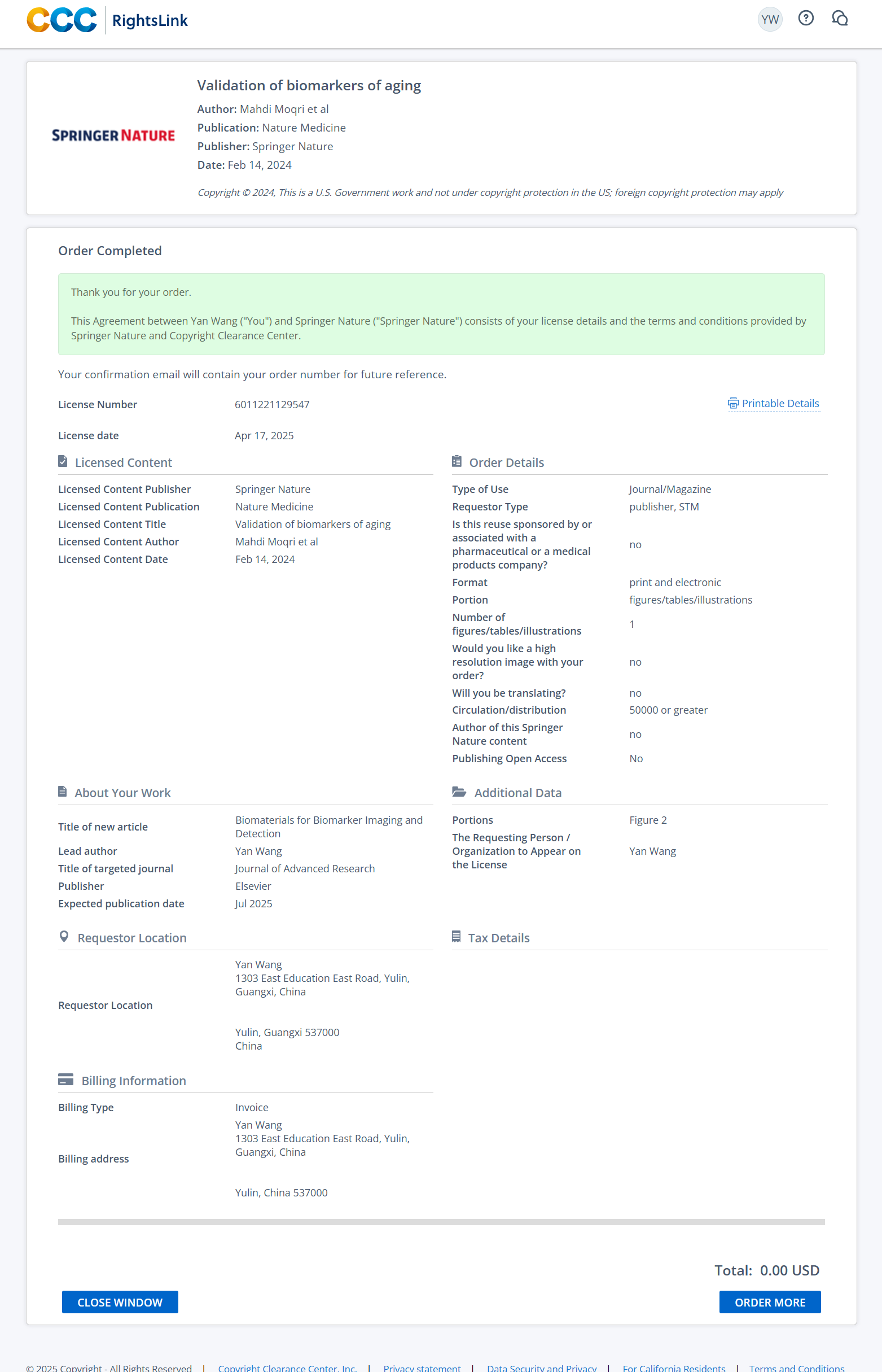

Supplement: Supplementary Data 2 [file mmc2.docx]

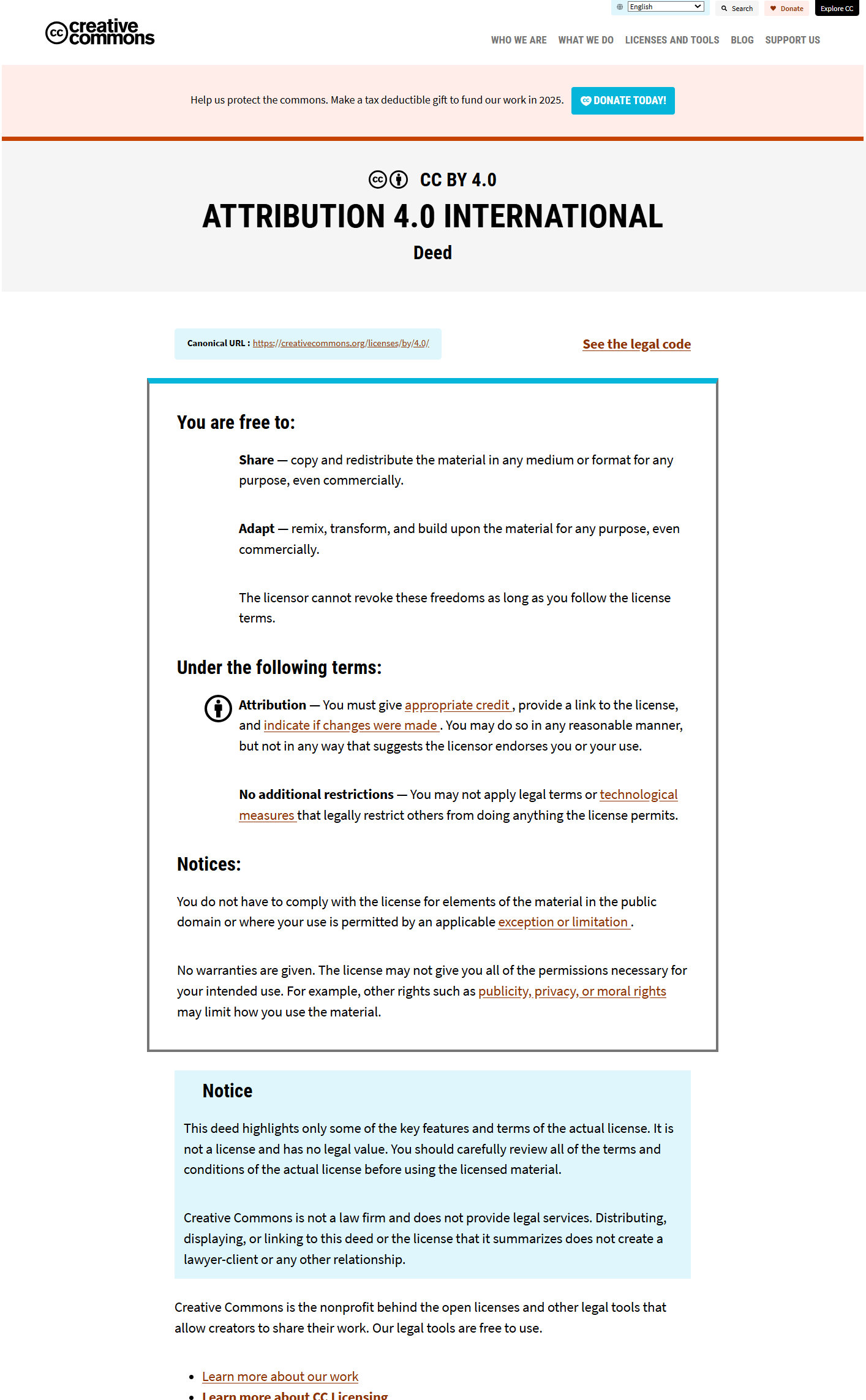

Supplement: Supplementary Data 3 [file mmc3.docx]

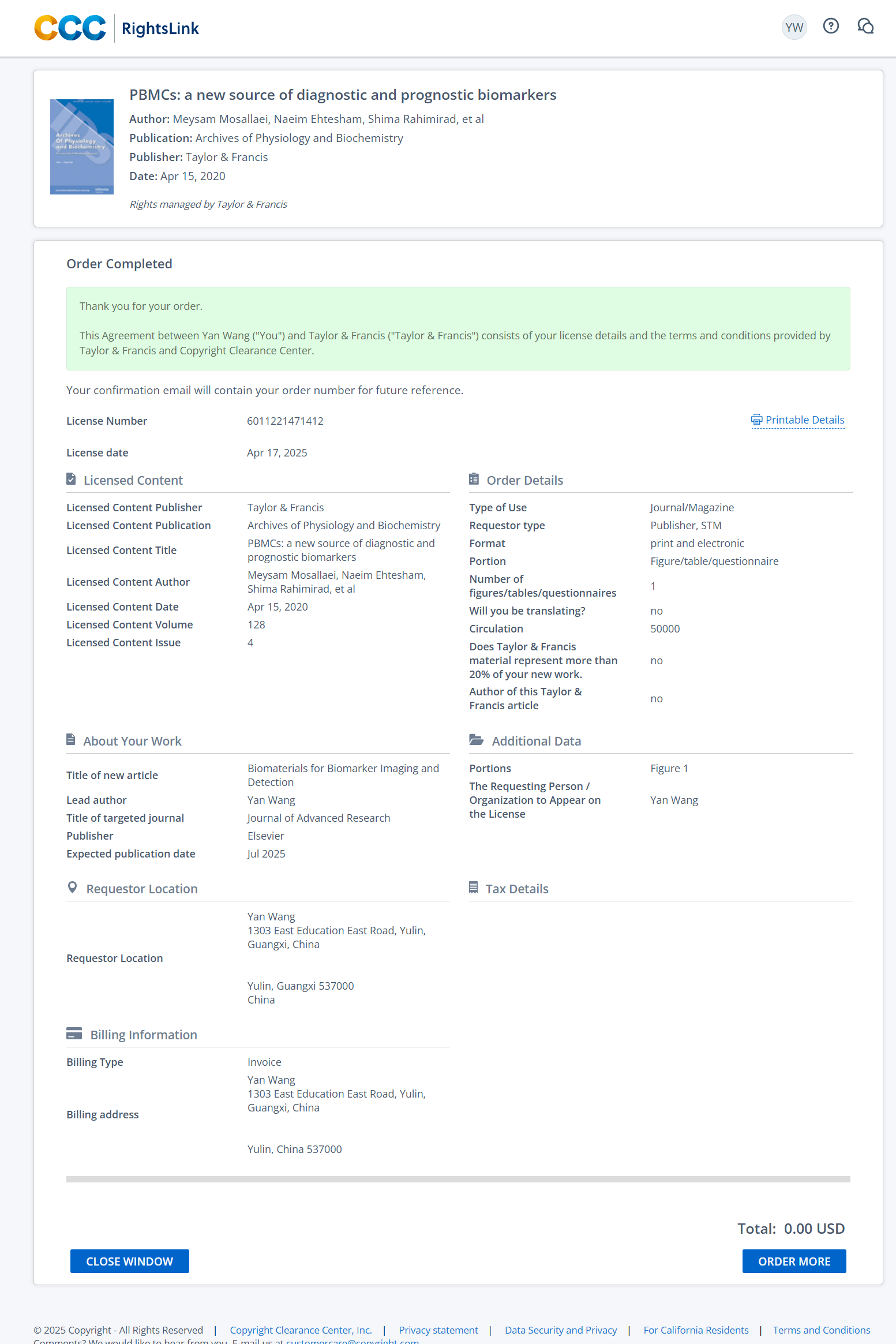

Supplement: Supplementary Data 4 [file mmc4.docx]

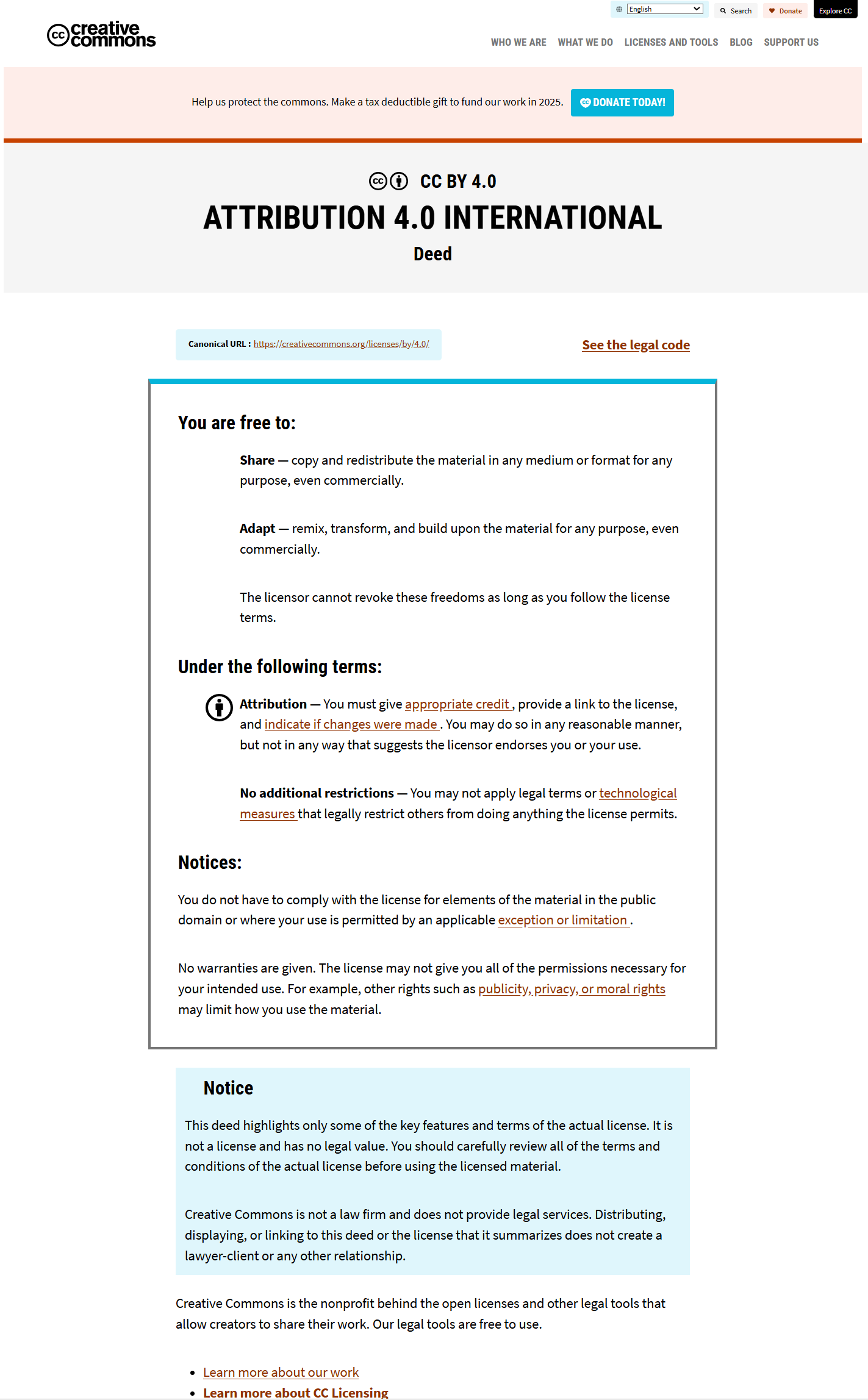

Supplement: Supplementary Data 5 [file mmc5.docx]

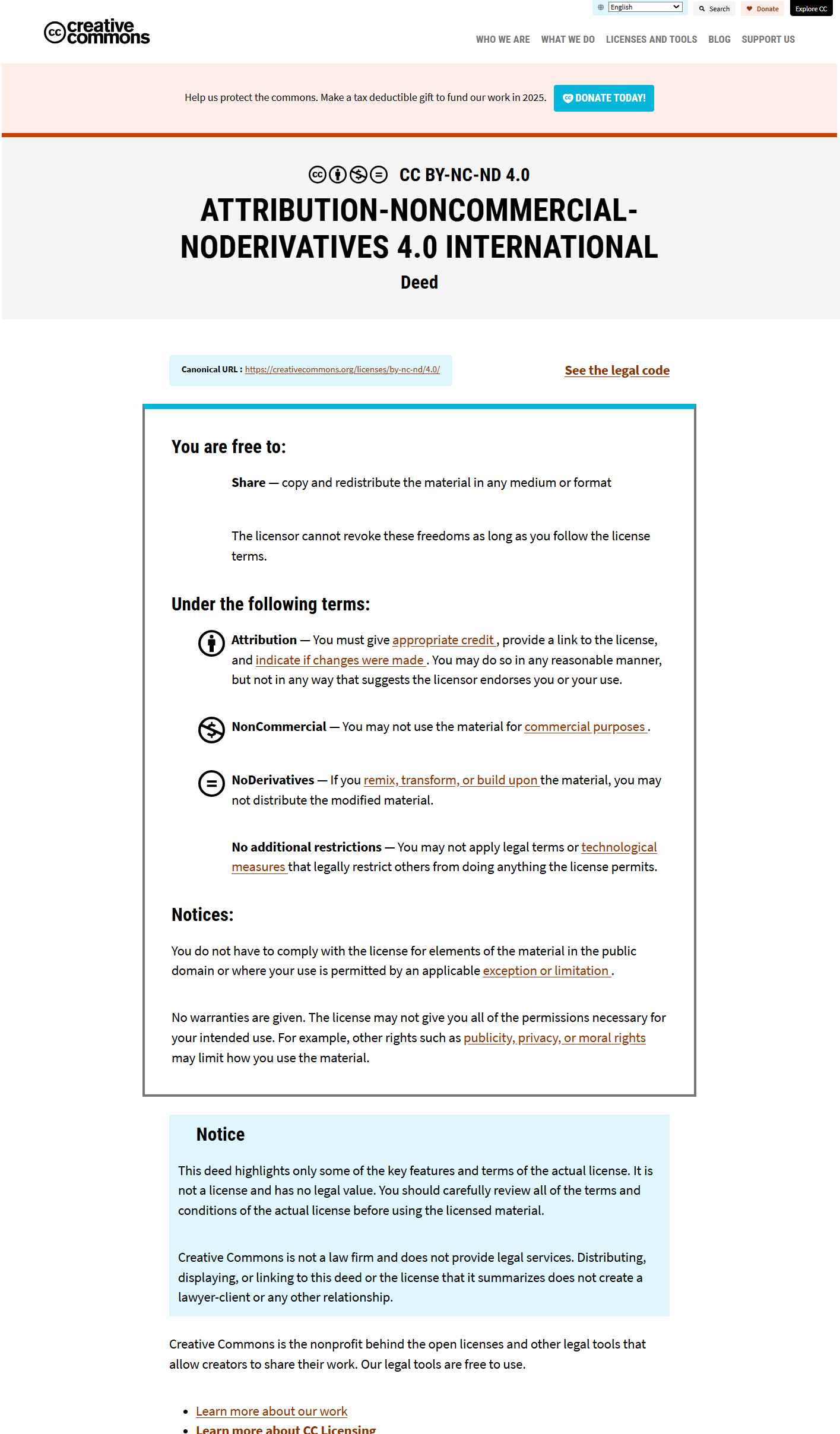

Supplement: Supplementary Data 6 [file mmc6.docx]

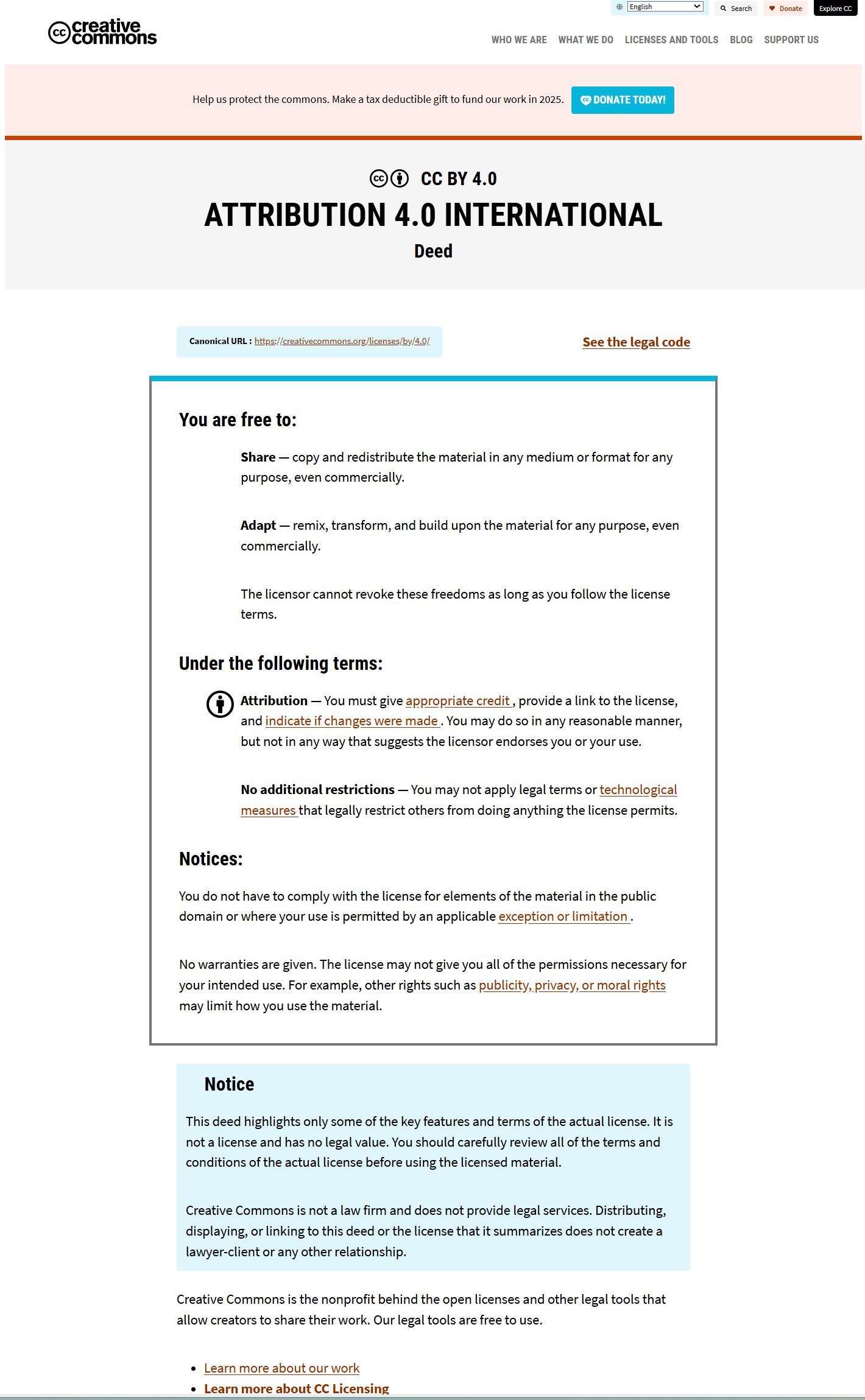

Supplement: Supplementary Data 7 [file mmc7.docx]

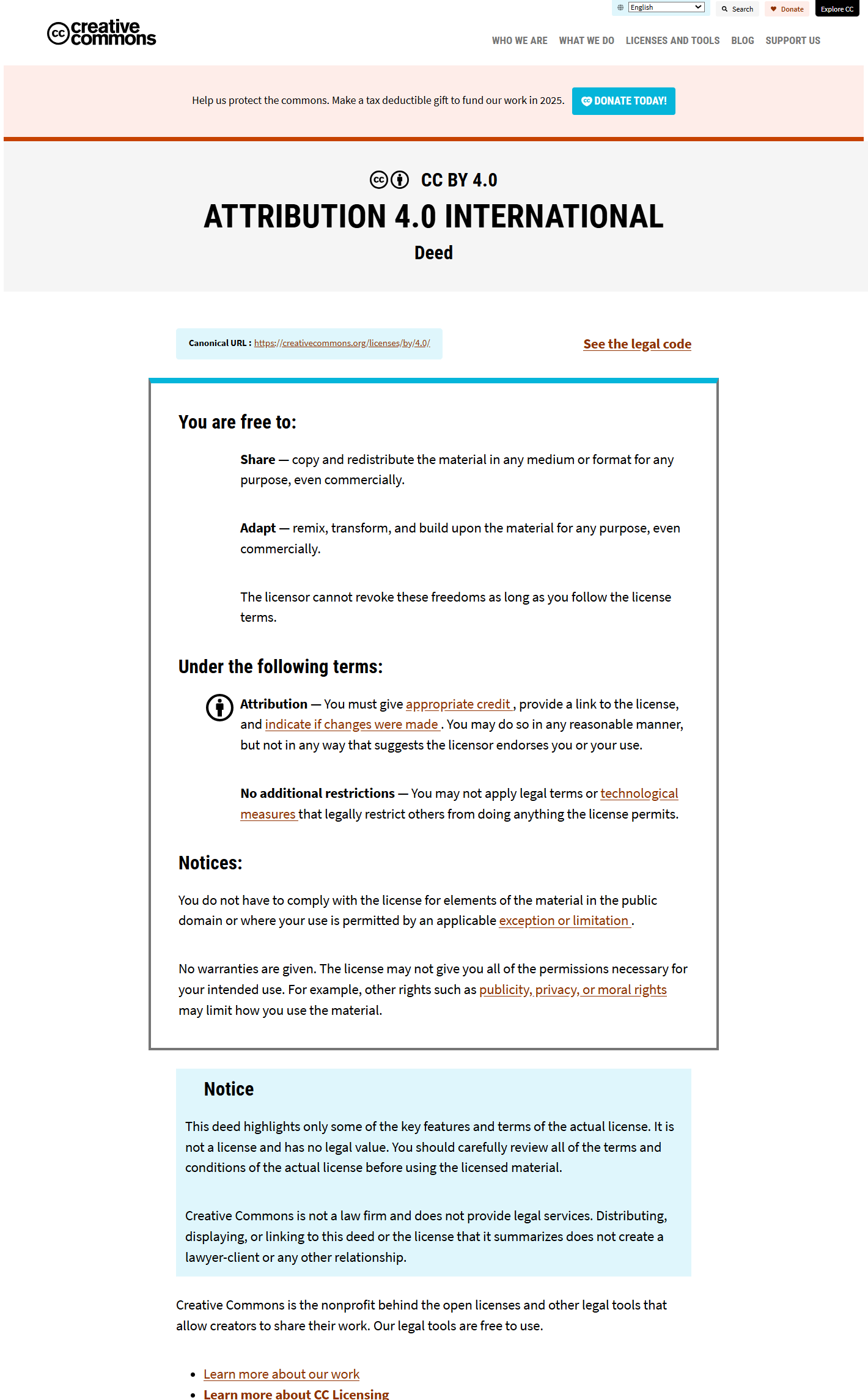

Supplement: Supplementary Data 9 [file mmc9.docx]

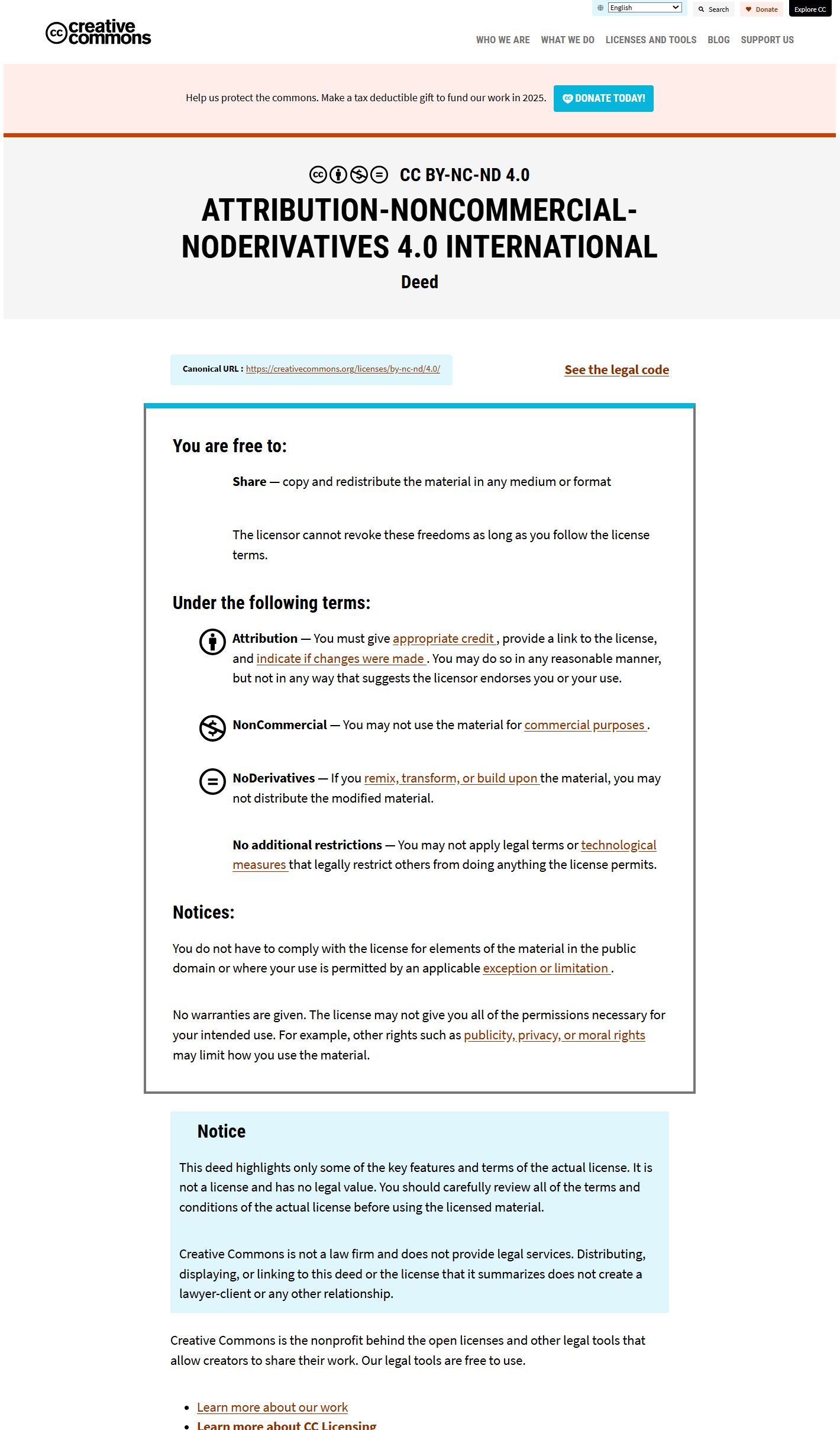

Supplement: Supplementary Data 10 [file mmc10.docx]

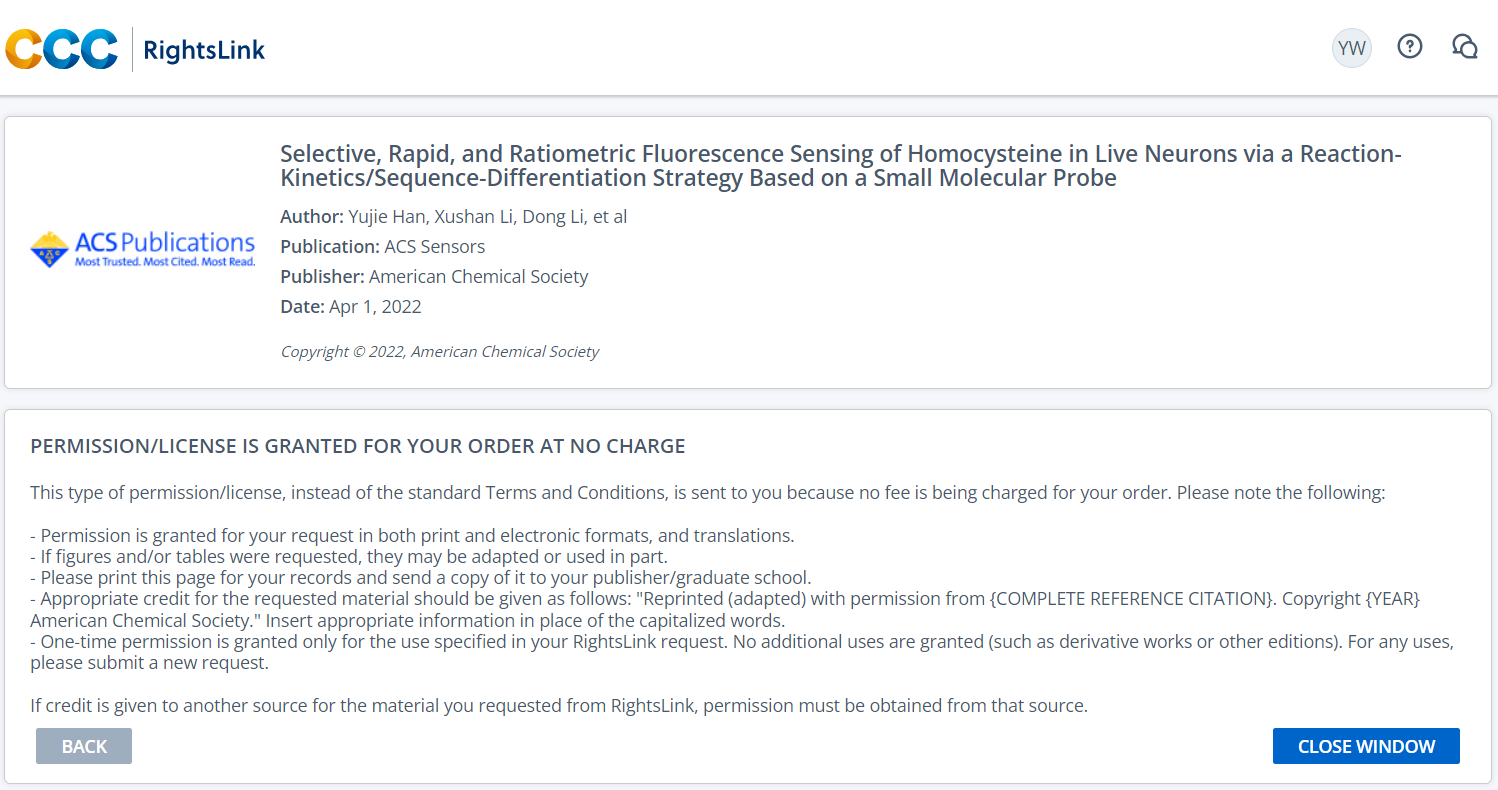

Supplement: Supplementary Data 12 [file mmc12.docx]

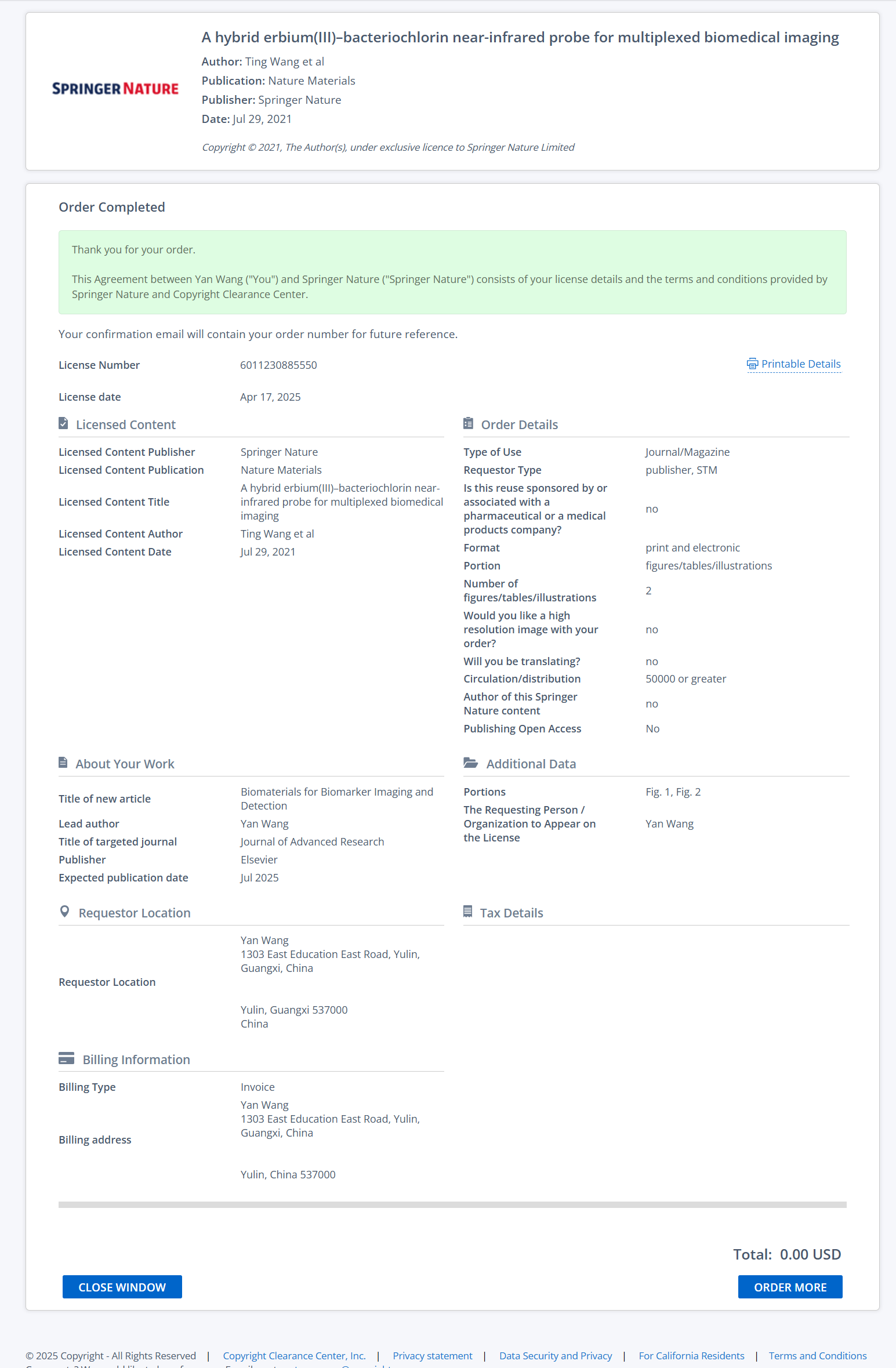

Supplement: Supplementary Data 13 [file mmc13.docx]

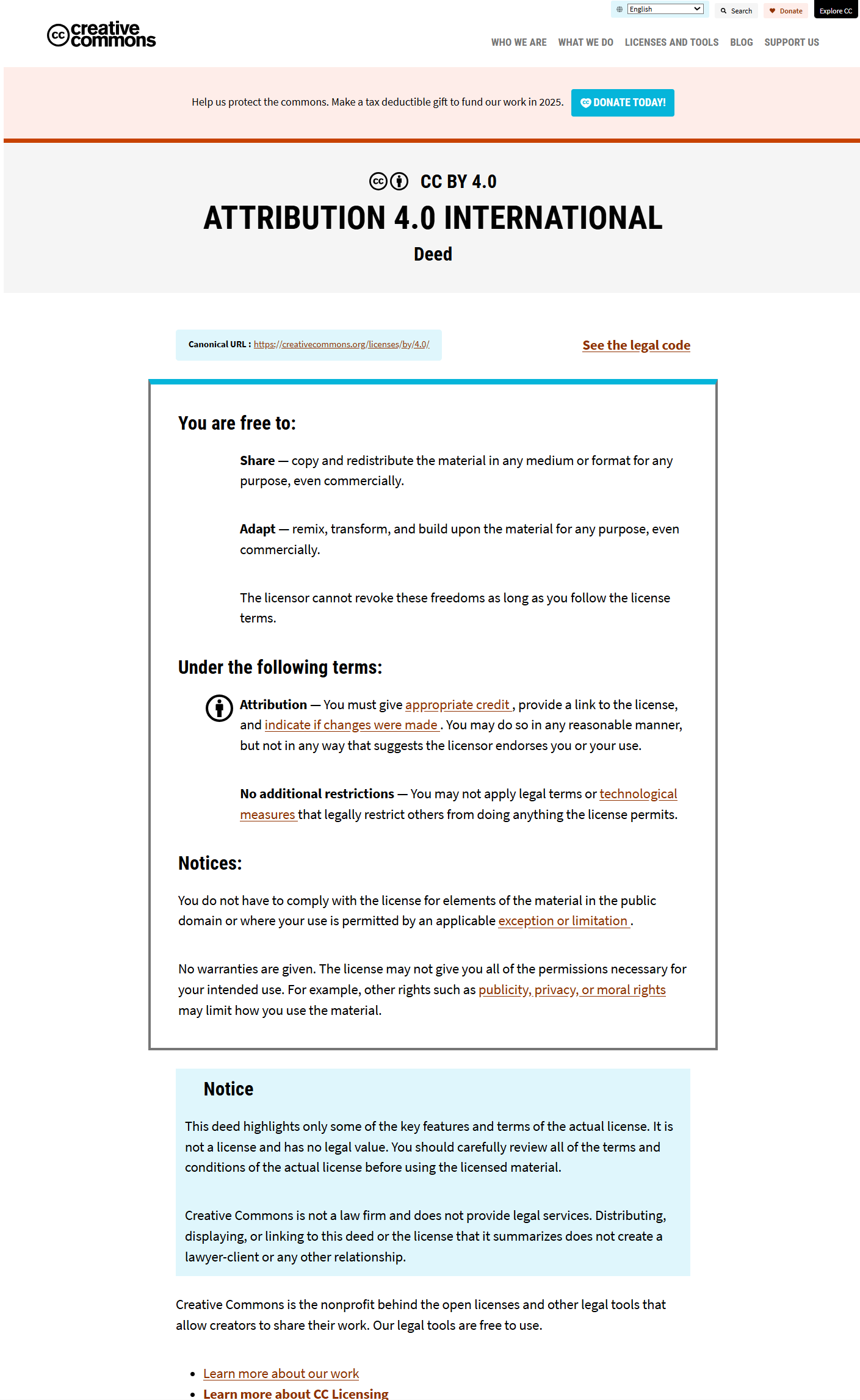

Supplement: Supplementary Data 14 [file mmc14.docx]

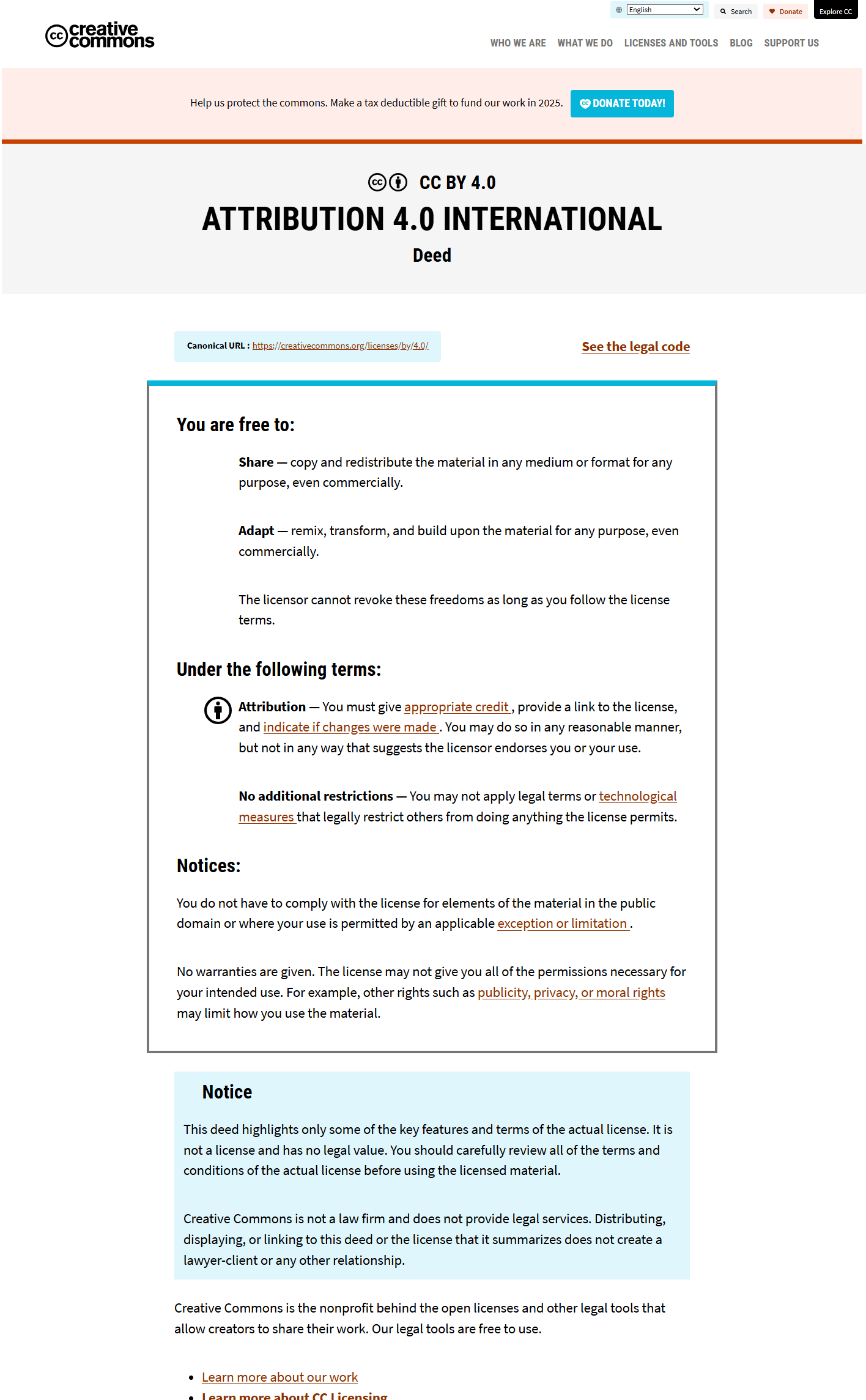

Supplement: Supplementary Data 15 [file mmc15.docx]

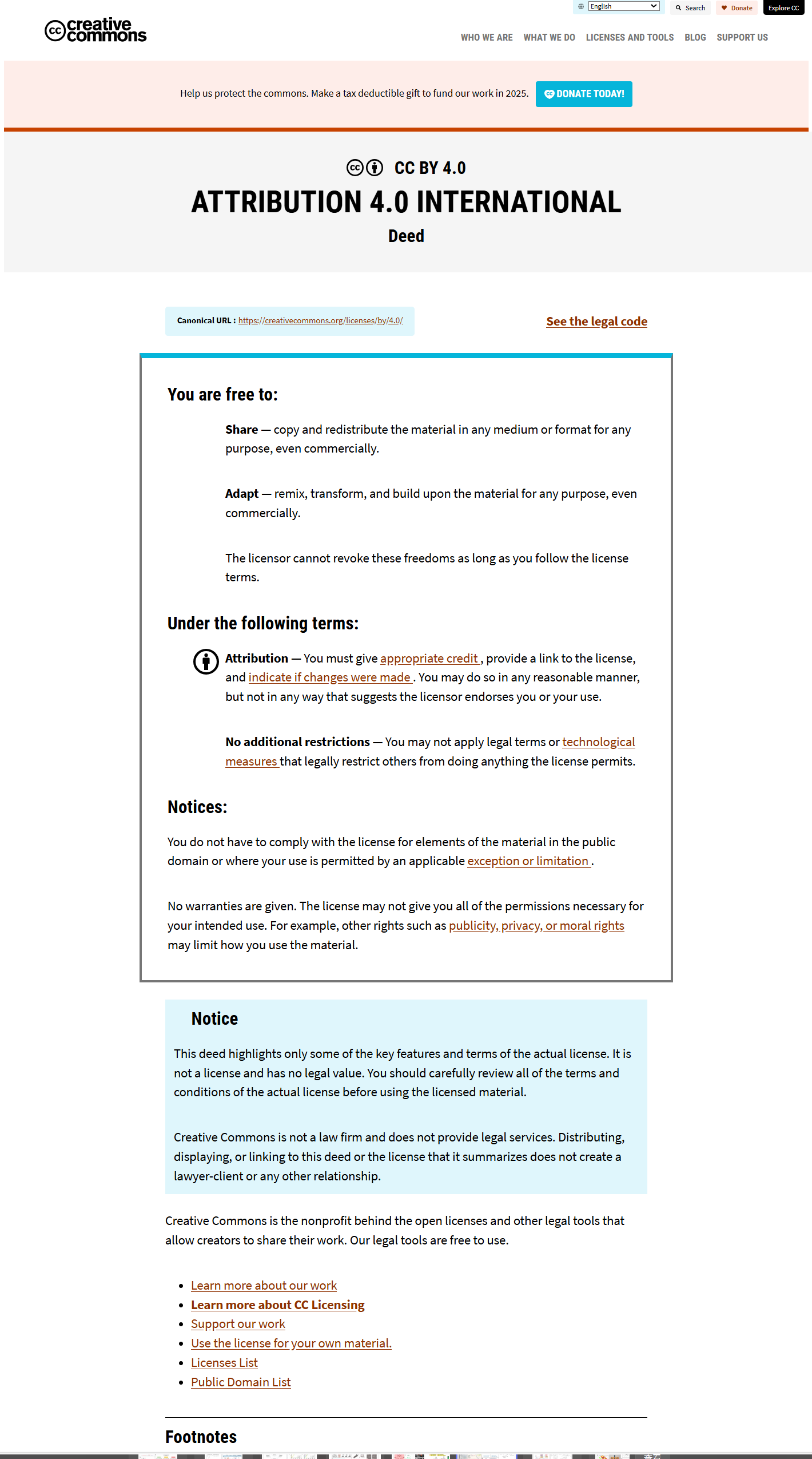

Supplement: Supplementary Data 16 [file mmc16.docx]

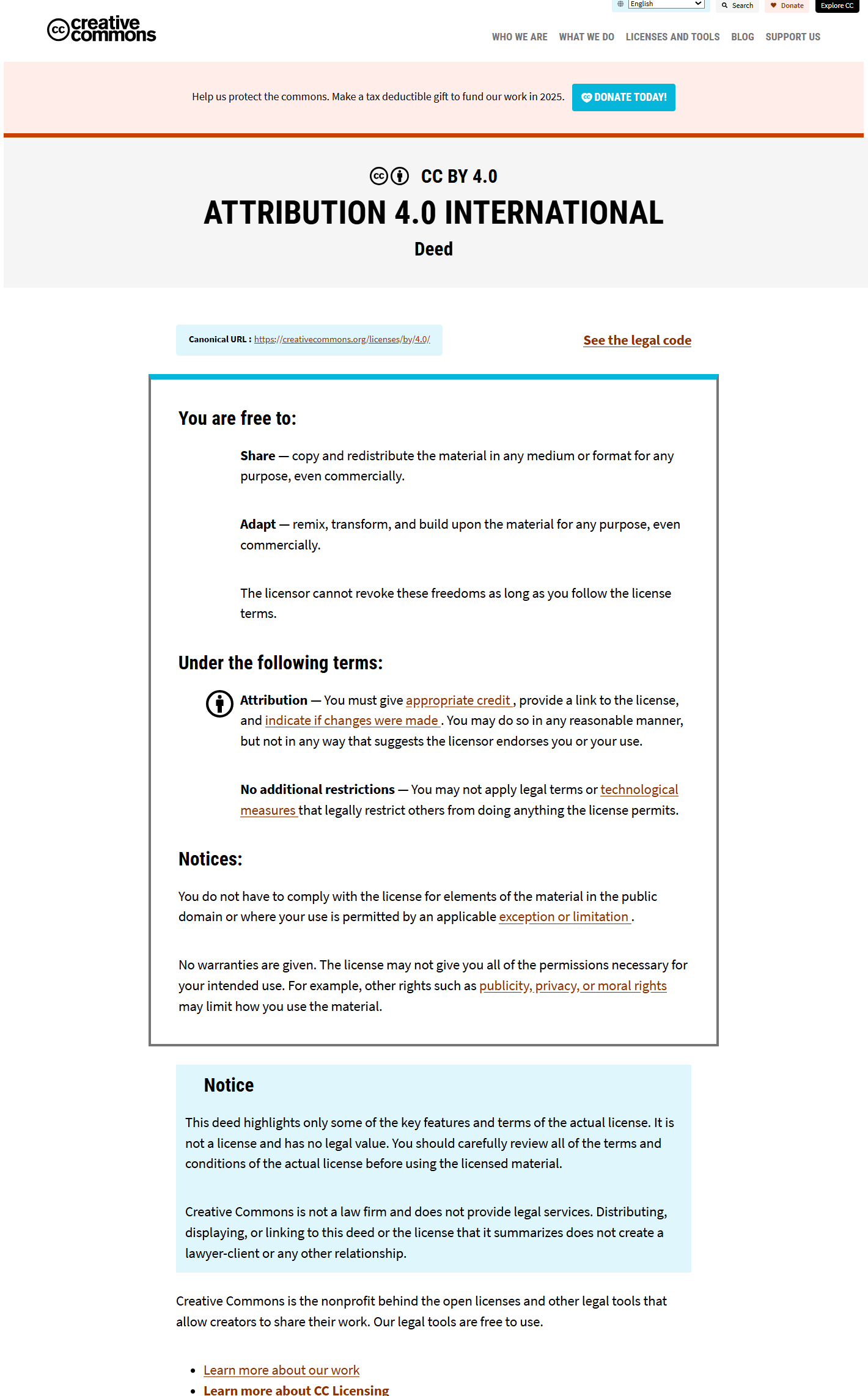

Supplement: Supplementary Data 17 [file mmc17.docx]

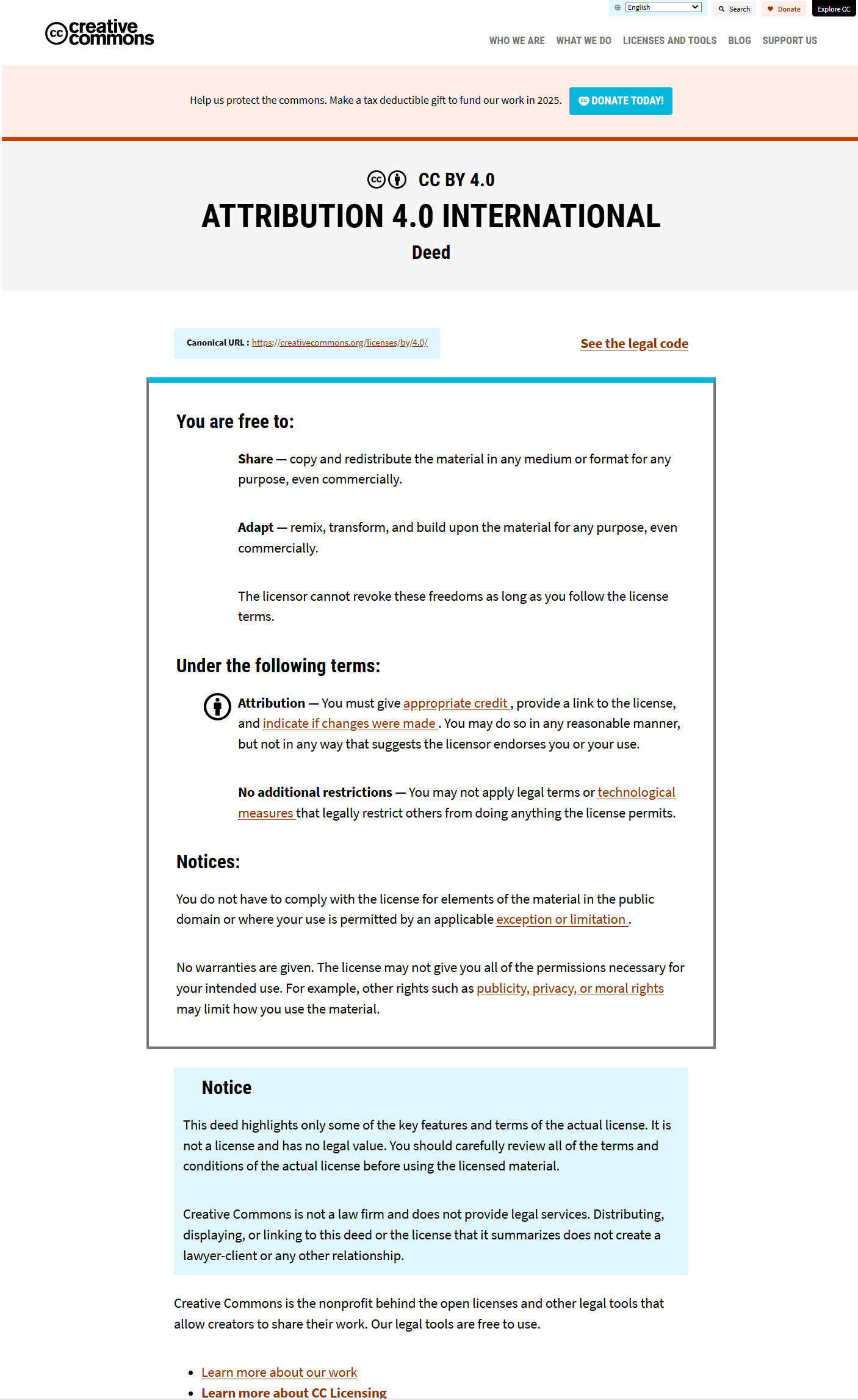

Supplement: Supplementary Data 18 [file mmc18.docx]

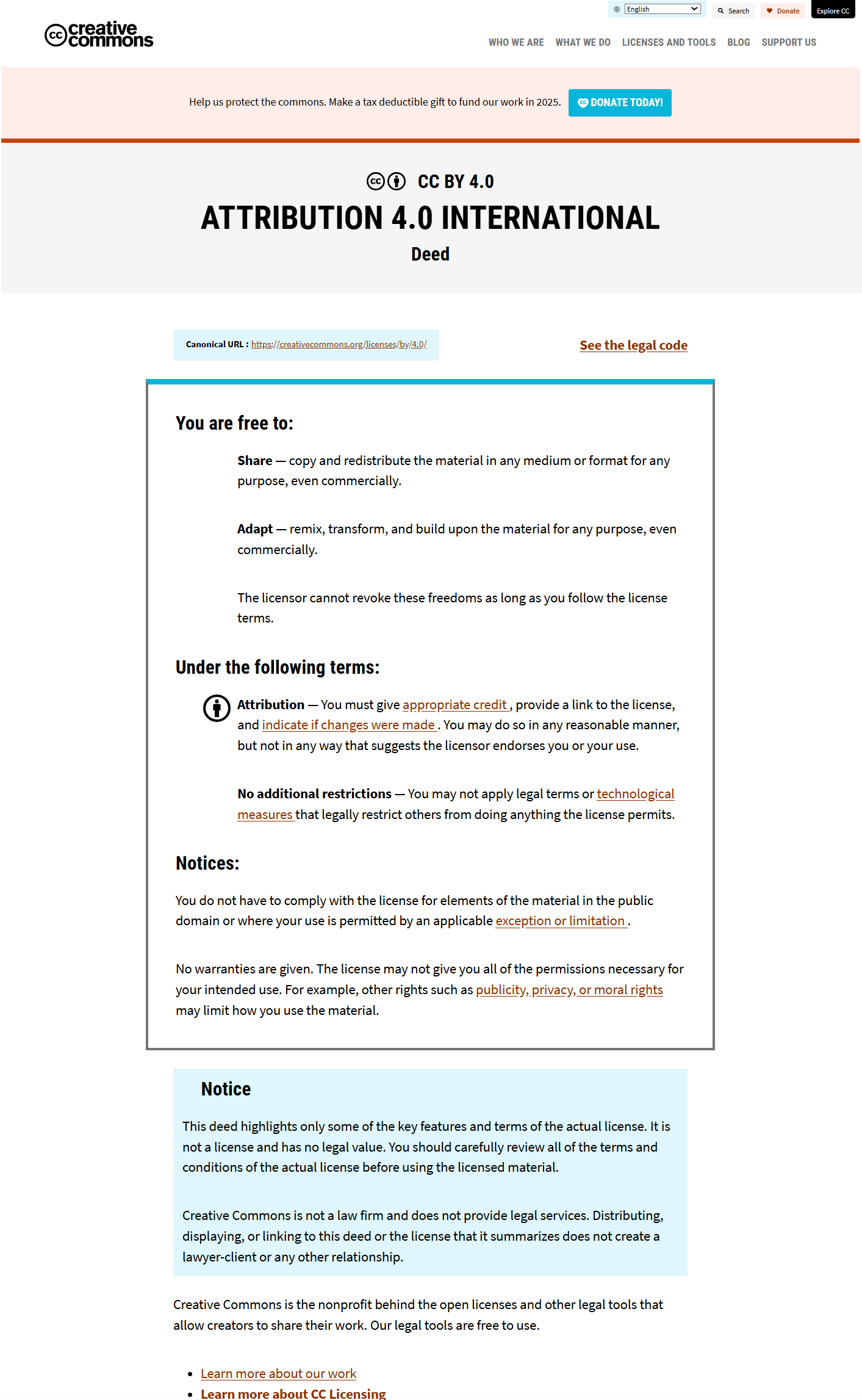

Supplement: Supplementary Data 19 [file mmc19.docx]

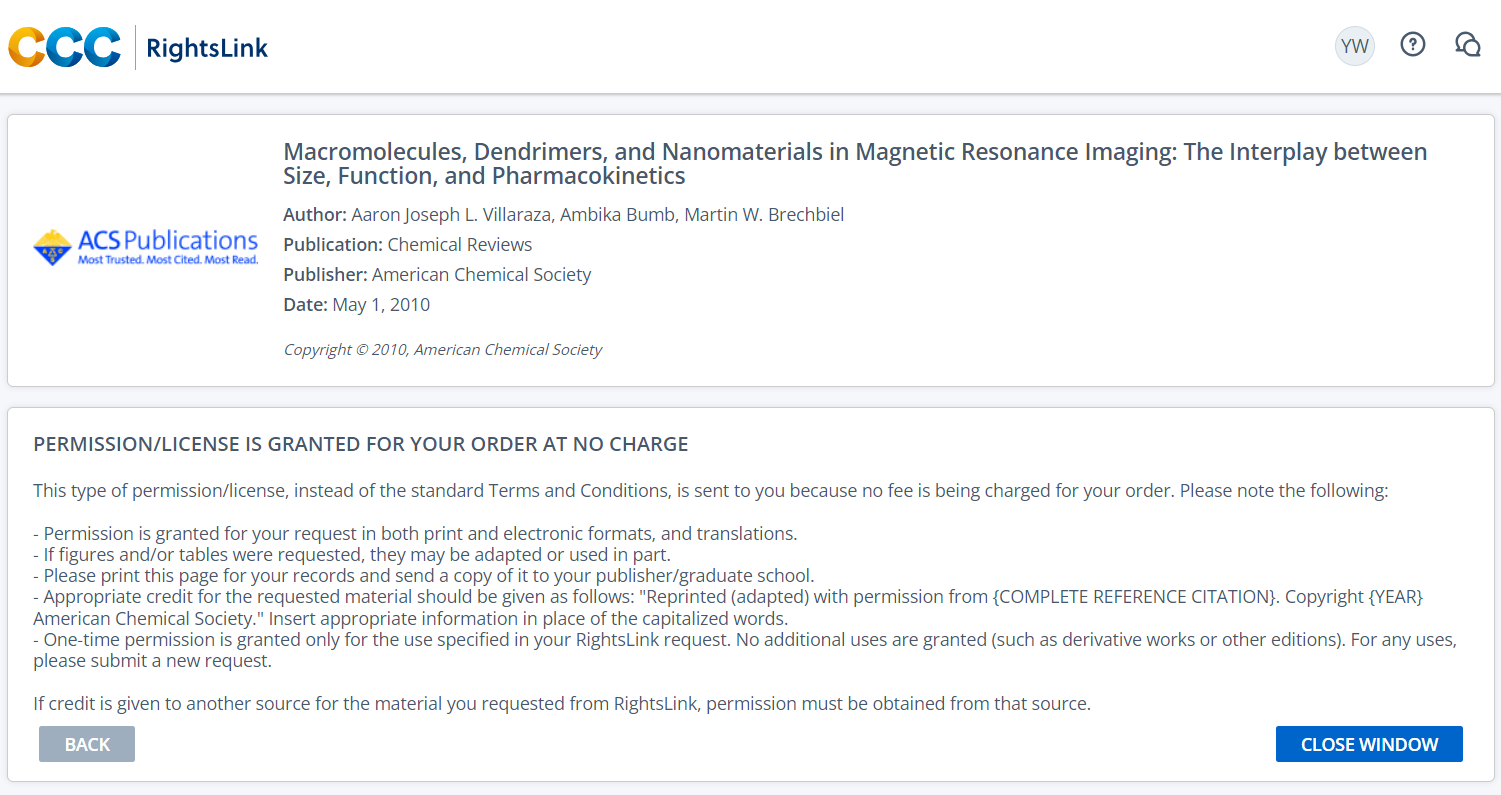

Supplement: Supplementary Data 20 [file mmc20.docx]

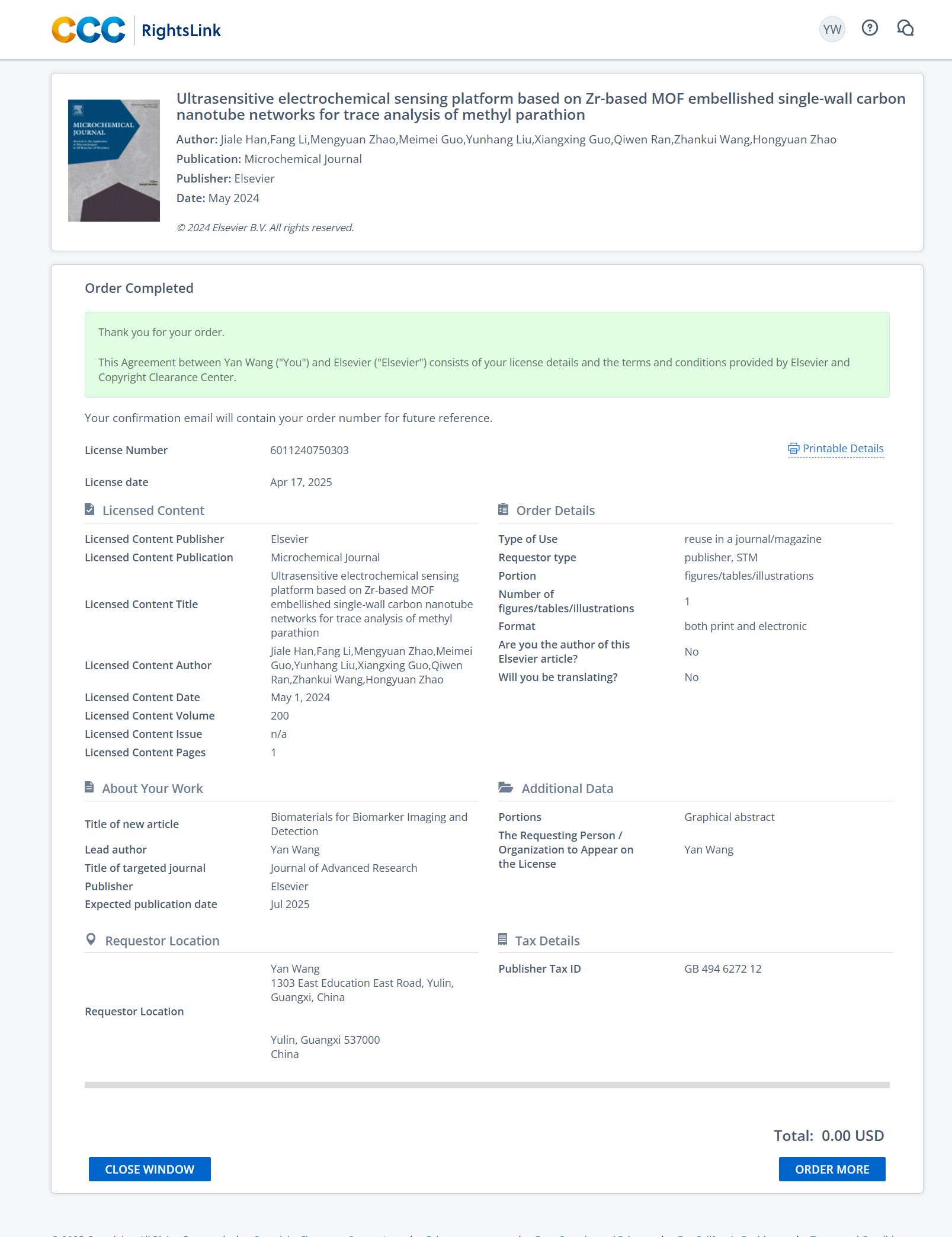

Supplement: Supplementary Data 21 [file mmc21.docx]

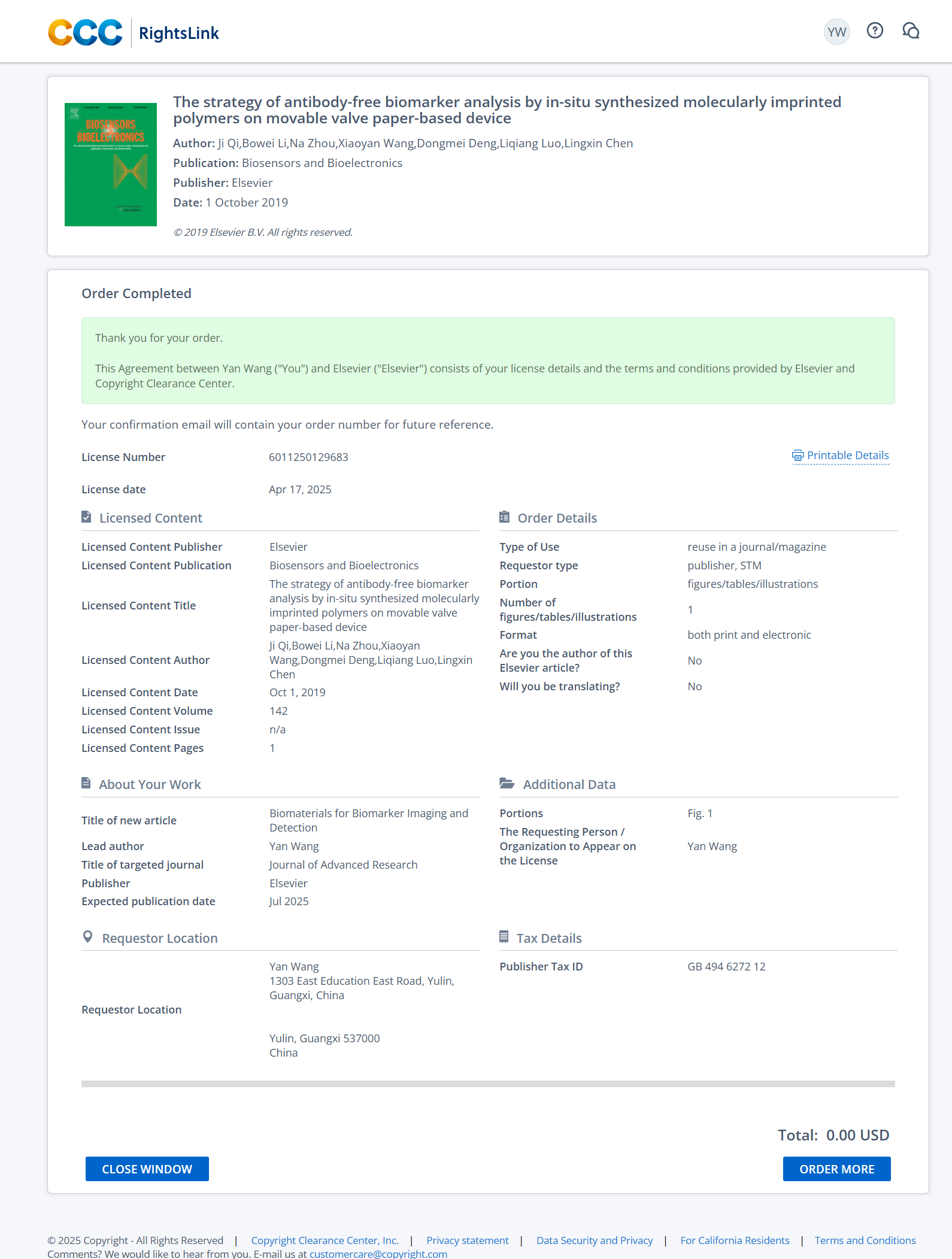

Supplement: Supplementary Data 22 [file mmc22.docx]

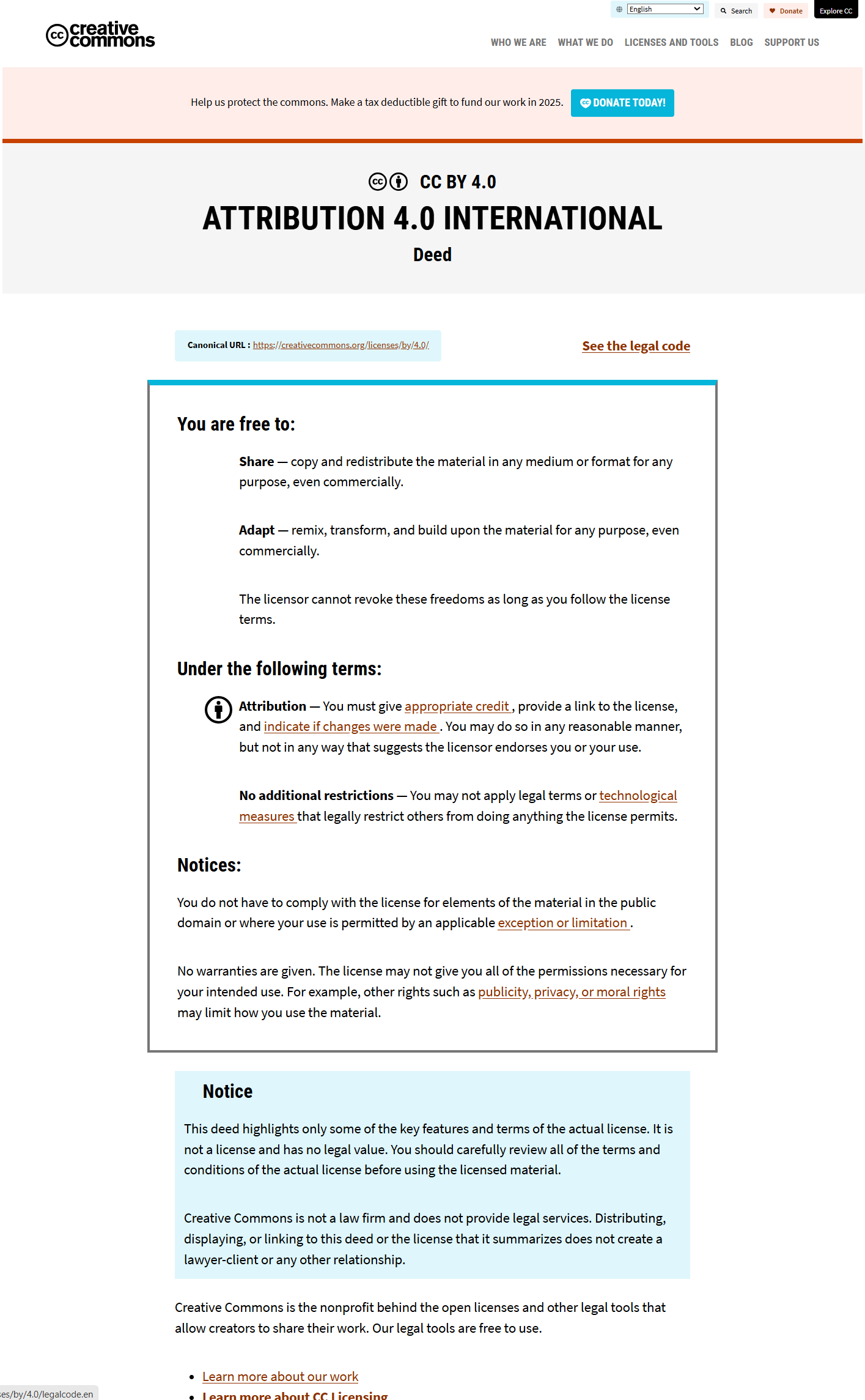

Supplement: Supplementary Data 23 [file mmc23.docx]

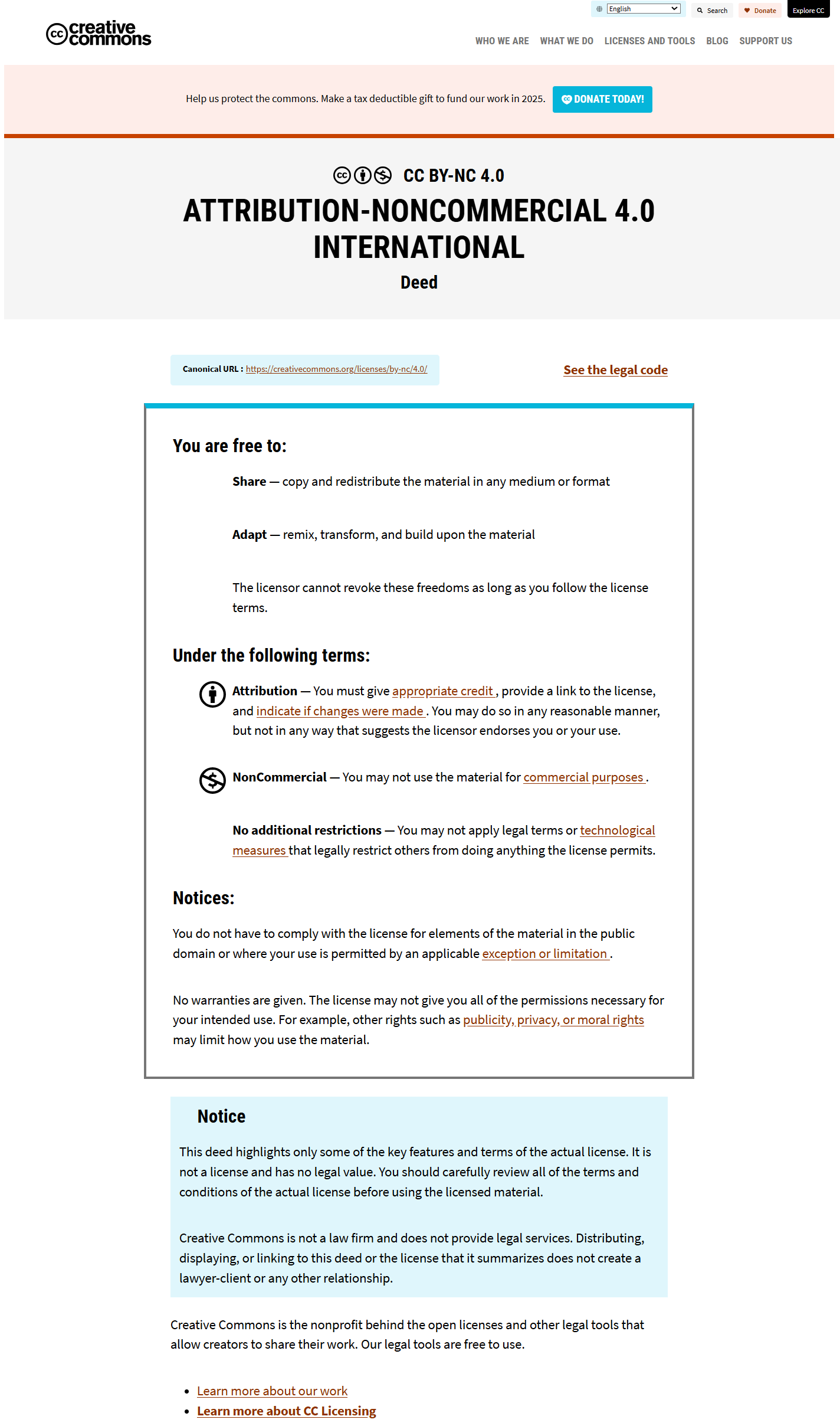

Supplement: Supplementary Data 24 [file mmc24.docx]

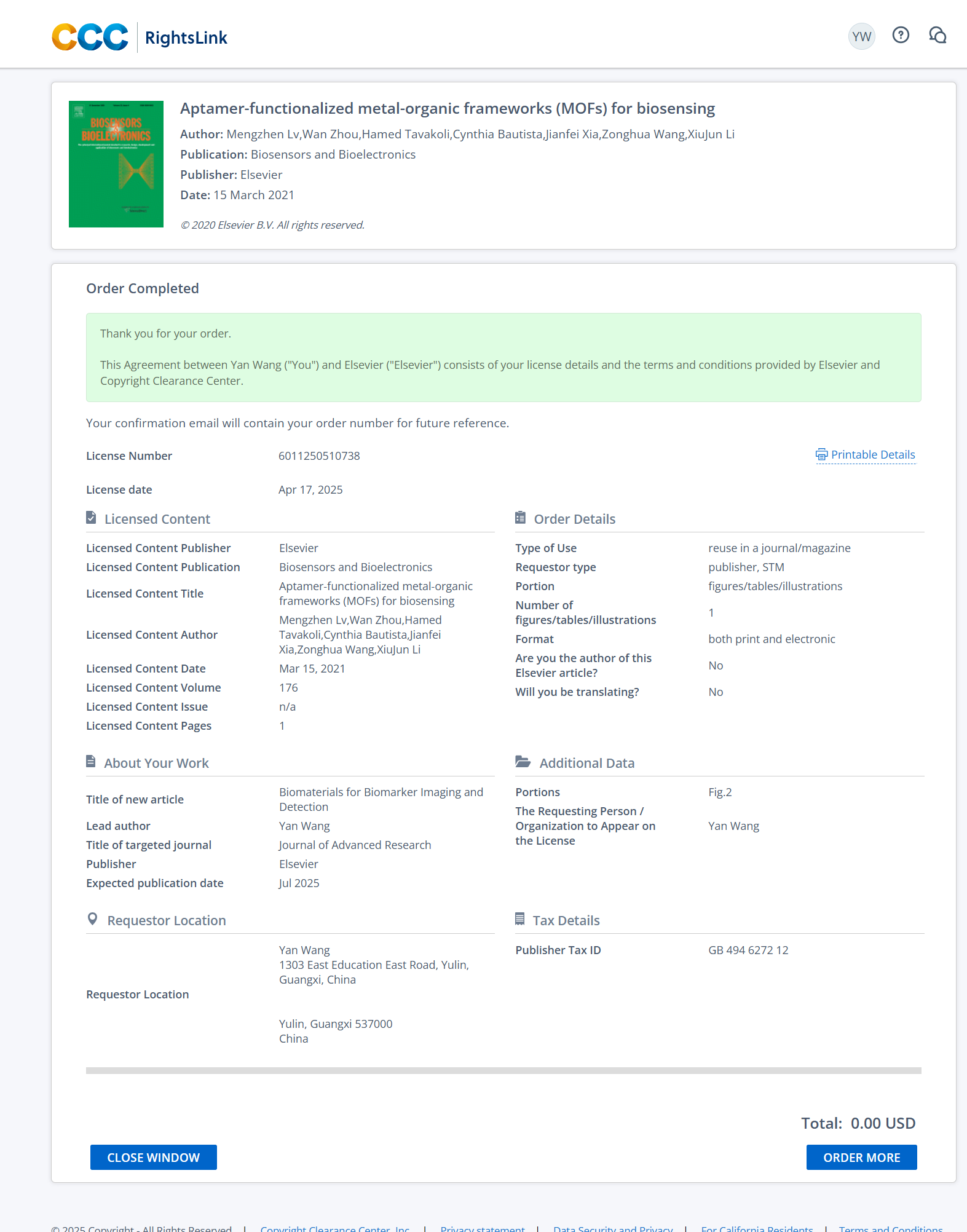

Supplement: Supplementary Data 25 [file mmc25.docx]

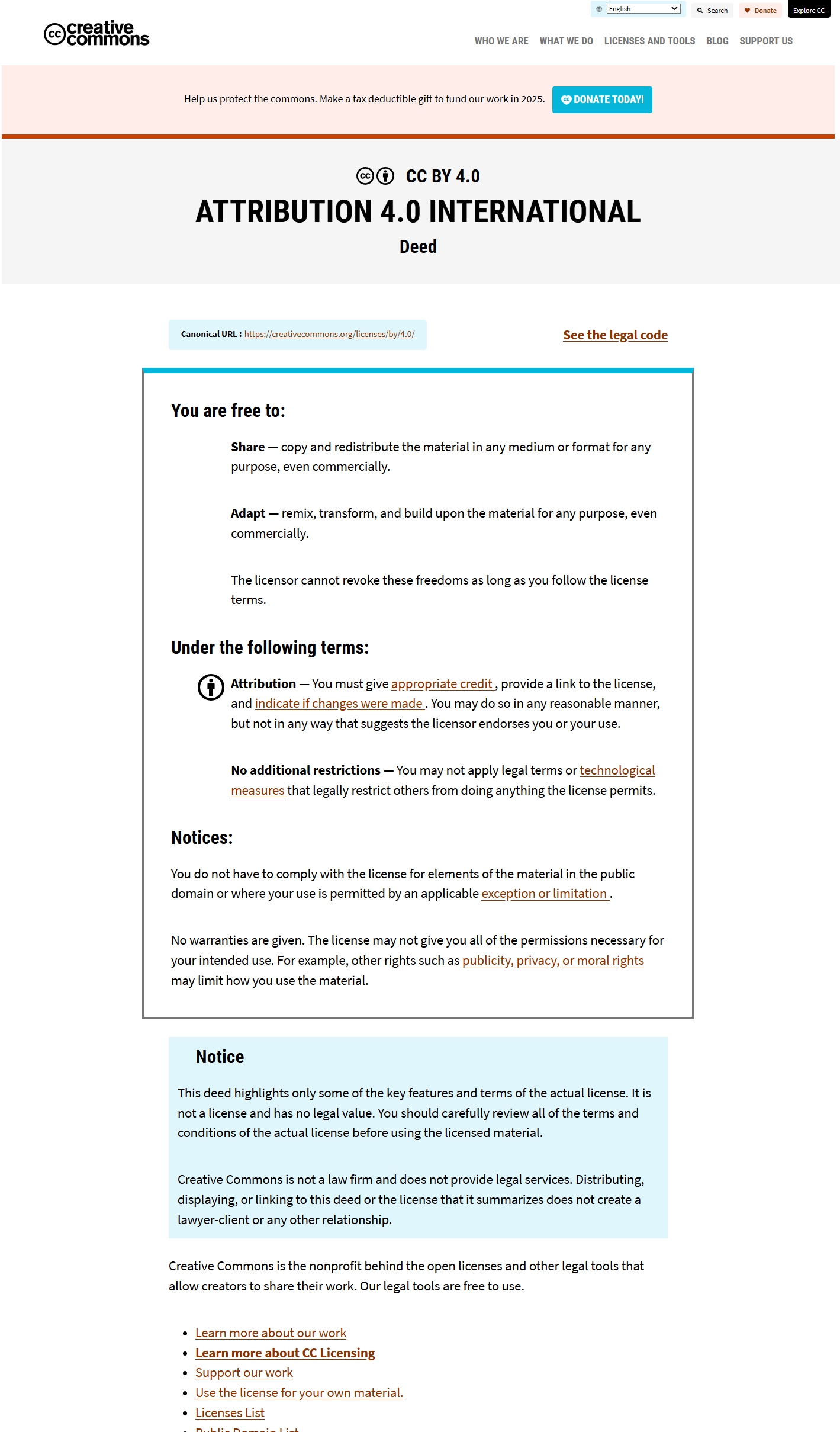

Supplement: Supplementary Data 26 [file mmc26.docx]
