# Supplementary material for: Biomaterials for biomarker imaging and detection
Source: J Adv Res. 2025 Aug 7;83:219–51. doi: 10.1016/j.jare.2025.07.049 (PMC13131447; doi:10.1016/j.jare.2025.07.049)
Supplement: Supplementary file 2 [file mmc1.docx]

Help us protect the commons. Make a tax deductible gift to fund our work in 2025. DONATE TODAY!

CC BY 3.0

ATTRIBUTION 3.0 UNPORTED

Deed

Notice

This is an older version of this license. Compared to previous versions, the 4.0 versions of all CC licenses are more user-friendly and more internationally robust . If you are licensing your own work , we strongly recommend the use of the 4.0 license instead: Deed - Attribution 4.0 International

Canonical URL : https://creativecommons.org/licenses/by/3.0/

See the legal code

You are free to:

Share — copy and redistribute the material in any medium or format for any purpose, even commercially.

Adapt — remix, transform, and build upon the material for any purpose, even commercially.

The licensor cannot revoke these freedoms as long as you follow the license terms.

Under the following terms:

Attribution — You must give appropriate credit , provide a link to the license, and indicate if changes were made . You may do so in any reasonable manner, but not in any way that suggests the licensor endorses you or your use.

No additional restrictions — You may not apply legal terms or technological measures that legally restrict others from doing anything the license permits.

Notices:

You do not have to comply with the license for elements of the material in the public domain or where your use is permitted by an applicable exception or limitation .

No warranties are given. The license may not give you all of the permissions necessary for your intended use. For example, other rights such as publicity, privacy, or moral rights may limit how you use the material.

Creative Commons is the nonprofit behind the open licenses and other legal tools that allow creators to share their work. Our legal tools are free to use.
